# An integrated peach genome structural variation map uncovers genes associated with fruit traits

**DOI:** 10.1186/s13059-020-02169-y

**Published:** 2020-10-06

**Authors:** Jian Guo, Ke Cao, Cecilia Deng, Yong Li, Gengrui Zhu, Weichao Fang, Changwen Chen, Xinwei Wang, Jinlong Wu, Liping Guan, Shan Wu, Wenwu Guo, Jia-Long Yao, Zhangjun Fei, Lirong Wang

**Affiliations:** 1 Zhengzhou Fruit Research Institute, Chinese Academy of Agricultural Sciences, Zhengzhou, China; 2 College of Horticulture & Forestry Sciences, Huazhong Agricultural University, Wuhan, China; 3 The New Zealand Institute for Plant & Food Research Limited, Private Bag 92169, Auckland, 1142 New Zealand; 4 Boyce Thompson Institute for Plant Research, Cornell University, Ithaca, NY USA; 5 US Department of Agriculture-Agricultural Research Service, Robert W. Holley Center for Agriculture and Health, Ithaca, NY USA

**Keywords:** SVs, GWAS, Peach, Fruit, Fruit shape

## Abstract

**Background:**

Genome structural variations (SVs) have been associated with key traits in a wide range of agronomically important species; however, SV profiles of peach and their functional impacts remain largely unexplored.

**Results:**

Here, we present an integrated map of 202,273 SVs from 336 peach genomes. A substantial number of SVs have been selected during peach domestication and improvement, which together affect 2268 genes. Genome-wide association studies of 26 agronomic traits using these SVs identify a number of candidate causal variants. A 9-bp insertion in *Prupe.4G186800*, which encodes a NAC transcription factor, is shown to be associated with early fruit maturity, and a 487-bp deletion in the promoter of *PpMYB10.1* is associated with flesh color around the stone. In addition, a 1.67 Mb inversion is highly associated with fruit shape, and a gene adjacent to the inversion breakpoint, *PpOFP1*, regulates flat shape formation.

**Conclusions:**

The integrated peach SV map and the identified candidate genes and variants represent valuable resources for future genomic research and breeding in peach.

## Introduction

Structural variations (SVs), comprising insertions/deletions (indels), duplications, inversions, and translocations, are widely present in genomes [1–4]. In humans, SVs provide an extensive source of genetic variation for the identification of genes involved in important biological processes [5]. In many plant species, SVs have been reported to regulate agronomic traits such as fruit shape in tomato (*Solanum lycopersicum*) [6], nematode resistance in soybean (*Glycine max*) [7], reproductive morphology in cucumber (*Cucumis sativus*) [8], asexual reproduction in citrus [9], and fruit texture in peach (*Prunus persica*) [10]. Recent rapid advances in next-generation sequencing technologies have facilitated genome-wide detection of SVs in large crop populations [11, 12].

Peach is the fourth largest deciduous fruit crop in the world (FAO, http://faostat.fao.org) and is regarded as a model plant for the Rosaceae family, thanks to its small genome and relatively short juvenile period [13]. Linkage analysis in peach has identified candidate genes for traits such as fruit flesh color [14], fruit hairiness [15], fruit flesh texture [10], double flower shape [16], and pendulous branches [17]; however, the genetic basis of these and other agronomic traits still remains largely unexplored. Genome-wide association studies (GWAS) represent an efficient method for mapping candidate genes and have been applied in peach to identify genome regions and/or genes associated with important traits, using genome-wide single nucleotide polymorphism (SNP) data [18–20]. However, identification of causal genes/variants is challenging in cases with large candidate regions. Furthermore, most GWAS analyses to date have been based on SNP data, while many phenotypes are associated with SVs. In peach, all reported genes that might be responsible for target traits have been associated with SVs [10, 15–17]. A pioneer study using SV data for GWAS in plants was reported in cucumber, where a large duplication controlling the reproductive morphology trait was detected [8]. Such an approach represents a more direct method for identifying candidate genes and casual variants, and the development of new bioinformatics methodologies, including more tools for analyzing SVs, has further enabled genome-wide SV mining.

Even though SVs are important sources of peach genetic diversity, their impact on genes and agronomic traits is still largely unknown. To date very few studies have focused on genome-wide detection of peach SVs [20], and subsequent investigations into the relationship between SVs and specific phenotypes using GWAS have not been reported. In this current study, 336 peach accessions were used to evaluate the SV landscape across the peach genome, resulting in an integrated SV map of more than 200,000 variants. GWAS of 26 agronomic traits were performed using the detected SVs in order to determine their significance and functional impacts.

## Results and discussion

### A peach sequence-based SV map

**Fig. 1:**
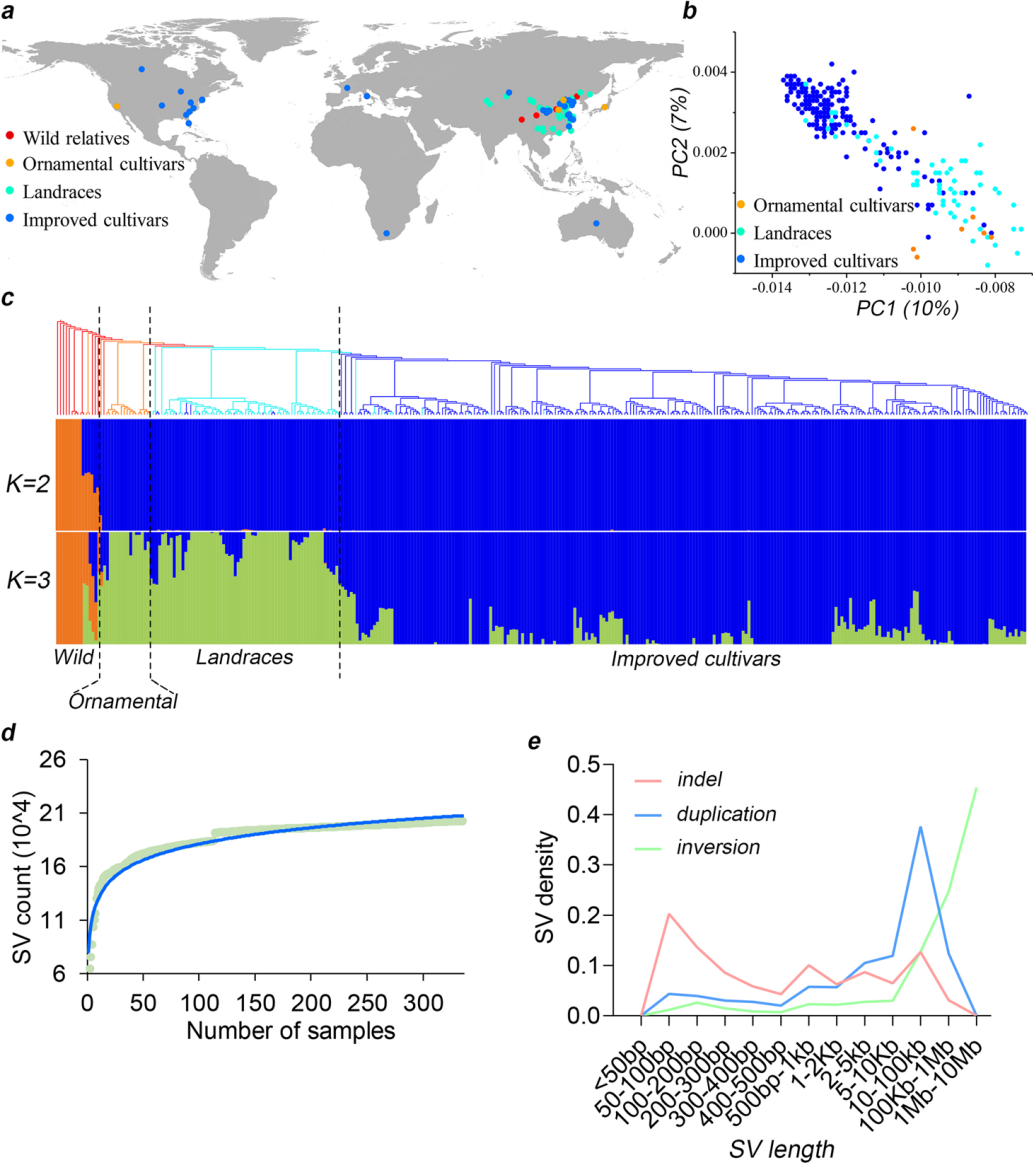
Phylogeny and structure variation in peach. **a** Geographic distribution of 336 peach accessions, represented by dots of different colors. **b** Principal component analysis (PCA) of ornamental, landrace and improved accessions using SVs. Different color dots represent the accession as in **a**. **c** Phylogenetic tree and population structure of 336 peach accessions using SVs. Different branch colors in the phylogenetic tree represent different groups, same as in **a**. **d** Simulations of the increase in number of SVs detected with the increase of accessions. The blue line is the fit curve of data points (light green). **e** SV density of different sizes for each SV type

A collection of 336 peach accessions, originating from all over the world (Fig. 1a), were re-sequenced to an average depth of 24× using the Illumina Hiseq X Ten platform (Additional file 1: Table S1). In order to improve the SV detection accuracy, we used four different tools that show high performance for human SV detection [21, 22]: LUMPY [23], Manta [24], GRIDSS [25], and Delly [26]. To further reduce the false-positive rate, SVs detected by LUMPY were filtered by setting a threshold for quality value (> 20) [21] and number of supporting reads (> 5). Deletions smaller than 340 bp lacking split read support were removed, and only variants detected by at least two tools were kept for the downstream analyses.

A final set of 202,273 SVs, ranging from 50 bp to 10 Mb, were identified among the 336 peach genomes, including 121,527 indels, 10,728 duplications, 8336 inversions, and 61,682 translocations (Additional file 1: Table S2). Based on these identified SVs, the peach accessions studied here could be divided into three major groups, ornamental cultivars and landraces as one group, the improved cultivar group, and the wild group (Fig. 1b, c, Additional file 2: Fig. S1). Phylogenetic relationship of the accessions inferred from these data was largely consistent with that reported in previous studies inferred using SNPs [18–20]. The total number of SVs from 150 randomly selected accessions was 193,575, accounting for 96% of the total SV number in all 336 accessions. Modeling of the SV size by iteratively randomly sampling accessions indicated that the SV number was relatively finite in the peach population (Fig. 1d), suggesting that our SV detection was comprehensive and nearly complete. Most of the detected SVs were present in a few accessions, and a total of 131,075 SVs had a minor allele frequency (MAF) < 0.01 (Additional file 2: Fig. S2). Most indels and duplications ranged in size from 100 bp to 10 kb, while many inversions were > 10 kb (Fig. 1e, Additional file 1: Table S2).

Short length of reads generated using next-generation sequence (NGS) technologies and limitations of current computational algorithms can restrain the accuracy of SV detection [27, 28]. To assess the accuracy of the SV calling in this study, six of the 336 accessions were sequenced using PacBio single molecule real-time sequencing (SMRT) technology [29]. A list of 150 SVs were randomly selected. A manual check by comparing these SVs with the PacBio long read mapping results using the IGV program [30] revealed an accuracy rate of 88% (Additional file 1: Table S3).

### Functional impact of peach SVs

SVs are known to have a major influence on genomes and are often associated with specific traits [5]. We investigated the associations of the SVs identified here with gene, coding sequence (CDS), and promoter regions. A total of 26,361 out of 26,873 genes (98%) were associated with at least one SV among the 336 peach genomes (Additional file 1: Table S4). These included 20,721 in CDSs, 3505 in introns, and 2135 in promoter regions. The 512 unaffected genes in the peach genome were mostly annotated as related to fundamental biological processes, such as chloroplast function (Additional file 2: Fig. S3, Additional file 1: Table S4), indicating genome regions regulating these processes have been fixed during peach evolution. This result indicated that SVs have a major role in gene regulation and morphological variation. This phenomenon has also been reported in other pan-genome studies [31, 32], where genes that were not present in the reference genome sequence were identified due to the presence of SVs.

**Fig. 2:**
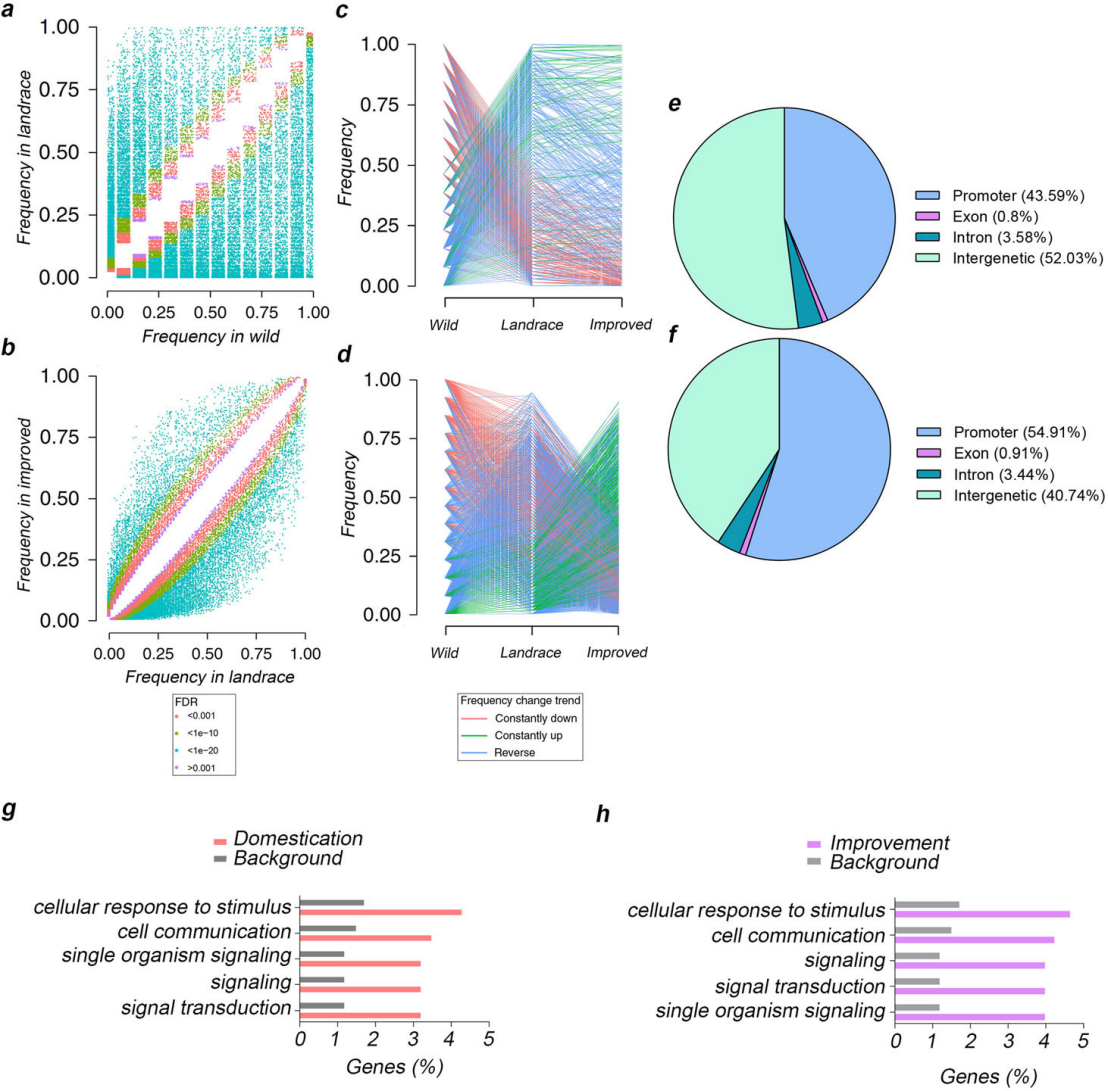
Functional impacts and distribution of peach SVs. **a**, **b** Scatter plots showing SV occurrence frequencies in wild and landrace groups (**a**) and in landrace and improved groups (**b**). **c**, **d** Occurrence frequency patterns of putative selected SVs during peach domestication (**c**) and improvement (**d**). **e**, **f** Impacts of selected SVs on genome during peach domestication (**e**) and improvement (**f**). **g**, **h** GO enrichment analysis of genes affected by SVs selected during peach domestication (**g**) and improvement (**h**)

During peach domestication and improvement, many genetic loci have been selected, and a number of studies have characterized this phenomenon using SNP data [19, 20]. In this study, a total of 134 domestication and 97 improvement sweeps, covering 10.3% (22.9 Mb) and 8.7% (20.0 Mb) of the genome, respectively, were identified using SNPs, while a total of 210 domestication and 170 improvement sweeps, covering 13.6% (31.3 Mb) and 12.1% (28.0 Mb), respectively, were identified using SVs (Additional file 2: Fig. S4, Additional file 1: Table S5–8). There were only 11.7 Mb overlapping sweeps for domestication and 11.7 Mb for improvement between those identified using SNPs and SVs (Additional file 1: Table S9–10). Therefore, to evaluate the impact of SVs on domestication and improvement processes, we selected specific SV sites based on their occurrence frequency and calculated and compared the occurrence frequencies for each SV in wild, landrace, and improved accession groups. Among all the detected SVs, 25,416 showed evidence of positive selection during domestication, with occurrence frequencies significantly higher in the landrace group than in the wild, while 29,780 showed evidence of negative selection (Fig. 2a, b, Additional file 1: Table S11). During the improvement process from landrace to modern cultivars, 4994 and 22,277 SVs were positively and negatively selected, respectively (Fig. 2c, d, Additional file 1: Table S11). The putative functional impact of these SVs was examined, and < 5% were found to be located in gene regions (Fig. 2e, f), affecting 2123 genes in the domestication process and 1207 genes in the improvement process. Gene ontology (GO) enrichment analysis showed that genes involved in cellular response to stimulus and signaling were significantly overrepresented (Fig. 2g, h), which is in agreement with a similar study in tomato [31]. A total of 1093 genes showed evidence of positive selection and 1432 of negative selection during domestication, with the corresponding numbers being 275 and 1023, respectively, during the improvement process (Additional file 1: Table S11). In total, 2268 genes have been selected during peach evolution, with 1059 specifically during domestication, 145 specifically during improvement, and 1064 during both (Additional file 2: Fig. S5).

### Genome-wide association studies of 26 agronomic traits

To further characterize functions of the SVs in peach, GWAS were performed for 26 key agronomic traits, including 18 traits for which GWAS has not been previously reported in peach. Performing GWAS with SNPs is an established methodology and one that takes advantage of the fact that SNPs provide a much higher density of polymorphisms than SVs and can be mapped to a relatively accurate genomic interval [9, 19, 33–35]. However, only a few studies in plants to date have described GWAS using SVs [8, 32]. There is considerable evidence from peach genetic and molecular studies that SVs can cause major phenotypic variance [10, 15–17], suggesting that SVs may represent a valuable source of variation for GWAS in peach. In order to characterize causal SVs, GWAS were performed separately using SNPs and SVs (including small variations < 50 bp) with MAF > 0.01. A total of 71,198 large SVs (> 50 bp) combined with 109,067 small indels (< 50 bp) with MAP > 0.01 were used for GWAS. The highly associated regions identified with SVs were largely consistent with those identified using SNPs and were also largely consistent using different models (Additional file 2: Fig. S6–31). In this study, GWAS provided reliable candidate variants that were used for gene identification in the context of these 26 agronomic traits (Additional file 2: Fig. S6–31, Additional file 1: Table S12).

### GWAS using SVs provide a powerful approach for identifying candidate genes

We selected 8 qualitative traits that have been targeted in previous GWAS analyses using SNPs [19, 20] and performed GWAS for them using both SNPs and SVs. We found that the most significant association signals were almost in the same genome regions between the two datasets; however, GWAS using SV data link traits to large genome variations, which are more likely to alter gene functions. For the 11 qualitative traits analyzed in this study, candidate genes have been previously found for four of them, namely fruit hairiness [15], flesh color [14], double flower shape [16], and pendulous branches [17], and we also identified these candidate causal DNA variations using GWAS with the SV data (Additional file 2: Fig. S7, S8, S10, S12, Additional file 1: Table S12). A transposable element (TE) insertion in a *MYB* gene (*Prupe.5G196100*) was previously reported to be associated with the fruit hairiness trait, resulting in the peach and nectarine phenotypes [15], and this variation was also found to be the most significant signal in the GWAS with SV data (Additional file 2: Fig. S7). For the fruit flesh color trait, three variations in the *CCD4* gene (*Prupe.1G255500*) on chromosome 1 have been implicated: a TE insertion, a simple sequence repeat (SSR), and a SNP [14]. Since multiple loci contribute to this trait, GWAS is unlikely to identify all three variants at the same time, and we found the most significant signal to be the SSR locus (Additional file 2: Fig. S8). When checking for variants located in this gene in 279 accessions with known flesh color phenotypes, we also found that these three variants were associated with the flesh color trait. Finally, a 1.3-kb deletion in *Prupe.3G200700* was found to be related to the pendulous branch trait (Additional file 2: Fig. S12, Additional file 1: Table S12). However, since this locus was not the most significant signal in the GWAS analysis, and some accessions with a weeping phenotype did not contain this variation, indicating that other variants responsible for the pendulous branch trait might exist. Taking all these into account, we concluded that GWAS using SVs provides an efficient strategy to identify candidate genes and in some cases can outperform the traditional GWAS with SNP approach, in agreement with studies of rapeseed (*Brassica napus*) [32].

There is considerable evidence that SVs control many phenotypic traits [28, 36, 37], and GWAS represents a powerful tool for mapping genes in genetic and molecular biology studies [19, 34, 35, 38, 39]. Due to the reliable detection of SNPs, GWAS using SNPs has been widely used; however, there are only a few studies showing that a single SNP can change gene function and the phenotype. In peach, only one trait (flesh color, white/yellow) was reported to be controlled by a SNP, while the flesh color trait was also controlled by two other non-SNP variations [14]. All other reported traits in peach were regulated by SVs. In our study, GWAS were performed using SVs, resulting in a map that was similar to that from GWAS using SNPs, but GWAS with SVs here allowed a more accurate prediction of causal variants that might be responsible for traits.

### A 9-bp insertion in a gene coding region leads to early fruit maturity

**Fig. 3:**
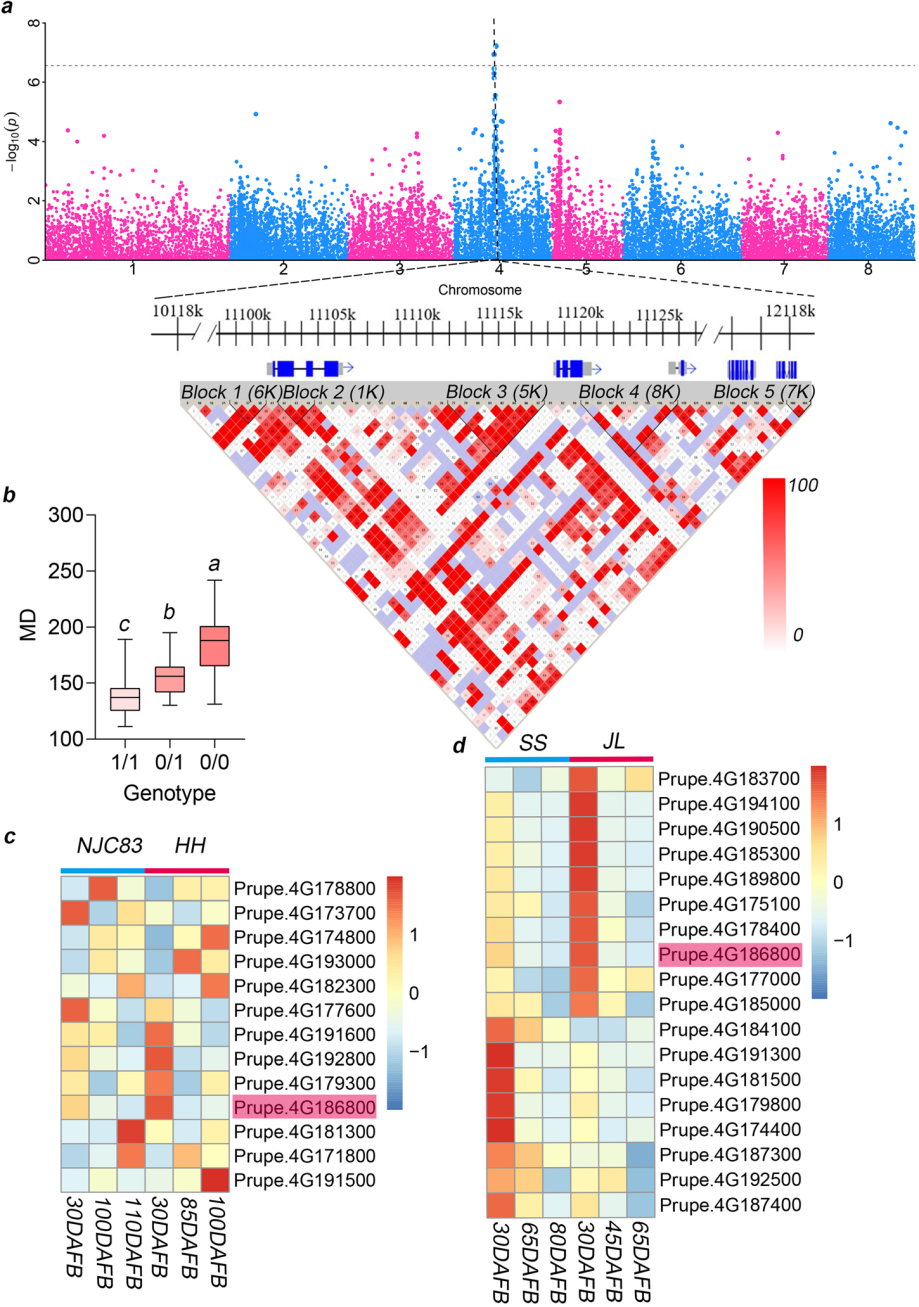
Identification of the gene underlying the fruit mature date (MD) trait. **a** Manhattan plot of MD using GWAS with SVs (top) and LD plot of the 200-kb region surrounding the most significant signal (bottom). Color for each box in LD plot represents LD relationship, showing increasing LD from white to red color. The number in each box represents the LD value multiplied by 100. **b** Allelotypes of the 9-bp insertion in *Prupe.4G186800* identified in 136 peach accessions and their relationship with MD. In each bar plot, the black line in the box indicates the mean value, and the lower and upper bounds of the box indicate the first and third quartiles, respectively. Groups labeled with different letters indicate significant difference at *P* < 0.01. **c**, **d** Expression patterns during fruit development of differentially expressed genes (DEGs) in the 200-kb region surrounding the most significant signal. The “JL” cultivar is a bud mutation of cultivar “SS” and its fruit matures 15 days earlier than “SS”. The “HH” cultivar is a bud mutation of cultivar “NJC83” and its fruit matures 10 days earlier than “NJC83.” The candidate gene, *Prupe.4G186800*, is highlighted in pink

Fruit maturity date (MD) is a critical factor in fruit marketing, and previous studies using linkage analysis have placed the MD locus on linkage group 4 in peach [40, 41]. A possible candidate gene, *Prupe.4G186800*, encoding a NAC transcription factor, was reported to be responsible for the MD trait in peach, and a 9-bp insertion was identified as the possible causal variant [41]. Consistently, this insertion in the coding region of *Prupe.4G186800* was also detected as the most significant signal in the GWAS analysis (Fig. 3a); however, *Prupe.4G186800* and *Prupe.4G186900* (annotated as encoding a protein with unknown function) were in the same linkage disequilibrium (LD) block (Fig. 3a). By genotyping this variant in the population, the accessions could be divided into three groups: early MD, which contains a homozygous insertion (1/1); intermediate MD, with a heterozygous genotype (0/1); and late MD, with no insertion, which is the same genotype as the reference (0/0) (Fig. 3b). To validate the functional role of *Prupe.4G186800* in controlling MD and given the transgenic limitations in peach, the cultivar “Spring Snow” (SS) and its bud mutant “Jinlei” (JL), whose fruit matures 15 days earlier than “SS,” and the “NJC83” cultivar and its bud mutation “Huihuang” (HH), whose fruit matures 10 days earlier, were subjected to comparative transcriptome analysis of fruit development. The differentially expressed genes (DEGs) in a 200-kb genome interval around the candidate region were analyzed, and *Prupe.4G186800* was the only gene with much higher expression in both bud mutants (Fig. 3c, d). During fruit development, this gene showed much higher expression levels in early stages (Fig. 3c, d). In addition, this locus also provided the most significant signal in GWAS for the fruit development period trait (Additional file 2: Fig. S21). This finding suggested that fruit MD and the fruit growth period trait are correlated. In summary, we confirmed that MD located in linkage group 4 is a major MD locus and that *Prupe.4G186800* is a candidate gene and the 9-bp insertion in *Prupe.4G186800* is a candidate causal variant for early fruit maturity and fruit growth control.

### Gain of anthocyanins in flesh surrounding the fruit stone

**Fig. 4:**
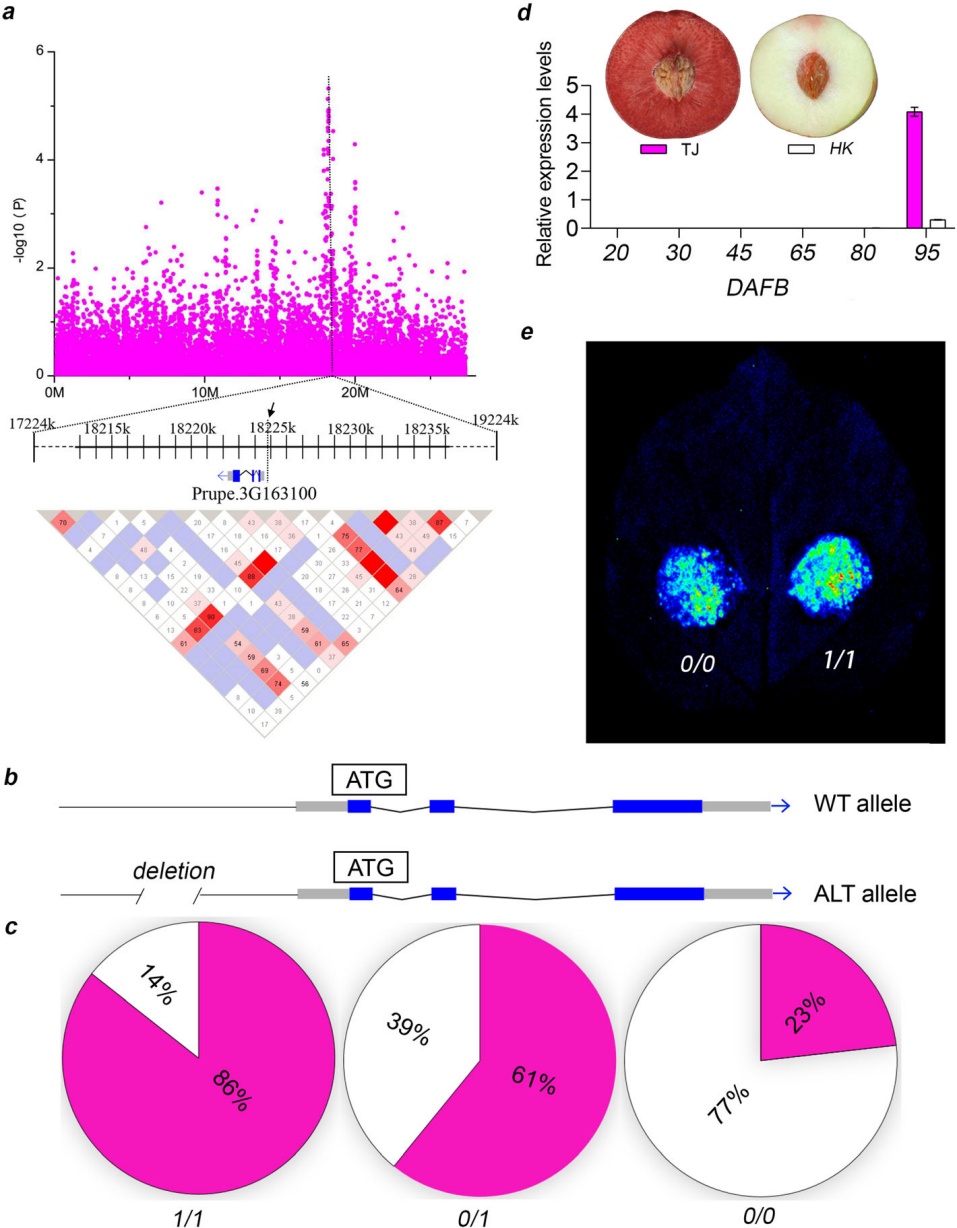
GWAS of flesh color around the stone. **a** Manhattan plot of flesh color around the stone on chromosome 3 (top) and LD plot of the 200-kb region surrounding the most significant signal. The dotted black line indicates the highest signal for this trait. Color for each box in LD plot represents LD relationship, showing increasing LD from white to red color. The number in each box represents the LD value multiplied by 100. **b** Two different alleles of *PpMYB10.1*. WT allele stands for the reference allele, while the ALT allele is the alternate allele that has a 487-bp deletion (DEL) in its promoter. **c** Allelotypes of the deletion in the promoter of *PpMYB10.1* observed in 295 peach accessions and their relationship with flesh color around the stone. The white color represents no red color around the stone, while the red-pink color stands for red color around the stone. **d** Relative expression of *PpMYB10.1* during fruit development determined by qRT-PCR. “TJ” is a red-fleshed cultivar and “HK” is a white-fleshed cultivar. The fruit ripens at 95 days after full bloom (DAFB). **e** Promoter activity analysis by dual-luciferase assay. 0/0 stands for the reference promoter sequence, while 1/1 represents the promoter with a 487-bp deletion

Anthocyanin accumulation is an important fruit attribute, and candidate genes for anthocyanin-related fruit traits have been reported in peach, including flesh color [42, 43] and fruit skin color [44]. The main candidate gene for fruit skin color is *PpMYB10.1*, while more than one gene, but including *PpMYB10.1*, have been associated with regulating flesh color [42, 43]. The presence of one signal for fruit skin color and many for flesh color was also noted here (Additional file 2: Fig. S32a, b). However, the underlying mechanism determining flesh color around the stone is still unknown. From the GWAS analysis using SV data, a 487-bp deletion affecting the *PpMYB10.1* promoter region was found to be associated with this trait (Fig. 4a, b), and the deletion genotype was highly correlated with the red flesh around the stone phenotype (Fig. 4c). To investigate the potential contribution of *PpMYB10.1* to the color formation in the flesh surrounding the stone, its expression was analyzed and found to correlate with the occurrence of anthocyanins in the corresponding flesh region (Fig. 4d). It has been reported that the expression of *PpMYB10.1* is regulated by the BL transcription factor [38]; however, the effect of the deletion on the promoter activity of *PpMYB10.1* remains unknown. In a dual-luciferase reporter assay, we found that the deletion enhanced the promoter activity, consistent with a role of *PpMYB10.1* in flesh color formation around the stone (Fig. 4e). In addition, the deletion in the *PpMYB10.1* promoter was found to have been selected for during domestication, with a significantly higher occurrence frequency in landraces than in wild accessions (Additional file 1: Table S11).

### A large inversion co-segregates with flat fruit shape

**Fig. 5:**
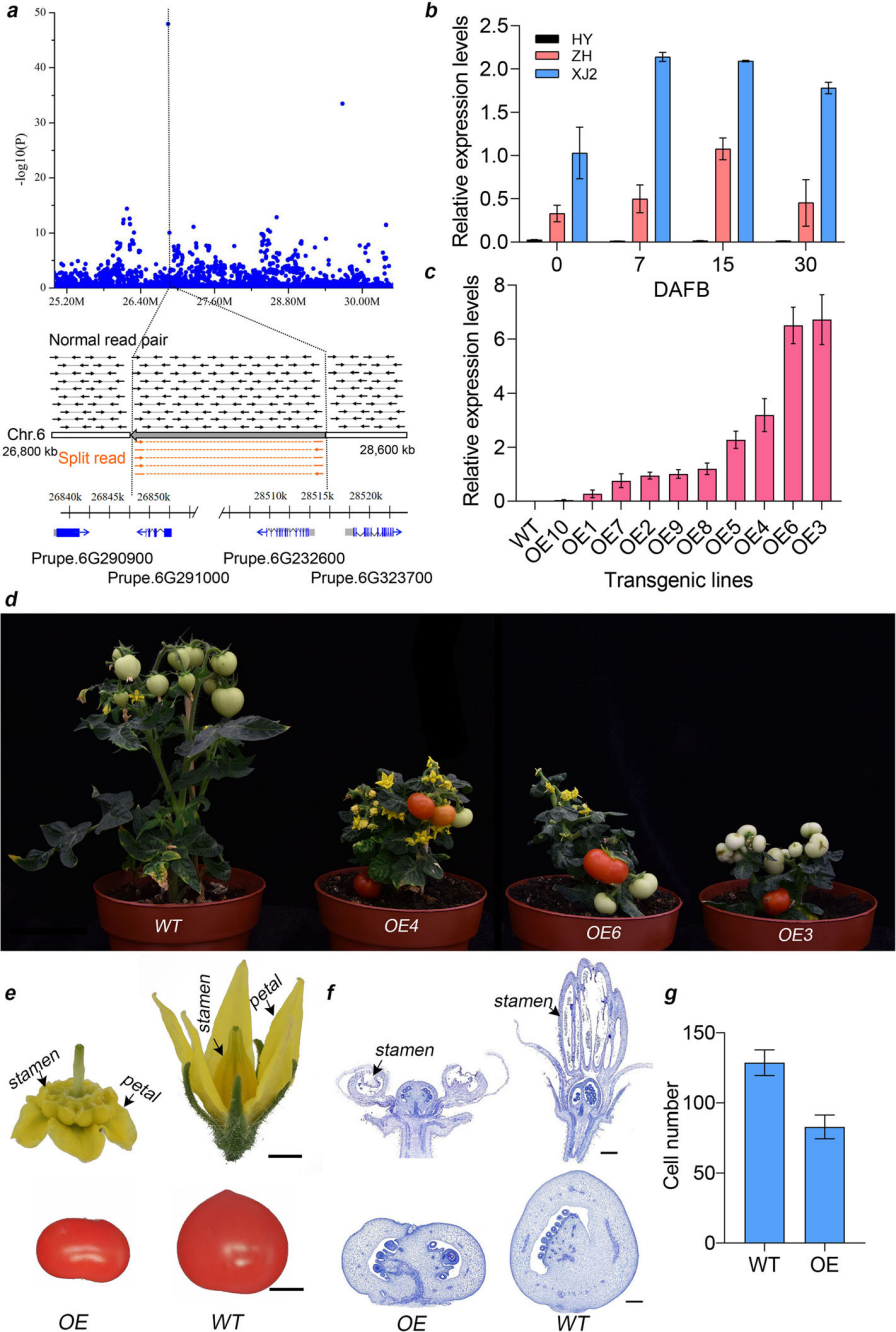
Identification and functional characterization of the candidate fruit shape gene. **a** A 1.67-Mb inversion highly associated with fruit shape. Top, Manhattan plot of GWAS of fruit shape. Bottom, the inversion supported by read alignments. Black arrows connected with solid lines represent normal paired-end reads mapped against the peach genome and orange arrows connected with dotted lines indicate split reads mapped against the breakpoints of the inversion. **b** Relative expression levels of *PpOFP1* (*Prupe.6G290900*) during early fruit development in normal flat (ZH), aborted flat (XJ2) and round peach (HY). **c** Relative expression levels of *PpOFP1* in transgenic tomato lines determined by qRT-PCR. **d** Phenotypes of transgenic Micro-Tom tomato lines overexpressing peach *PpOFP1* gene. Scale bar = 5 cm. **e** Phenotypes of flower and fruit collected from wild type (WT) and overexpressing (OE) transgenic tomato. Scale bar = 0.5 cm. **f** Paraffin sections of flowers (top) and fruit 10 days after fertilization (bottom) in WT and OE transgenic tomato. Scale bar = 500 μm. **g** Cell number along the vertical axis of WT and OE tomato fruit at 10 days after fertilization

Fruit shape is an important trait and determinant of consumer selection. Peach fruit have two typical shapes, flat and round, which are controlled by a single dominant gene, *S* [45]. Genetic markers closely linked to the flat fruit trait have been identified and are useful for marker-assisted breeding; however, the specific mechanisms controlling flat shape formation in peach are still unknown. In this study, GWAS was performed for fruit shape using SV data and a 1.67-Mb inversion was identified (Fig. 5a) that co-segregated with the flat fruit shape. All flat peach accessions that could produce mature fruits were heterozygous at this inversion locus, while fruit of the homozygous genotype aborted during early development. In this study, 34 of 35 flat peach accessions were heterozygous and one was homozygous, while all the 301 round peach accessions were homozygous, with the same genotype as the reference at this locus. To verify the accuracy of this inversion call, PacBio long reads spanning the breakpoints were identified, and meantime, a draft genome was assembled from PacBio long reads and contigs that spanned the inversion interval were identified from the assembly as well. The presence of the inversion was further confirmed by examining the alignments of raw reads (Additional file 2: Fig. S33a) and assembled contigs (Additional file 2: Fig. S33b), respectively, to the peach reference genome. We also validated this inversion in cultivars and an F1 population (a cross of “Okubo” × “You Pan Tao 1-3”) using PCR-based method (Additional file 2: Fig. S34).

This inversion covers the SNP associated with flat shape reported in a previous study [19] and is approximately 500 kb away from the 10-kb deletion reported to co-segregate with the flat shape [46] (Additional file 2: Fig. S35). Based on its location, we deduced that the inversion might affect four genes, *Prupe.6G290900*, *Prupe.6G291000*, *Prupe.6G323600*, and *Prupe.6G323700*, which were located around the two breakpoints (Additional file 2: Fig. S35). To determine the best candidate gene, we performed quantitative real time-PCR (qRT-PCR) to measure the relative expression patterns of the four genes during fruit development in “JH” (round peach), “HY” (round peach), “ZPT10” (normal flat peach), “ZH” (normal flat peach), and “XJ2” (aborting flat peach). We found that the expression patterns of two of the genes, *Prupe.6G290900* and *Prupe.6G323700*, were related to the allelic genotypes of flat and round peach (Fig. 5b, Additional file 2: Fig. S36), indicating that they might be responsible for flat fruit shape formation. Given that the flat shape gene is dominant, the candidate gene with higher expression levels in flat peaches was more likely to be the gene responsible for the flat phenotype, as was the case for *Prupe.6G290900*. Notably, *Prupe.6G290900* was annotated as encoding an ovate family protein (OFP), a member of which is the key gene controlling fruit shape in tomato [47], and we named this gene *PpOFP1*. Phylogenetic analysis of OFP genes in peach and tomato showed a close relationship between *PpOFP1* and *SlOFP20*, a gene known to control tomato fruit shape (Additional file 2: Fig. S37).

Stable transformation of peach is not yet technically possible and so we could not confirm the function of *PpOFP1* by generating transgenic peach lines. As an alternative approach, we expressed *PpOFP1* in the Micro-Tom tomato genotype. Ten transgenic lines with different levels of *PpOFP1* expression were generated (Fig. 5c). Three transgenic lines, *OE3*, *OE4*, and *OE6*, expressed *PpOFP1* to a medium to high levels and exhibited developmental aberrations. They were shorter than the WT control plants and most importantly produced flat tomato fruit (Fig. 5d, e). These lines also showed abnormal floral organs such as stamen and stigma (Fig. 5e, f). The stigma phenotype was similar to that of the flat peach stigma, which is shorter and thicker than those of round peach (Additional file 2: Fig. S38). According to paraffin section result, the cell number along the vertical axis in transgenic flat tomato fruit was lower than that in round tomato fruit (Fig. 5f,g), consistent with the development of flat peach [48].

## Conclusions

In this study, we provided an integrated map of genome SVs by re-sequencing 336 peach accessions, collectively originating from all over the world. We analyzed the putative effects of the SVs on the genome during peach domestication and improvement and found that almost all genes in peach were affected by SVs and that the very few unaffected genes were almost all involved in core biological processes. A GWAS approach using SVs was found to be more efficient than GWAS using SNPs in identifying candidate genes and causal variants, and based on the SV dataset we generated here, we performed GWAS for 26 peach agronomic traits. This suggested candidate genes responsible for the fruit related traits, such as flesh color around the stone, fruit MD, and fruit shape. The function of *PpMYB10.1* was observed to include control of flesh color around the stone, while the candidate gene for fruit MD was confirmed to be *Prupe.*4G186800, as previously reported. The fruit shape candidate gene, *PpOFP1*, was validated in transgenic tomato, and its heterologous expression in tomato leads to a flat fruit. The integrated SV map provides a valuable resource for future genomic research in peach and other plant species. In addition, the significant association signals identified for the 26 agronomic traits provide valuable candidates for the genetic improvement of peach and will be beneficial to the peach industry.

## Materials and methods

### Plant materials and genome resequencing

In this study, 336 peach accessions were sampled from National Peach Germplasm Repository of China (NPGRC, Zhengzhou), including 13 wild, 20 ornamental, 70 landrace, and 233 improved accessions (Additional file 1: Table S1). In these samples, 104 were reported in our previous study [20, 49]; however, the sequencing depth of these accessions was ~ 5×, which would not be suitable for SV detection [23]. We therefore performed further genome resequencing of these accessions to an average depth of ~ 20×. The genomic DNA was extracted from young leaves as previously described [50]. DNA libraries were constructed with an insert size of ~ 350 bp and sequenced on a Hiseq X Ten platform (Illumina) which generated paired-end reads of 150 bp in length. For each accession, no less than 5 Gb of sequencing data was generated to ensure the reliability of SV detection.

### Phenotyping

Twenty-six agronomic traits were targeted in this study, including 11 qualitative and 15 quantitative traits. The 11 qualitative traits, including fruit shape, fruit hairiness, flesh color (white/yellow), flesh color around the stone, flower shape (double/single; showy/non-showy), pendulous branch, pollen sterility, hypanthium color (white/yellow), anther color, and kernel taste, were measured in two successive years according to previous reported evaluation criteria [51] from 2011 to 2012. The 15 quantitative traits, including internode length, flower/leaf bud ratio, relative height between pistil and stigma, suture depth, fruit development period, development period, leaf length, leaf width, bloom date, full bloom date, bloom ending date, leaf expanding date, fruit maturity date, deciduous date, and deciduous ending date, were recorded in 1 to 3 years as shown in Additional file 1: Table S1.

### SV detection and genotyping

Raw Illumina reads were processed to remove adapter and low-quality sequences. The paired-end reads were mapped to the reference peach “Lovell” genome [52] (release version 2.0_a2.1) using BWA [53] (version 0.7.15) with the following parameters: “bwa mem -M -R.” Four tools were selected for SV detection: LUMPY [23] (version 0.2.13), Manta [24] (version 1.6.0), GRIDSS [25] (version 2.5.2), and Delly [26] (version 0.7.8). LUMPY was used to detect SVs, with the exception of insertions, with the following parameters: “lumpyexpress -P -B -S –D.” To further reduce the number of false SVs, we filtered deletions that were < 340 bp and had no split read support. The SV results were then genotyped in the population using SVTyper [54] (version 0.0.4). For the other three tools, SVs were detected and genotyped using the default parameters, as these tools can be used to both detect SV and perform genotyping. To improve the accuracy, the results were filtered using SURVIVOR [55] (version 1.0.6) by keeping the variants that were detected by at least two tools, with the following parameters: “SURVIVOR merge name 1000 2 1 1 0 50.” SURVIVOR defines and merges SVs according to the distance between breakpoints, SV type and SV strands. In this study, we set the maximum distance between breakpoints to 1000 bp. Transposons were identified with panISa [56] and genotyped according to the number of clipped reads and sequencing depth at the variants position using an in-house script.

### Small variants calling

SNP and small indel calling were performed using the GATK Best-Practices pipeline (https://software.broadinstitute.org/gatk/best-practices/). The detection was performed using the GATK HaplotypeCaller, and genotyping was done with GenotypeGVCFs [57], and the separation of SNPs and indels was performed using the GATK selectTypeToInclude option. After separation, the SNP call set was filtered by applying the following parameters in GATK VariantFiltration to ensure accuracy: “QUAL <40, QD <2.0, FS >60.0, MQ <40.0, MQRankSum <-12.5, ReadPosRankSum <-8.0.” The indel (< 50 bp) set was filtered with parameters: “QD <2.0, FS >200.0, ReadPosRankSum <-20.0.”

### Quality evaluation of the detected peach SV set

To evaluate the accuracy of the detected SVs, we randomly selected 150 variants using the Linux command “shuf -n.” In addition to Illumina short reads, single-molecule real-time sequencing (SMRT) was used to generate PacBio long reads for six accessions: “Xinjiang Pan Tao #2,” “Hakuho,” “Tianjin Shui Mi,” “Ying Ge Tao,” “Okubo,” and “Tian Ren Tao.” The PacBio reads were mapped to the reference peach genome using NGMLR [58] (version 0.2.7). Based on the SV type, genome position and SV size, we manually checked the short-read mapping results and the PacBio long-read mapping results using IGV [30] to evaluate the accuracy of SVs.

### Phylogeny, population structure, and selective sweep analyses

A maximum-likelihood phylogenetic tree was constructed based on the binary SV data using IQ-TREE [59] with 1000 bootstraps. Using the same binary SV data, population structure was investigated using ADMIXTURE [60], and principal component analysis (PCA) was performed using the smartpca program of the EIGENSFOT [61] software (version: 6.0.1) with default settings. Nucleotide diversity (π) was calculated for each group with VCFtools [62] (version 0.1.12) using a window size of 100 kb and a step size of 10 kb. The selective sweeps during domestication and improvement were identified based on reduction of diversity (ROD). The π_wild_/π_landrace_ and π_landrace_/π_improved_ values were calculated and the top 5% windows with the highest ROD values were identified as selective sweeps during domestication and improvement processes, respectively.

### Functional impact of SVs and SV selection during peach evolution

According to the genome annotation, we assessed the putative SV impacts on gene, CDS, and promoter regions according to SV locations and sizes. We set an interval of 2 kb upstream of the gene as the promoter region. To evaluate the SV significance in peach evolution, the occurrence frequencies of each SV were calculated in wild, landrace and improved groups. SVs with higher or lower occurrence frequencies in landrace compared to wild, and improved compared to landrace, were designated as showing positive or negative selection, respectively, during domestication or improvement. The significance of the difference of the frequencies for each SV between the two compared groups (wild versus landrace for domestication and landrace versus improved for improvement) was determined using the Fisher exact test with the R package “pdrtool.” The resulting raw *P* values of all SVs in each of the two comparisons were then corrected based on a false discovery rate (FDR). SVs with significantly different frequencies (FDR < 0.001 and fold change > 2) were identified as those under selection. These SVs were then analyzed to identify the associated genes, and GO enrichment analysis was performed for these genes using the AgriGO [63] with a cutoff of FDR < 0.05.

### GWAS for 26 agronomic traits

To identify candidate genes responsible for the various agronomic traits, GWAS were performed for 26 agronomic traits. We aimed to identify variants associated with these traits by performing GWAS with the SV dataset and the SNP dataset, separately. To improve the accuracy of the GWAS results, we filtered the SV and SNP datasets by removing those with minor allele frequency (MAF) < 0.01. GWAS were performed using MLM, CMLM, and FarmCPU models implemented in GAPIT [64] (version 3.0), which also integrates PCA and kinship analyses. The significance cutoff was defined as the Bonferroni test threshold, which was set as 0.05/(total number of SVs) and 0.05/(total number of SNPs), which corresponded to -log10(P) = 6.56 for SVs and 7.82 for SNPs.

### Experimental validation of the inversion variation in the *S* locus

An inversion of 1.67 Mb was found to co-segregate with the flat shape in peach. To validate this inversion, first, PacBio SMRT library was constructed and sequenced for the peach accession, Xinjiang Pan Tao #2, which was homozygous at the *S* locus. The resulting PacBio long reads were then mapped against the peach reference genome using NGMLR [58] and reads covering the inversion were identified according to the inversion location. Second, the PacBio reads were assembled de novo using Canu [65] (version 1.8) to obtain long contigs that might cover the inversion locus. After assembly, the collinearity between the assembled Xinjiang Pan Tao #2 and the reference genomes was plotted using MUMmer [66] (version 3.9.4), and based on the dotplot around the *S* locus, the inversion event was evaluated. Finally, we confirmed the inversion by PCR using primers (Additional file 1: Table S13) adjacent to the inversion breakpoints.

### RNA extraction and real-time PCR analysis

To investigate the fruit maturity and flesh color around the stone traits, fruit samples were collected at five stages from “Hakuho” (HK; white flesh) and “Tianjin Shui Mi” (TJ; red flesh): 20, 40, 60, 80, and 90 DAFB (days after full bloom). For the fruit shape trait, fruit samples were collected at 16 stages from pre-bloom bud to fruit maturation period from “Zhong Tao Hong Yu” (HY; round peach) and “Zhong Pan Tao #10” (ZPT10; normal flat peach). These samples were used to analyze candidate gene expression patterns. Samples from another two accessions, “Zao Huang Pao Tao” (ZH; normal flat peach) and “Xinjiang Pan Tao #2” (XJ2; aborted flat peach), were collected at 0, 7, 15, and 30 DAFB, to further validate the candidate genes. Total RNA was extracted from these fruit samples using an RNA-extracting kit (Hua Yue Yang, China). To check the relative expression levels of *PpOFP1* in transgenic tomato lines, RNA was extracted from young leaves using the same method. First- and second-strand complementary DNA (cDNA) samples were synthesized using a cDNA Synthesis System kit (TOYOBO, Japan) following the manufacturer’s protocol. The qRT-PCR was performed using a LightCycler 480 (Roche) with the following cycle conditions: 95 °C for 5 min, followed by 45 cycles at 95 °C for 10 s, 60 °C for 10 s and 72 °C for 20 s. The house-keeping gene RP-II (RNA polymerase II) was used as an internal control for peach [19]. The relative expression level was calculated using the 2^−ΔΔCt^ method [67].

### Overexpression of the candidate gene in tomato

The full-length coding region of *PpOFP1* was amplified from fruit cDNA sample of “XJ2” (aborting flat peach with homozygous genotype) by PCR using a high fidelity DNA polymerase (KOD-201, TOYOBO, Japan). The products were cloned into the *pBI121* vector downstream of the cauliflower mosaic virus (CaMV) 35S promoter using a one-step construction kit (C112, Vazyme, China). The constructs were then transformed into Micro-Tom tomato using *Agrobacterium tumefaciens* GV3101 following protocol described in Sun et al. [68]. After transformation, transgenic lines were obtained and their fruit shapes were analyzed. Primers used in this experiment are listed in Additional file 1: Table S13.

### Microscopic analysis of transgenic tomato fruit and flowers

Flower and fruit samples of transgenic tomato lines overexpressing *PpOFP1* were collected at 0 and 10 DAFB and fixed in FAA immediately for 2 days. The samples were then removed from FAA, rinsed thoroughly in deionized water, and then dehydrated using an ethanol gradient, cleared using xylene, and embedded in wax. The embedded samples were then sectioned (10 μm section thickness), and the sections were rehydrated and stained using aqueous toluidine blue (pH 7.0) [69]. Images were captured with a light microscope (Olympus) fitted with a camera (DP71, Olympus). To determine the cell number along the vertical axis, the cells were counted one by one manually under a light microscope.

### Dual-luciferase reporter assay

To determine whether the deletion in the promoter region of *PpMYB10.1* affects the promoter activity, dual-luciferase reporter assays were carried out. Different promoter regions of *PpMYB10.1* were cloned into *pGreen-II-0800-LUC* vector using the one-step construction kit as described above (C112, Vazyme, China). The promoters of *PpMYB10.1* with or without the deletion were cloned into *pGreen-II-0800-LUC* to check the influence on gene expression. The constructs were transformed into GV3101 and transient expression assays were performed using tobacco leaves. The relative luciferase activities were detected and photos taken by a Tanon-5200Multi machine (Biotanon, China).

## Supplementary information

**Additional file 1.** Supplementary tables S1-S13.

**Additional file 2.** Supplementary figures S1-S38.

**Additional file 3.** Review history.

## Data Availability

The sequence data have been deposited in NCBI Sequence Read Archive (SRA) under accession PRJNA630113 [70]. The SNPs and SVs in Variant Call Format (VCF) have been deposited in Zenodo [71]. The scripts used in this study were available in GitHub [72].
