# Supplementary material for: An integrated peach genome structural variation map uncovers genes associated with fruit traits
Source: Genome Biol. 2020 Oct 6;21:258. doi: 10.1186/s13059-020-02169-y (PMC7539501; doi:10.1186/s13059-020-02169-y)
Supplement: Supplementary file 2 — Additional file 2. Supplementary figures S1-S38. [file 13059_2020_2169_MOESM2_ESM.docx]

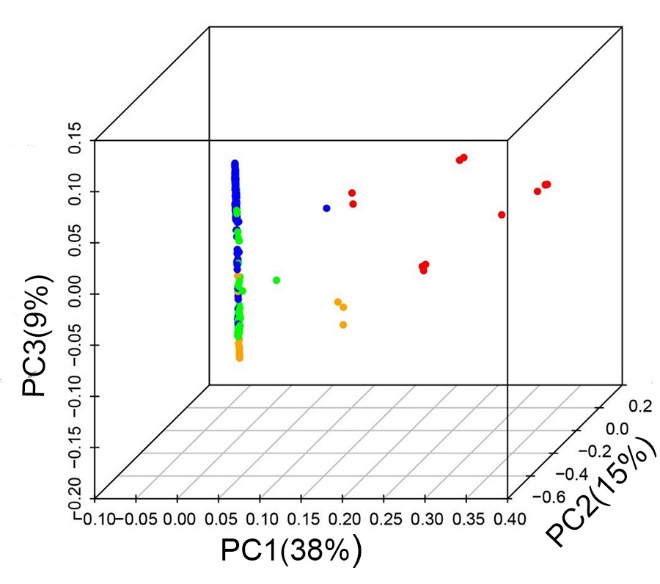


**Fig S1. PCA of 336 peach accession.**


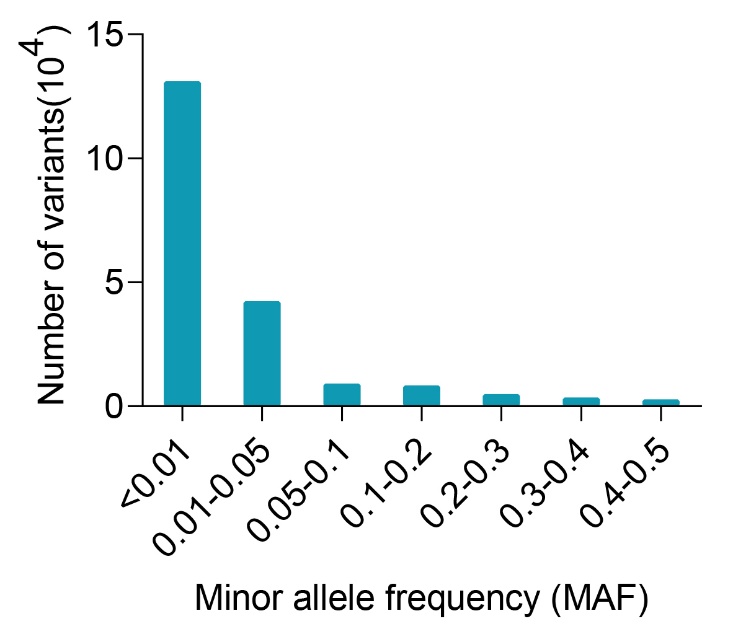


**Fig S2. Distribution of SVs based on the site-frequency spectrum.**


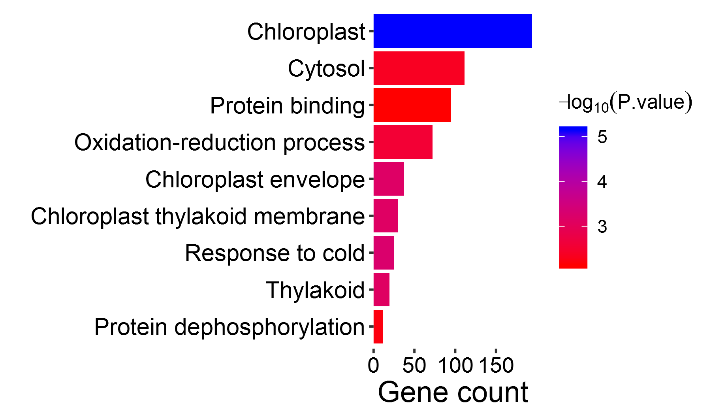


**Fig S3. GO enrichment of the 512 genes that are not affected by SVs in the 336 peach accessions.**


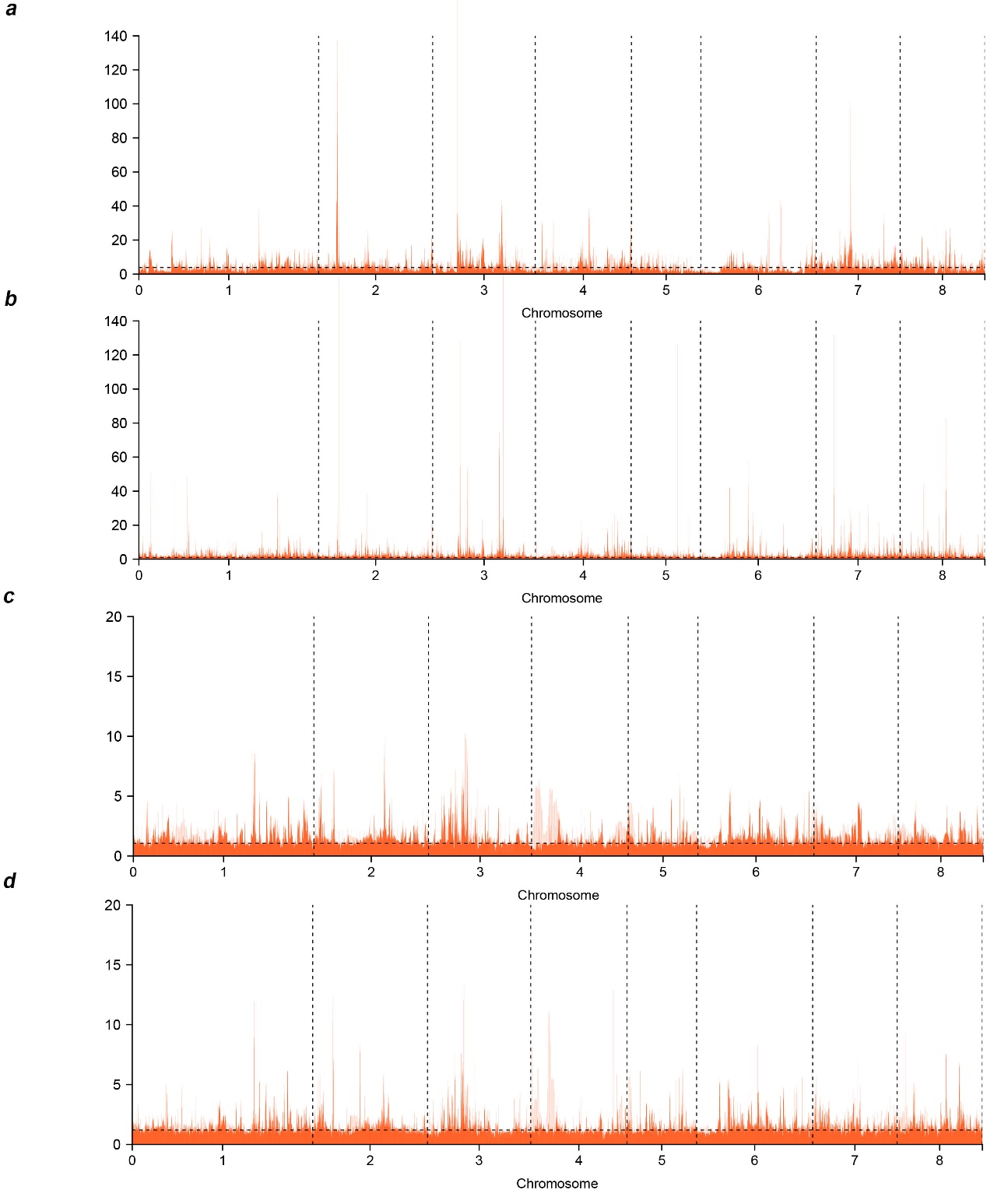


π_landrace_/π_improved cultivar_

π_landrace_/π_improved cultivar_

π_wild_/π_landrace_

π_wild_/π_landrace_

**Fig S4. Genome-wide detection of selective sweeps during domestication and improvement.** **a** Selective sweeps during domestication identified using SNPs. **b** Selective sweeps during domestication identified using SVs. **c** Selective sweeps during improvement using identified SNPs. **d** Selective sweeps during improvement identified using SVs.


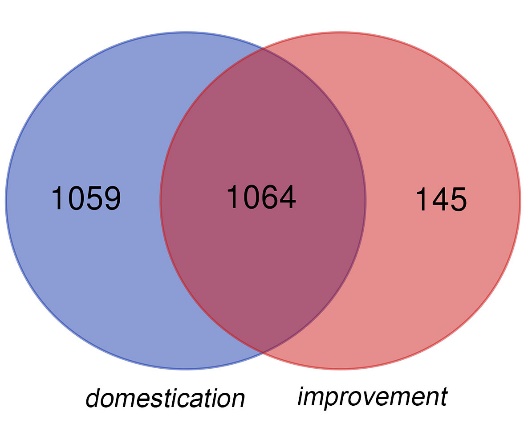


**Fig S5. Venn diagram showing genes selected during peach domestication and improvement.**

**
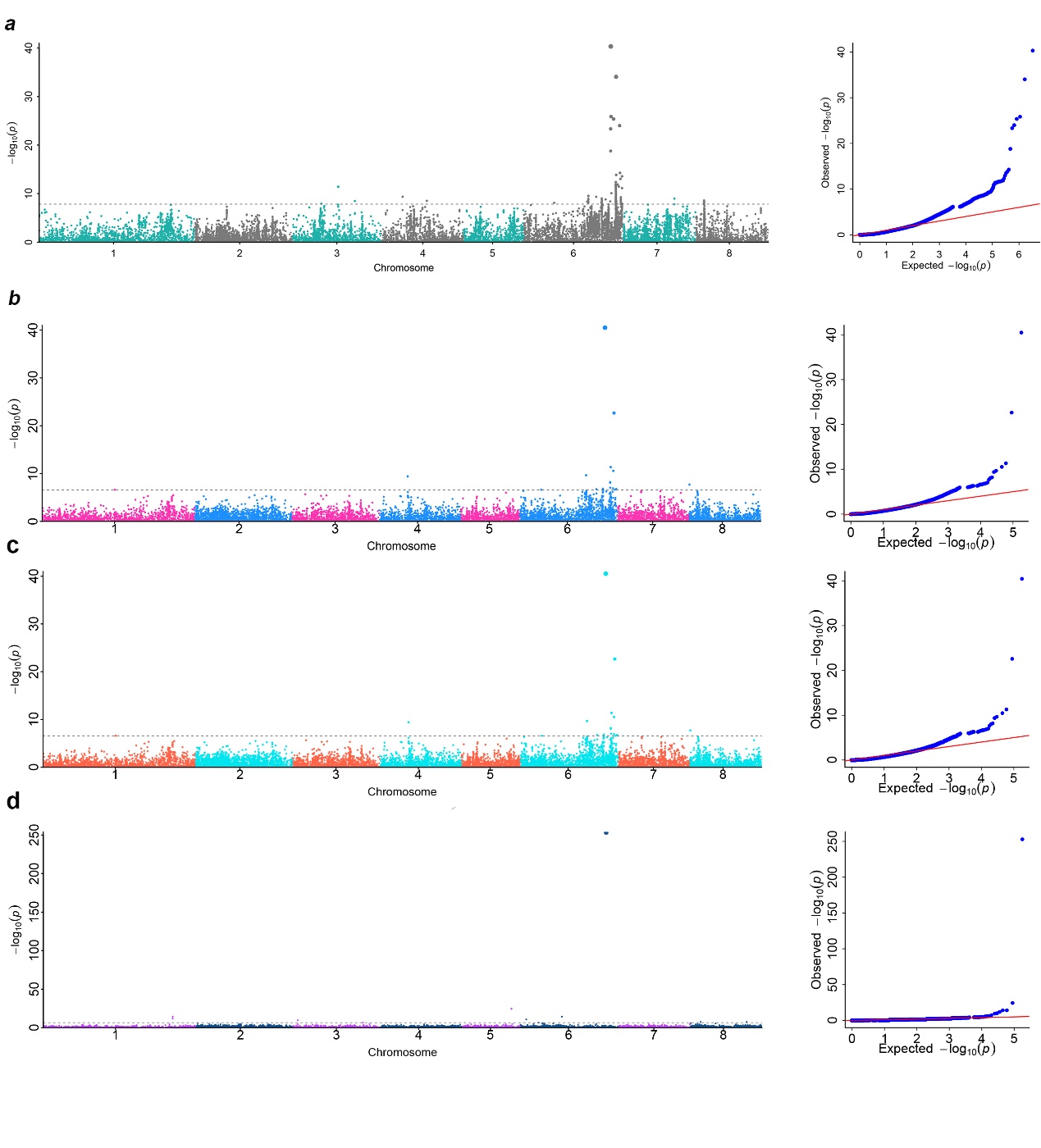
**

**Fig S6. Manhattan and QQ plots of genome-wide association studies (GWAS) of fruit shape (flat/round). a** GWAS using SNPs with MLM model. **b** GWAS using SVs with MLM model. **c** GWAS using SVs with CMLM model. **d** GWAS using SVs with FarmCPU model.


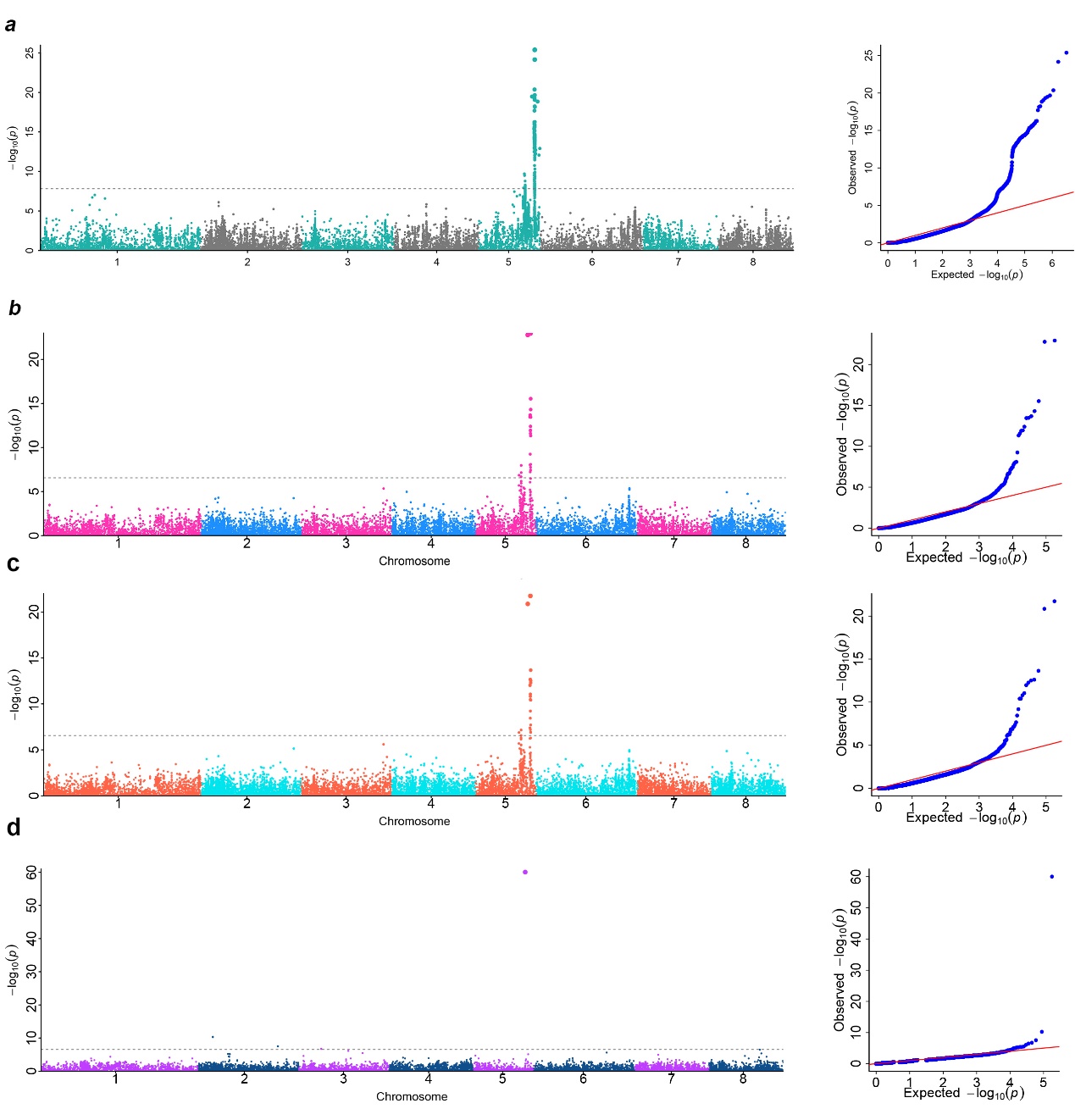


**Fig S7. Manhattan and QQ plots of genome-wide association studies (GWAS) of fruit hairiness (peach/nectarine). a** GWAS using SNPs with MLM model. **b** GWAS using SVs with MLM model. **c** GWAS using SVs with CMLM model. **d** GWAS using SVs with FarmCPU model.


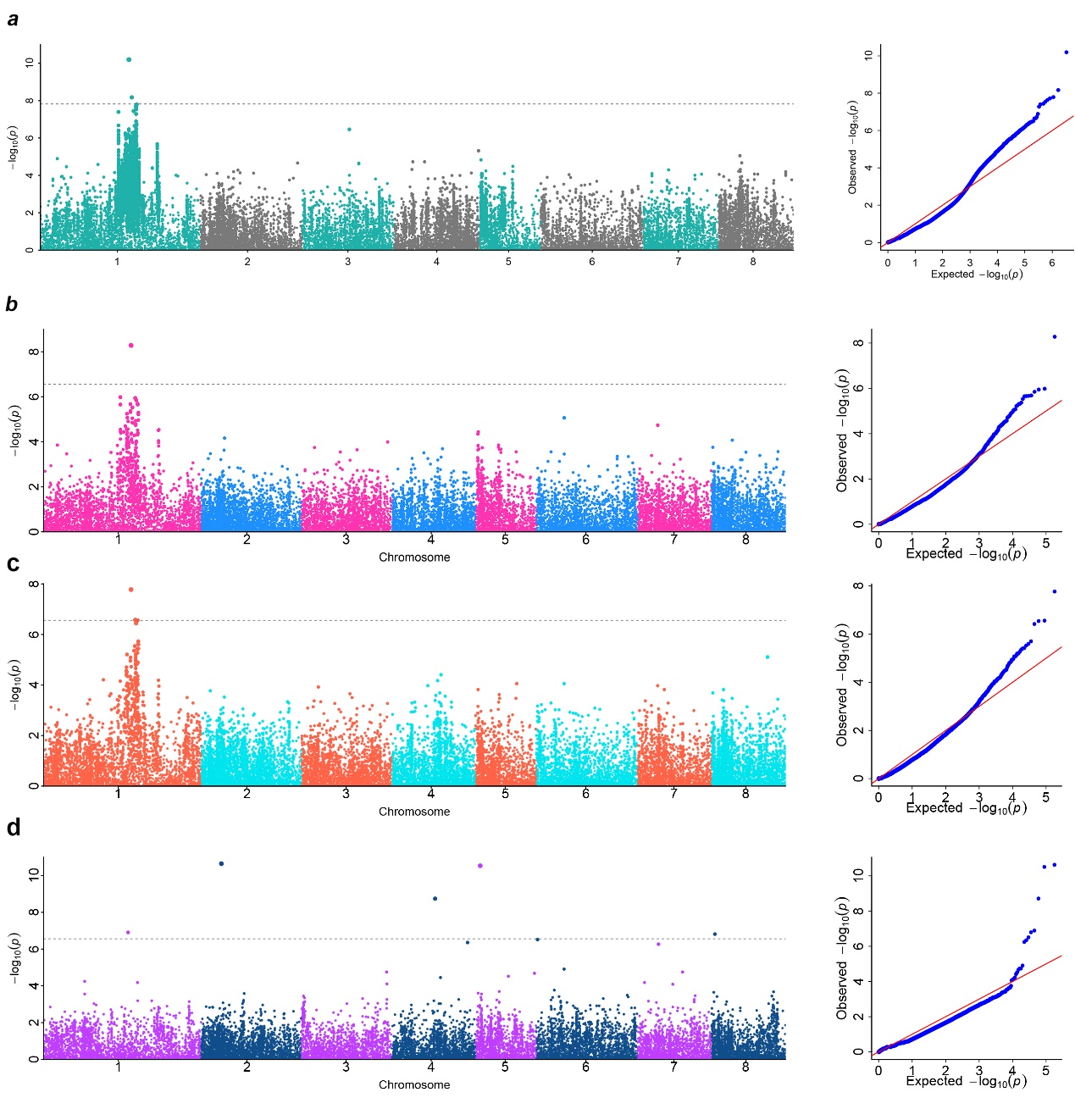


**Fig S8. Manhattan and QQ plots of genome-wide association studies (GWAS) of fruit flesh color (white/yellow). a** GWAS using SNPs with MLM model. **b** GWAS using SVs with MLM model. **c** GWAS using SVs with CMLM model. **d** GWAS using SVs with FarmCPU model.


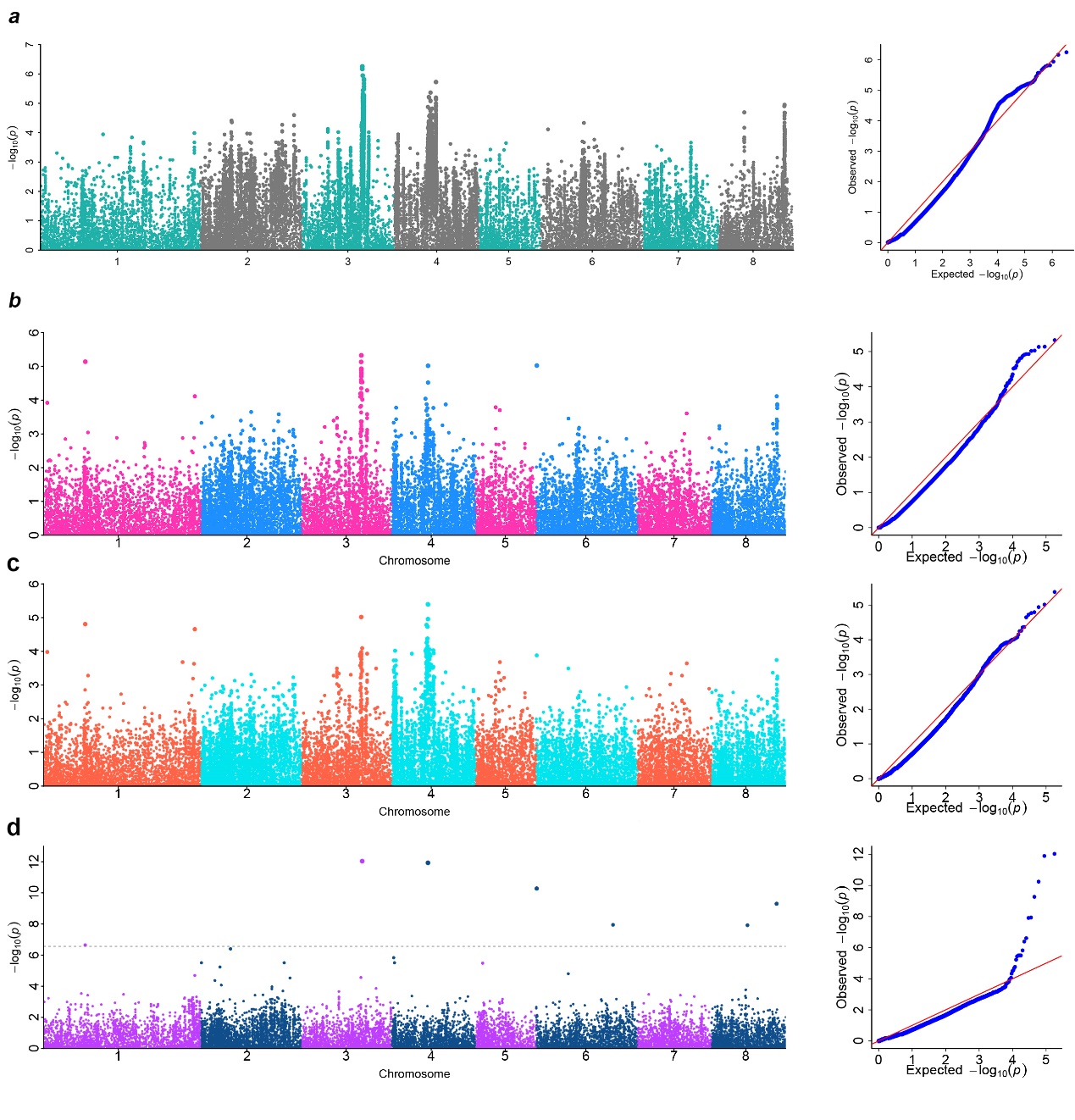


**Fig S9. Manhattan and QQ plots of genome-wide association studies (GWAS) of fruit** **flesh color around the stone (with/without anthocyanin). a** GWAS using SNPs with MLM model. **b** GWAS using SVs with MLM model. **c** GWAS using SVs with CMLM model. **d** GWAS using SVs with FarmCPU model.


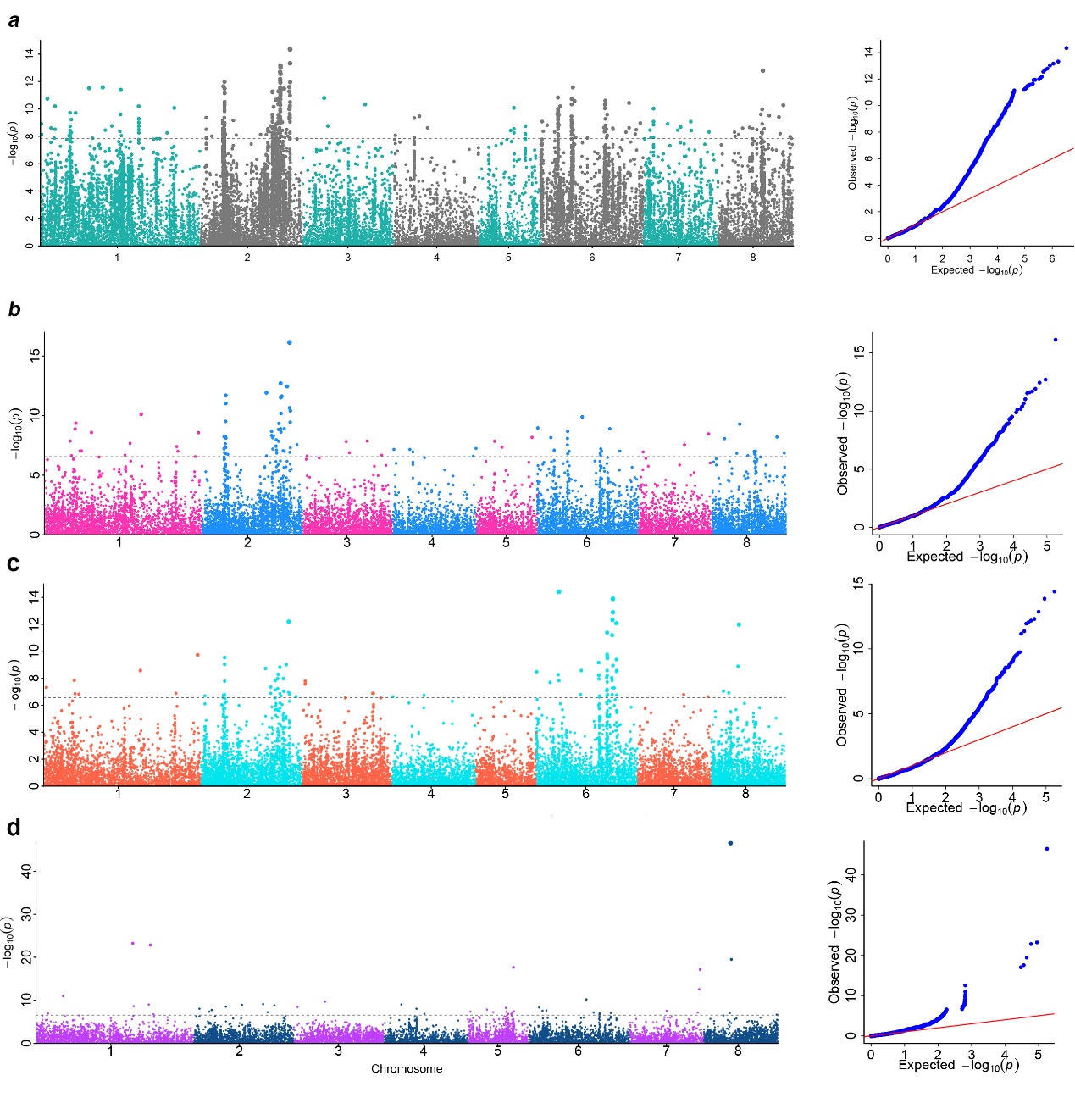


**Fig S10. Manhattan and QQ plots of genome-wide association studies (GWAS) of flower shape (double/single). a** GWAS using SNPs with MLM model. **b** GWAS using SVs with MLM model. **c** GWAS using SVs with CMLM model. **d** GWAS using SVs with FarmCPU model.


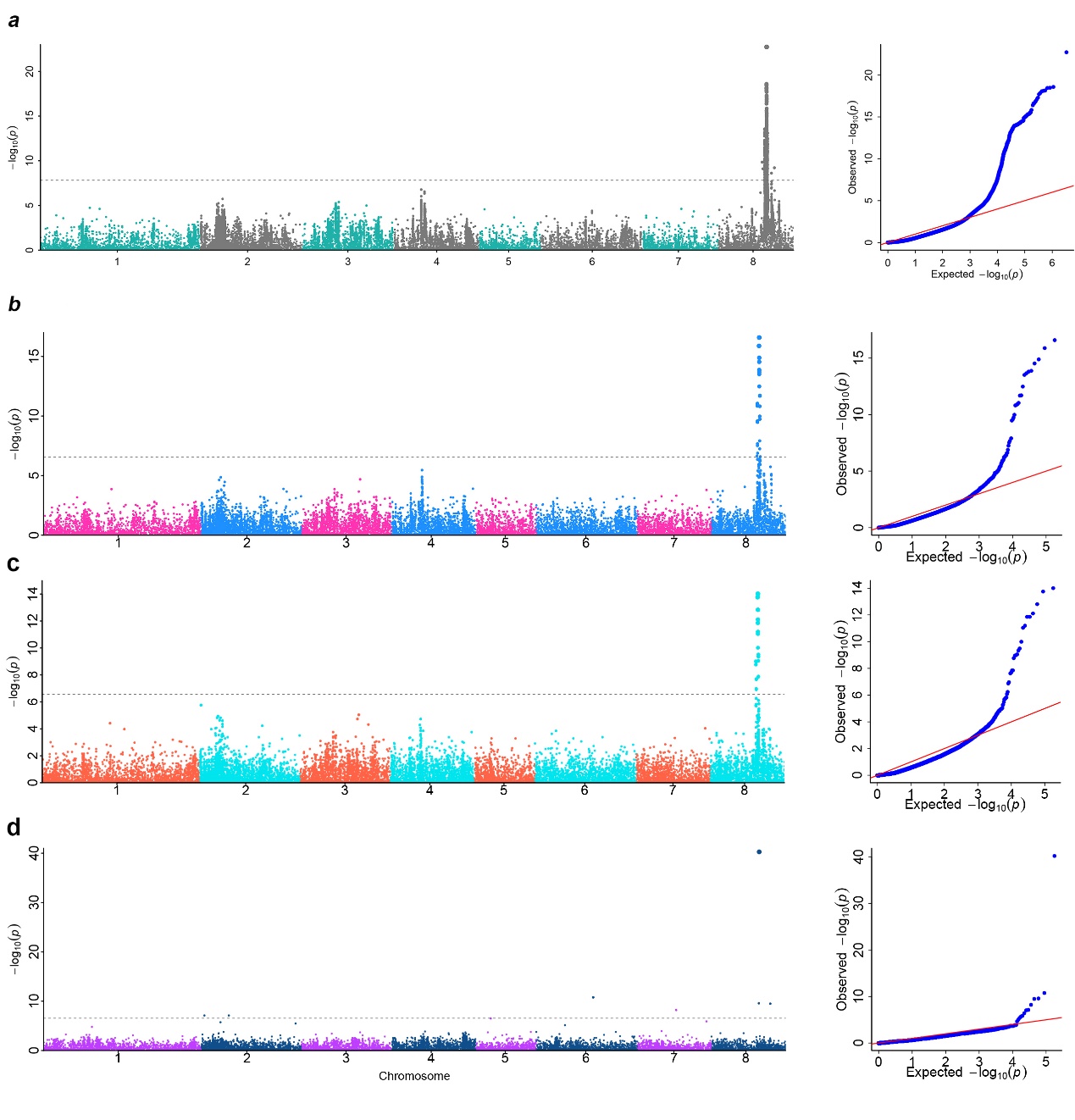


**Fig S11. Manhattan plot and QQ plots of genome-wide association studies (GWAS) of flower shape (showy/non-showy). a** GWAS using SNPs with MLM model. **b** GWAS using SVs with MLM model. **c** GWAS using SVs with CMLM model. **d** GWAS using SVs with FarmCPU model.


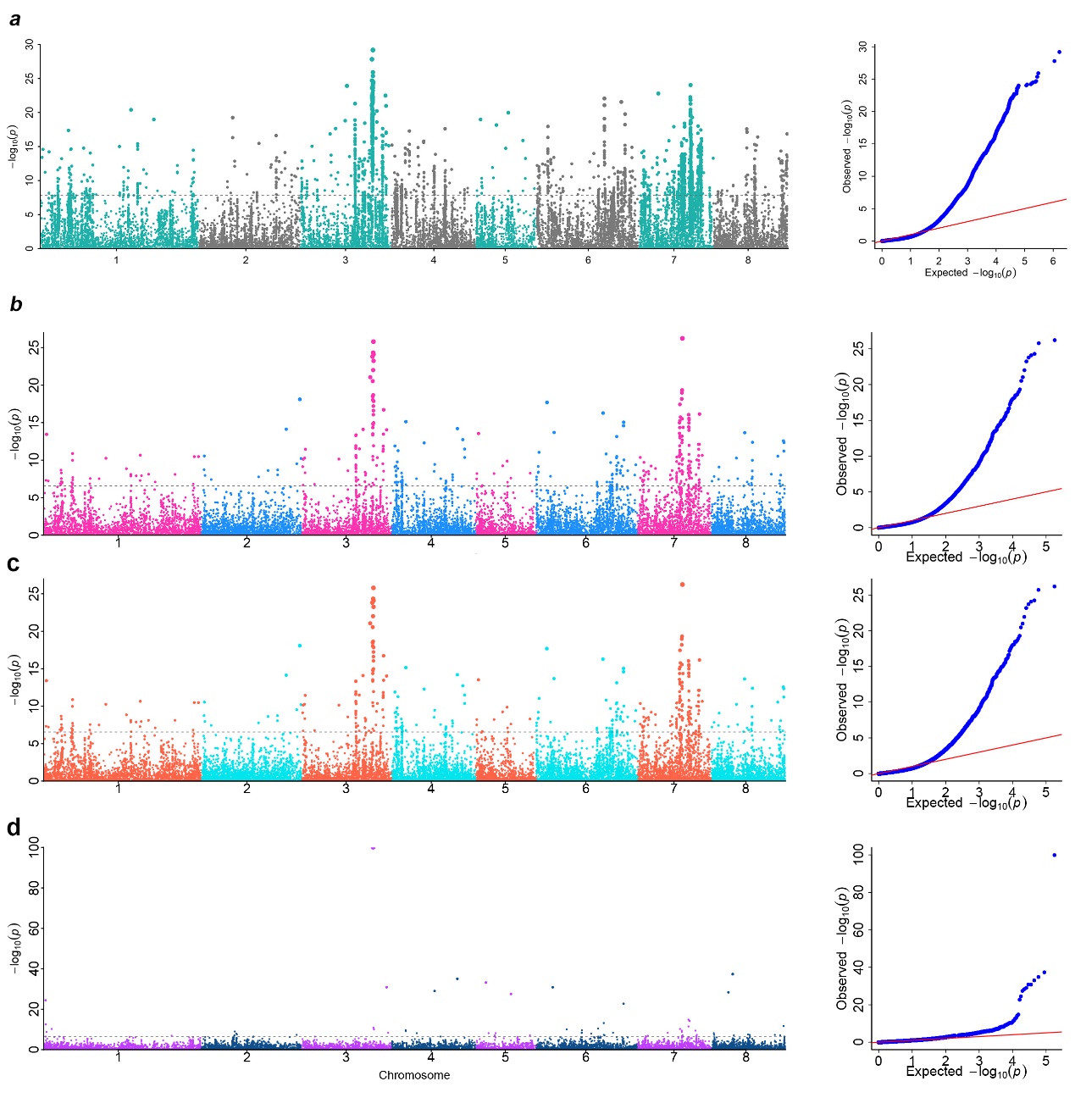


**Fig S12. Manhattan and QQ plots of genome-wide association studies (GWAS) of weeping branch (weeping/normal). a** GWAS using SNPs with MLM model. **b** GWAS using SVs with MLM model. **c** GWAS using SVs with CMLM model. **d** GWAS using SVs with FarmCPU model.


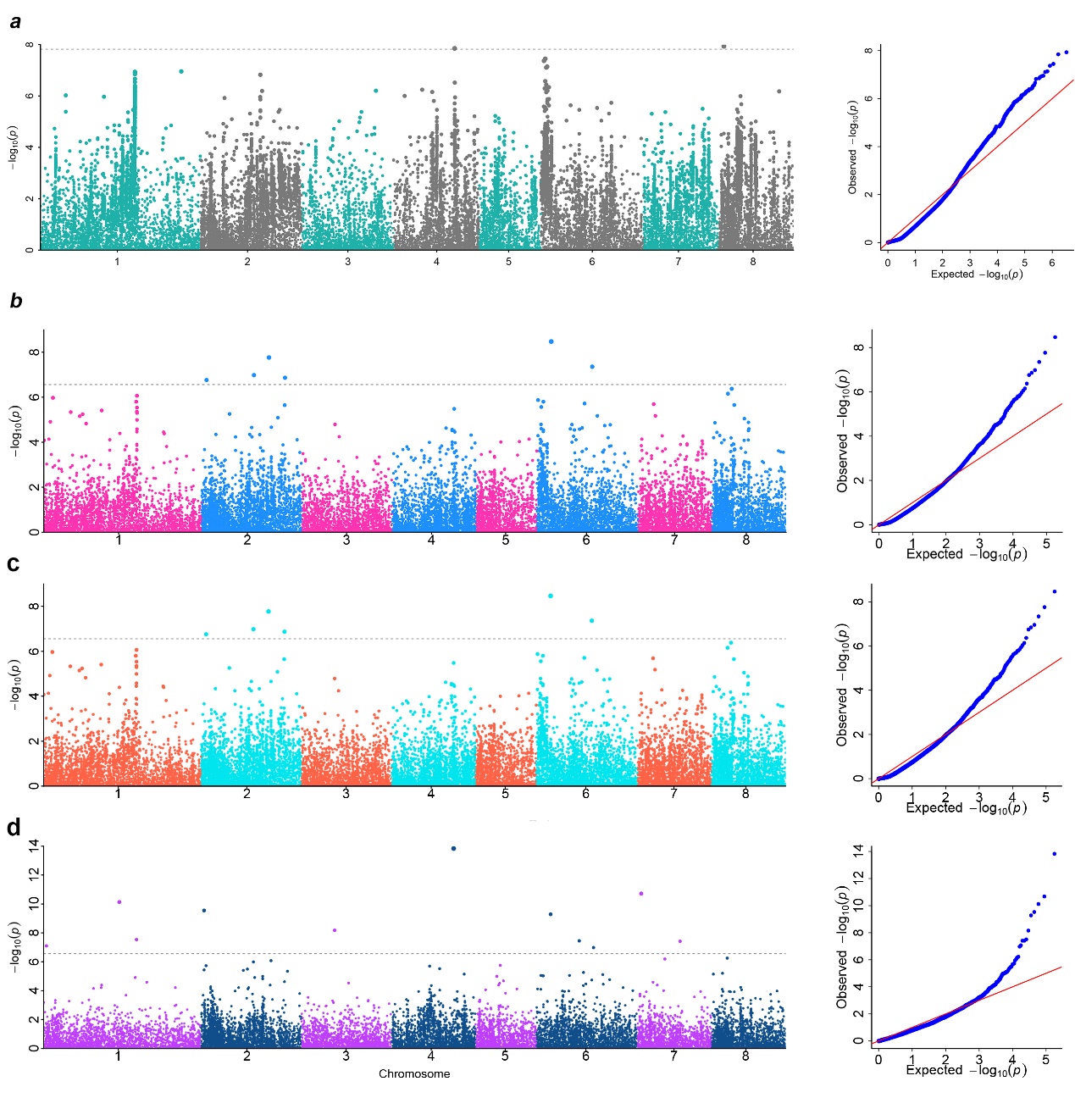


**Fig S13. Manhattan and QQ plots of genome-wide association studies (GWAS) of pollen fertility. a** GWAS using SNPs with MLM model. **b** GWAS using SVs with MLM model. **c** GWAS using SVs with CMLM model. **d** GWAS using SVs with FarmCPU model.


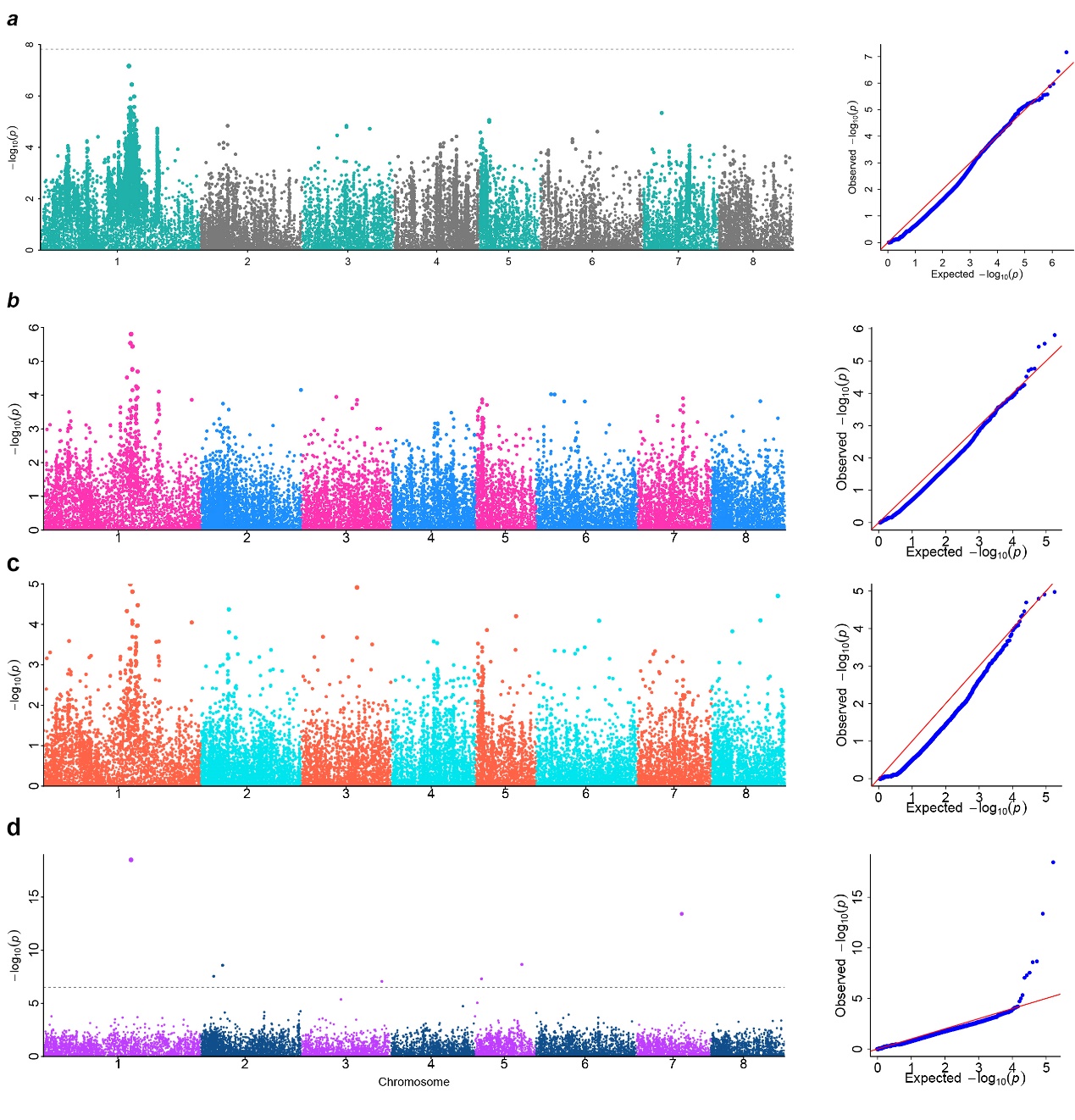


**Fig S14. Manhattan and QQ plots of genome-wide association studies (GWAS) of hypanthium inner wall color (white/yellow). a** GWAS using SNPs with MLM model. **b** GWAS using SVs with MLM model. **c** GWAS using SVs with CMLM model. **d** GWAS using SVs with FarmCPU model.


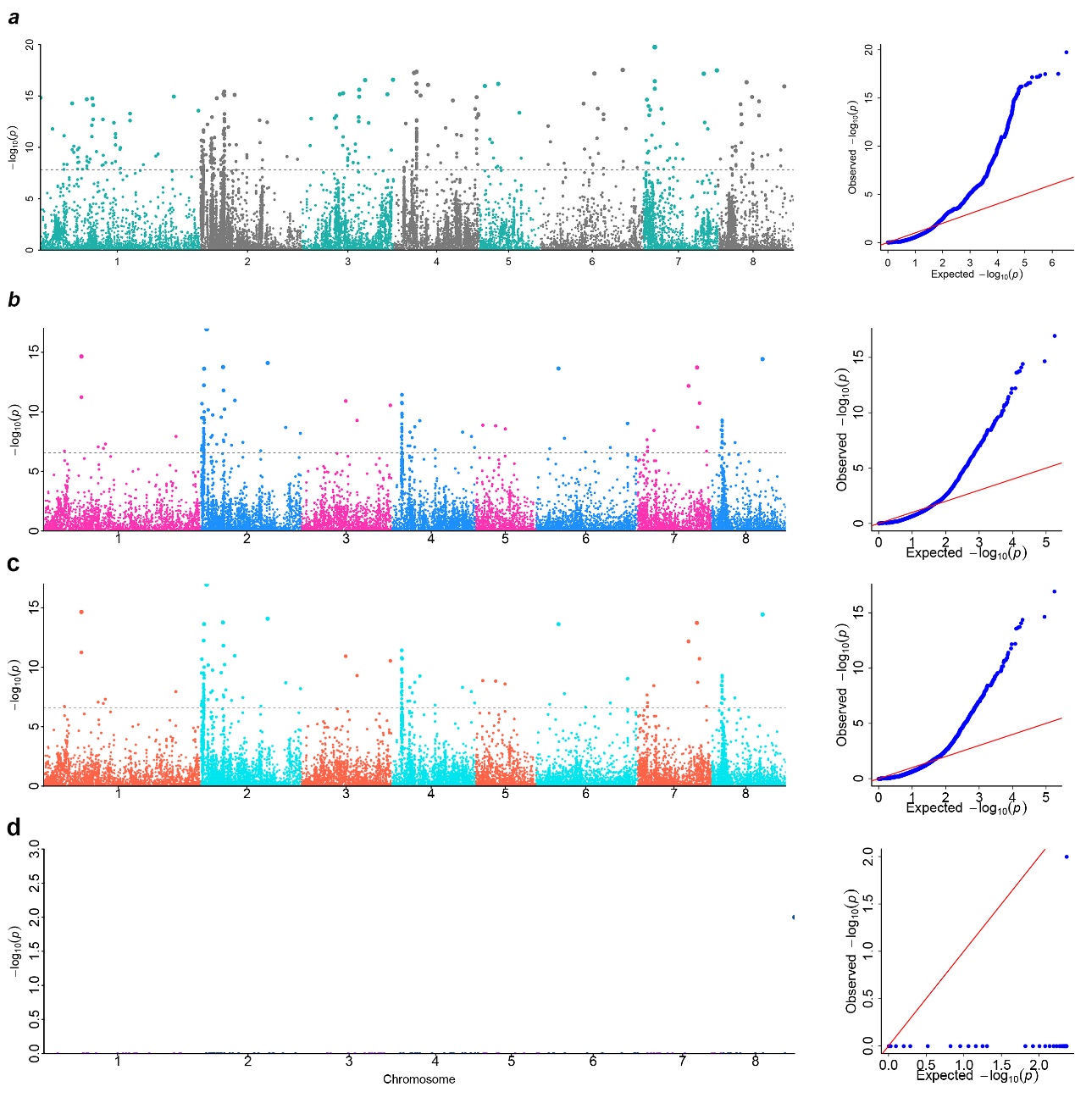


**Fig S15. Manhattan and QQ plots of genome-wide association studies (GWAS) of kernel taste (sweet/bitter). a** GWAS using SNPs with MLM model. **b** GWAS using SVs with MLM model. **c** GWAS using SVs with CMLM model. **d** GWAS using SVs with FarmCPU model.


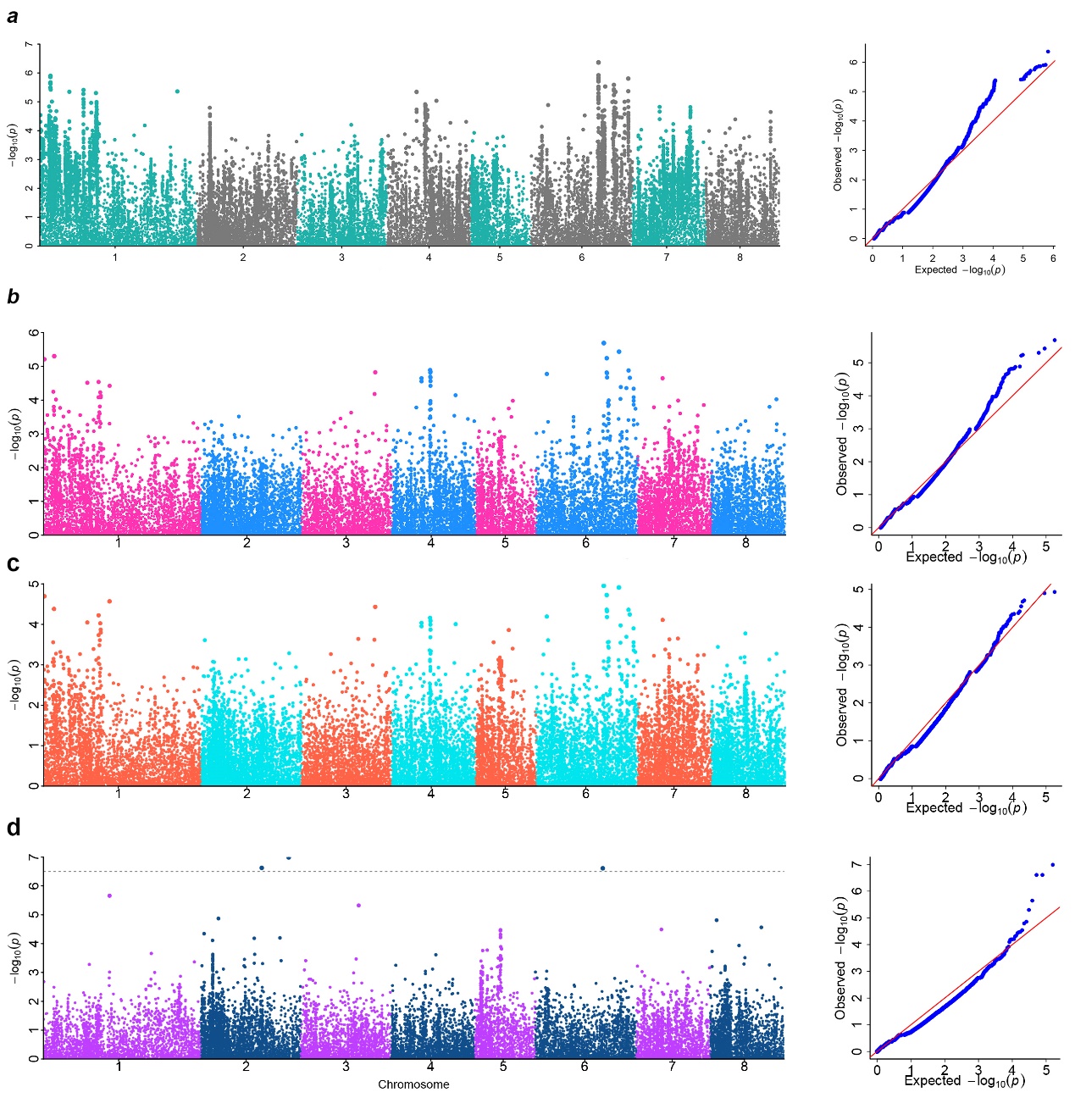


**Fig S16. Manhattan pand QQ plots of genome-wide association studies (GWAS) of internode length. a** GWAS using SNPs with MLM model. **b** GWAS using SVs with MLM model. **c** GWAS using SVs with CMLM model. **d** GWAS using SVs with FarmCPU model.


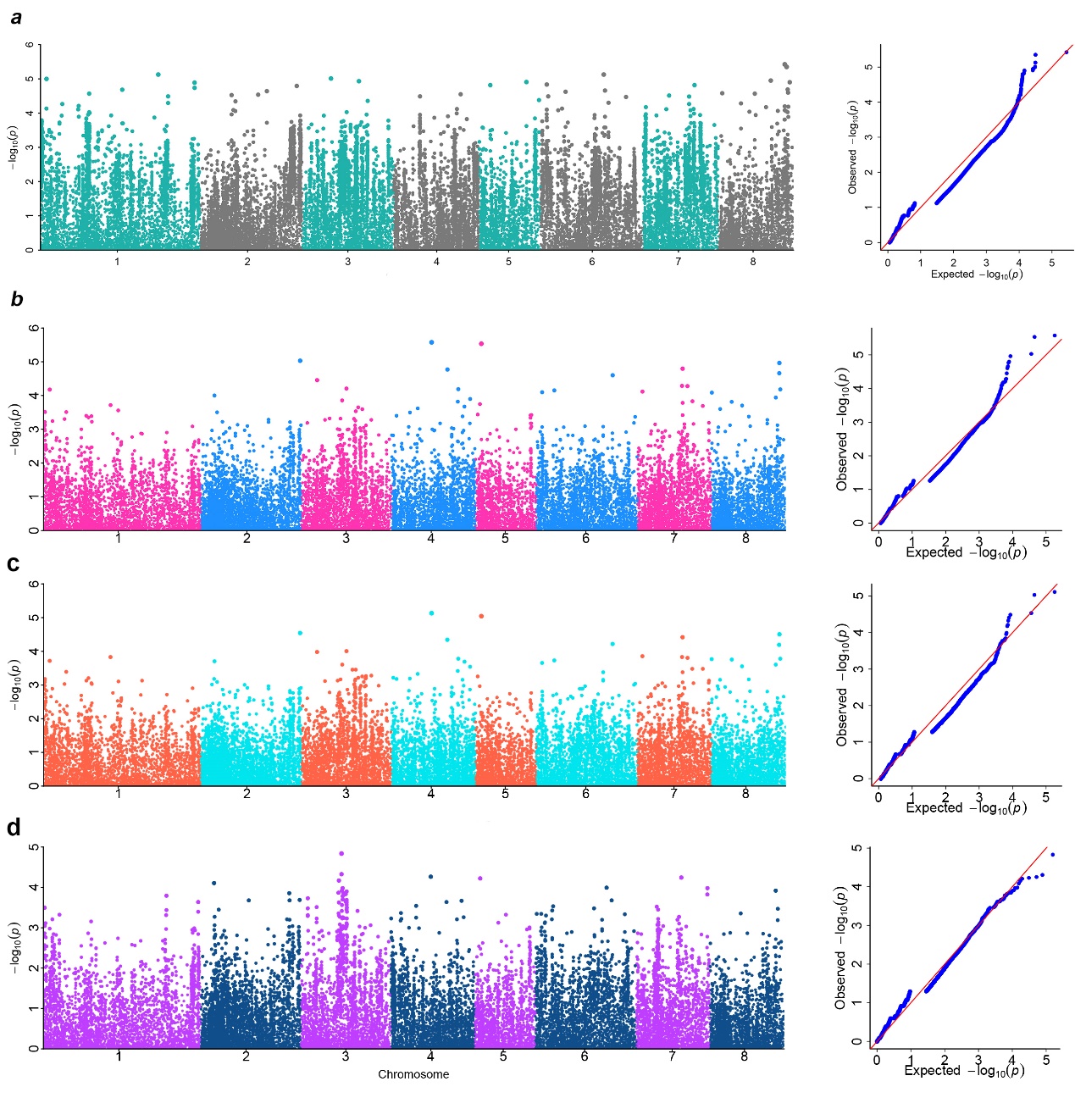


**Fig S17. Manhattan and QQ plots of genome-wide association studies (GWAS) of flower/leaf bud ratio. a** GWAS using SNPs with MLM model. **b** GWAS using SVs with MLM model. **c** GWAS using SVs with CMLM model. **d** GWAS using SVs with FarmCPU model.


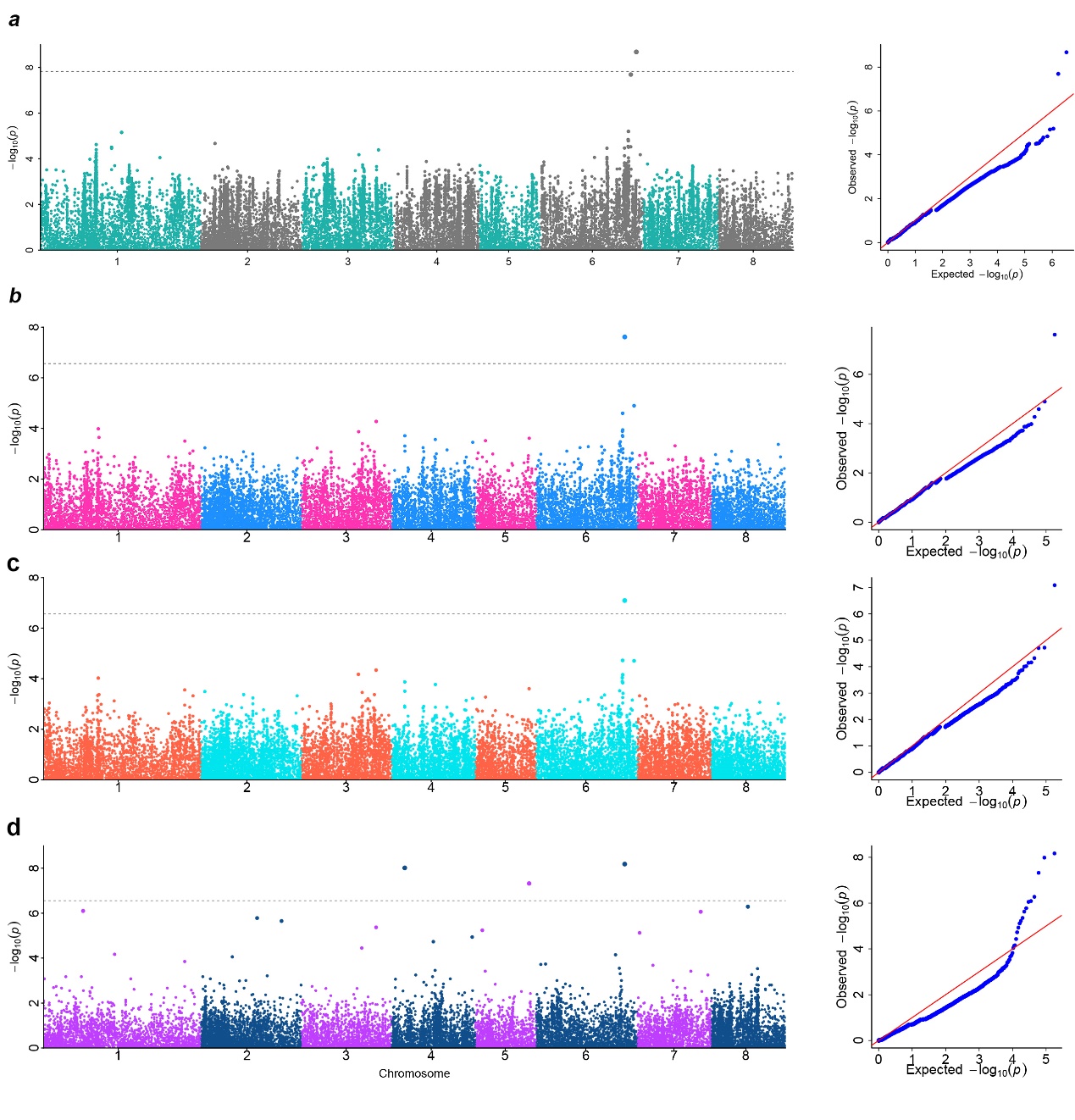


**Fig S18. Manhattan and QQ plots of genome-wide association studies (GWAS) of relative height between pistil and stigma. a** GWAS using SNPs with MLM model. **b** GWAS using SVs with MLM model. **c** GWAS using SVs with CMLM model. **d** GWAS using SVs with FarmCPU model.


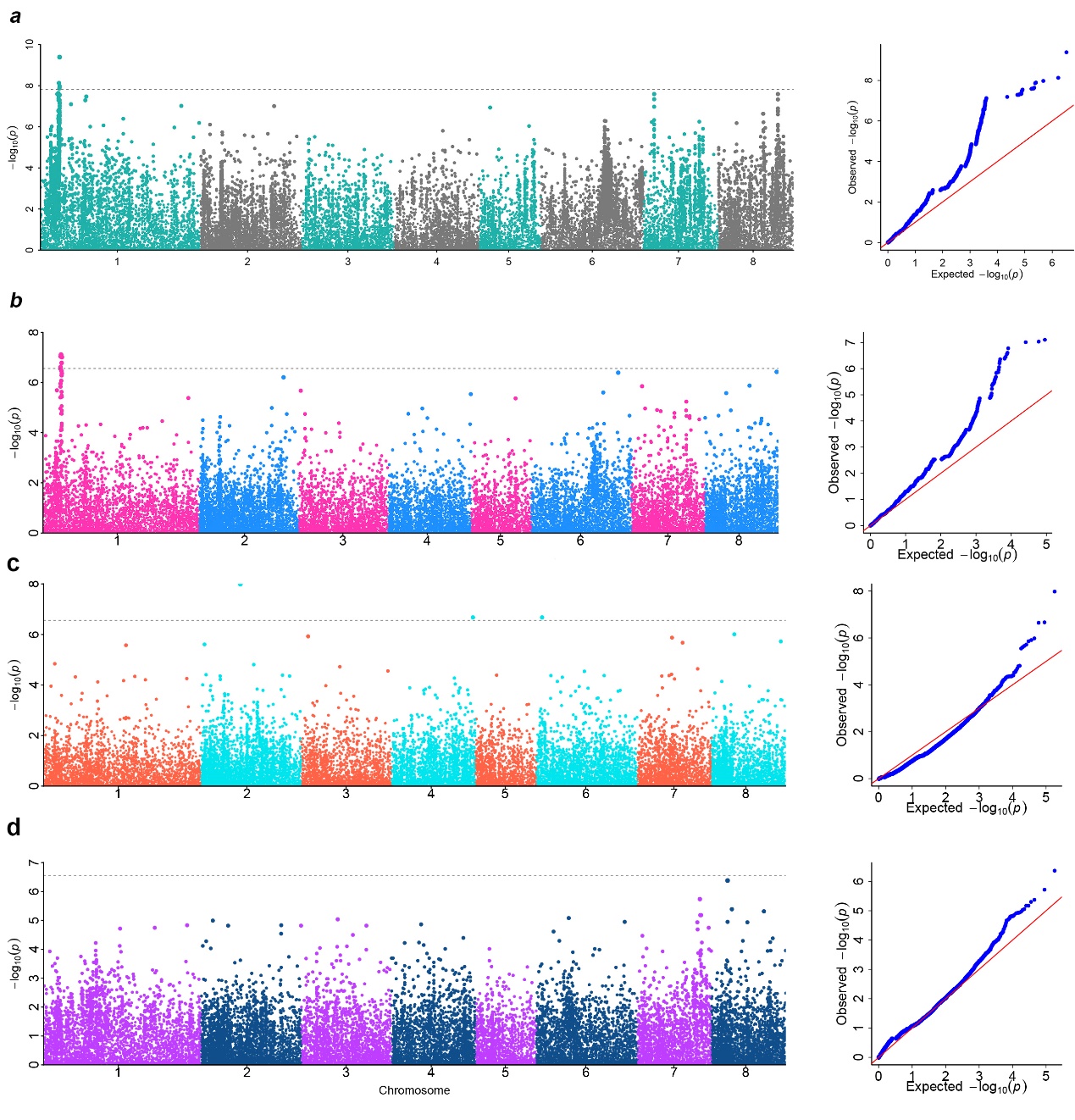


**Fig S19. Manhattan and QQ plots of genome-wide association studies (GWAS) of anther color. a** GWAS using SNPs with MLM model. **b** GWAS using SVs with MLM model. **c** GWAS using SVs with CMLM model. **d** GWAS using SVs with FarmCPU model.


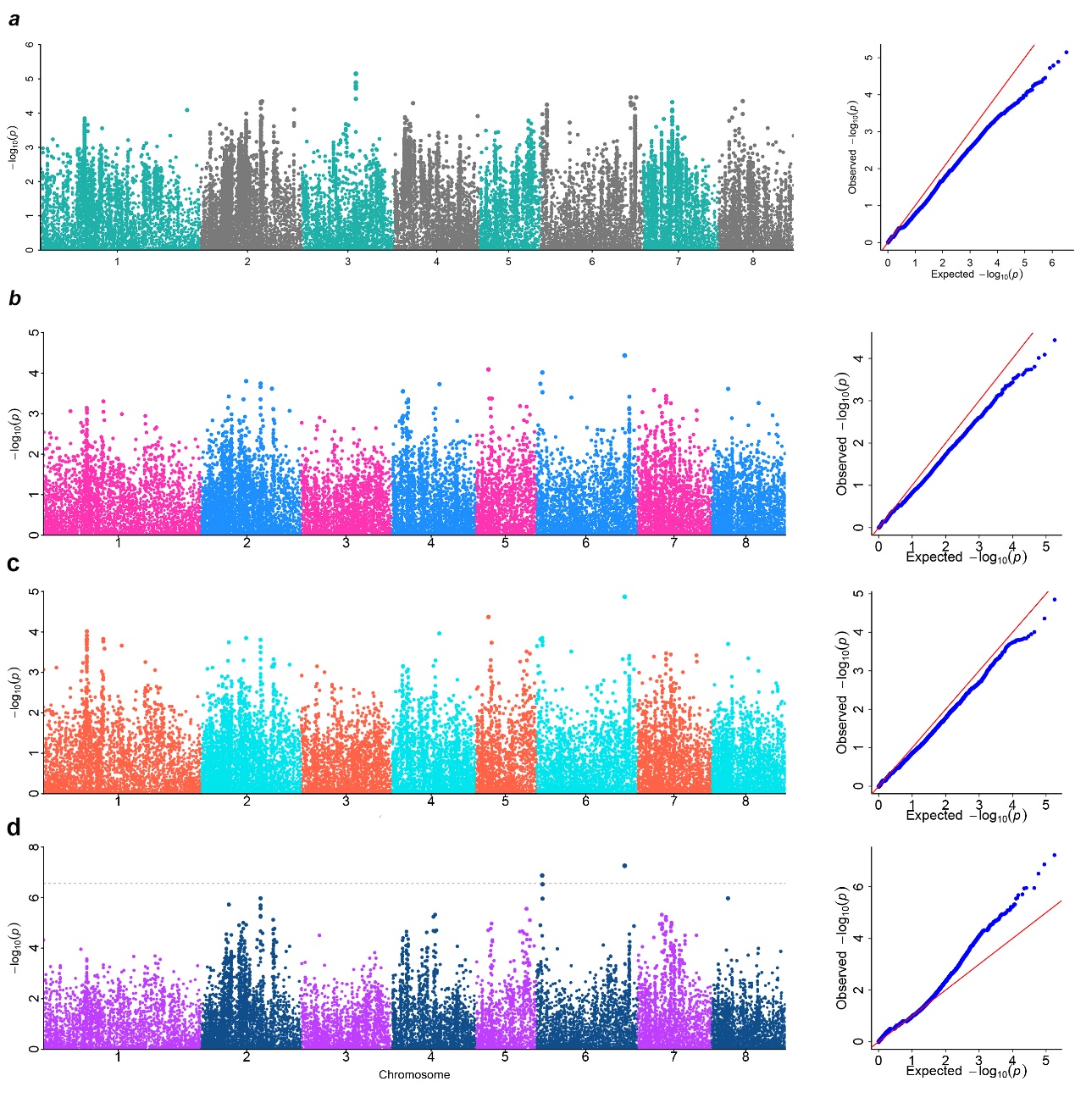


**Fig S20. Manhattan and QQ plots of genome-wide association studies (GWAS) of suture depth. a** GWAS using SNPs with MLM model. **b** GWAS using SVs with MLM model. **c** GWAS using SVs with CMLM model. **d** GWAS using SVs with FarmCPU model.


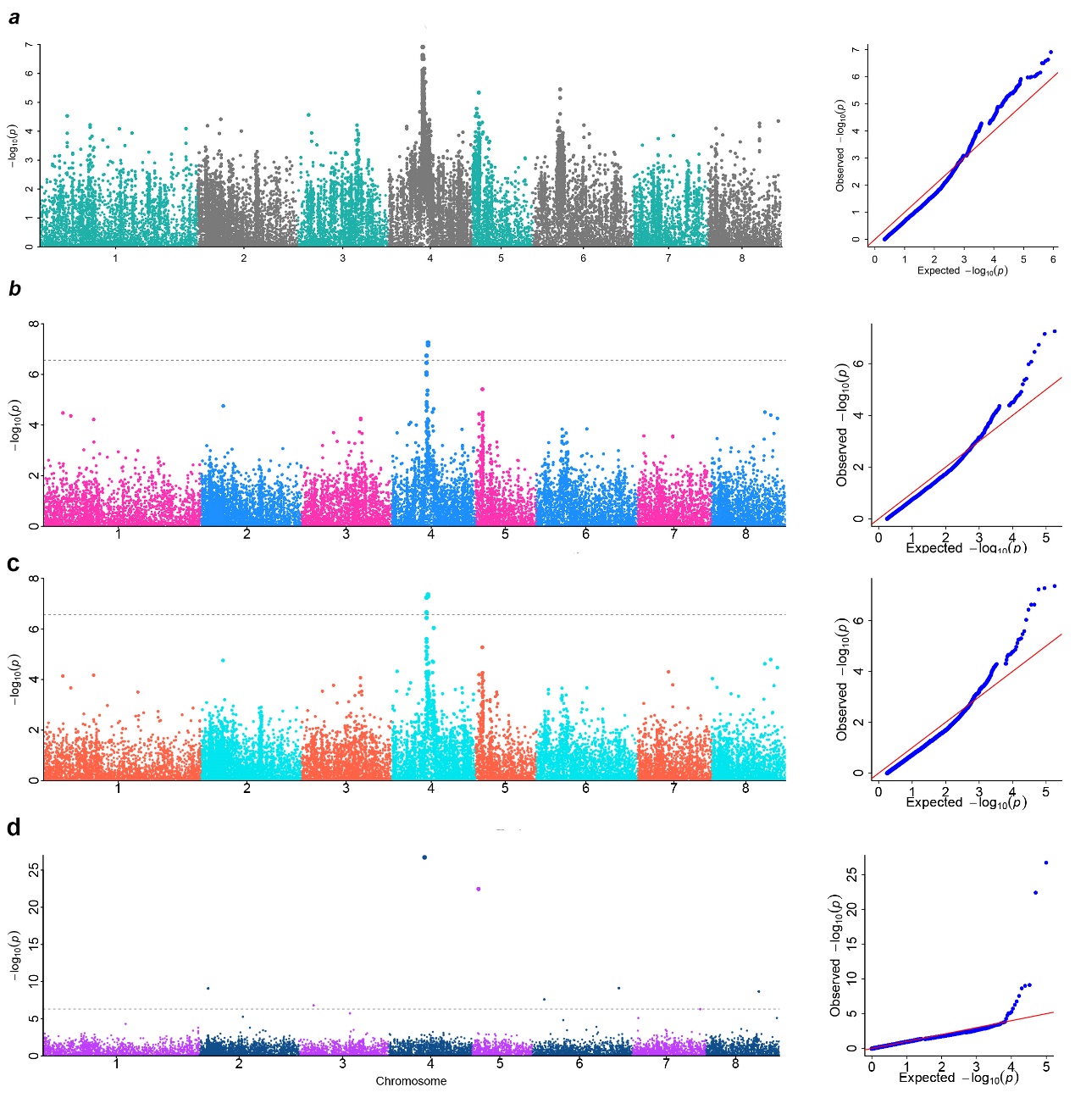

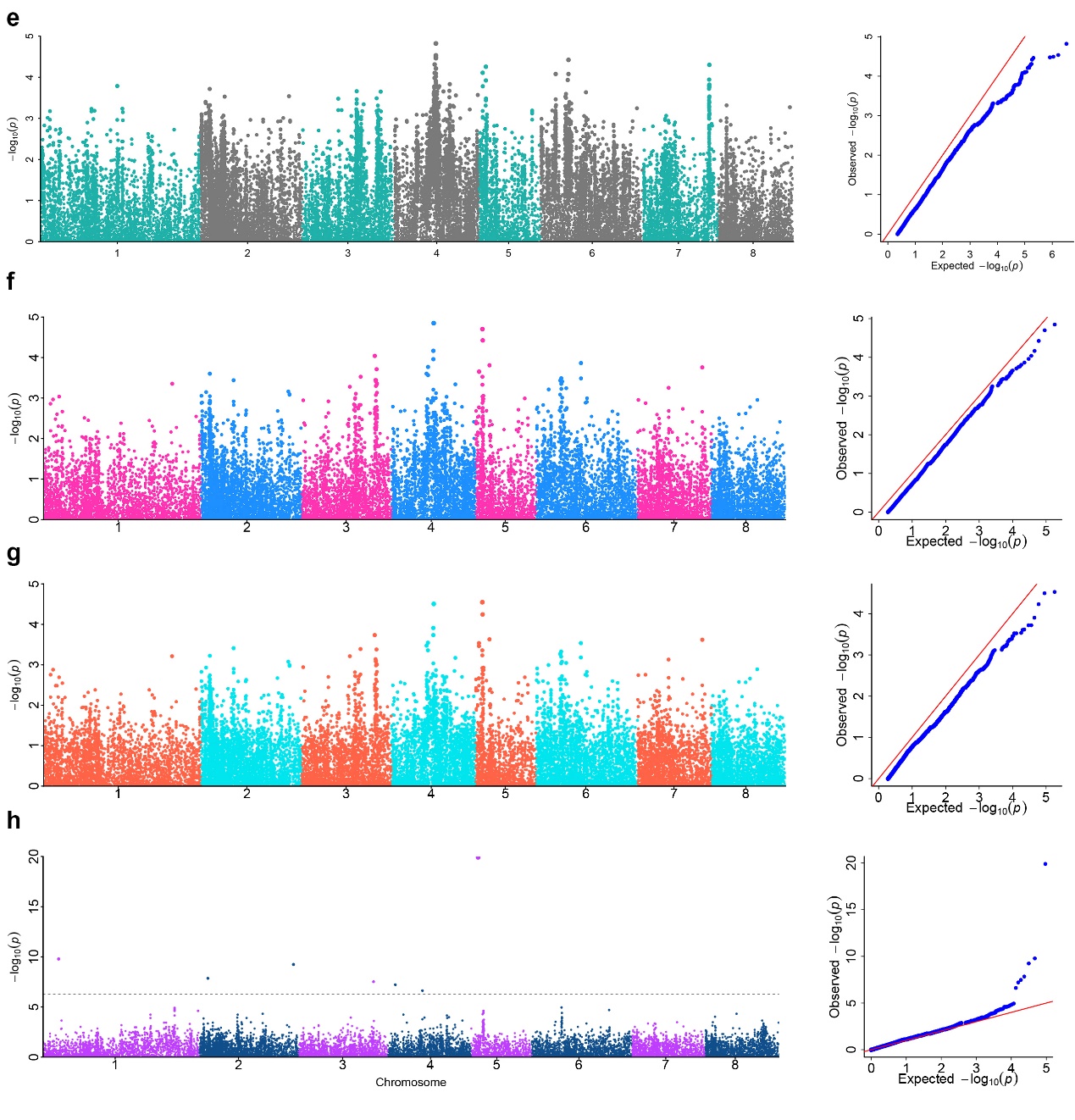


**Fig S21. Manhattan and QQ plots of genome-wide association studies (GWAS) of fruit development period. a** GWAS using SNPs with MLM model in 2008. **b** GWAS using SVs with MLM model in 2008. **c** GWAS using SVs with CMLM model in 2008. **d** GWAS using SVs with FarmCPU model in 2008. **e** GWAS using SNPs with MLM model in 2010. **f** GWAS using SVs with MLM model in 2010. **g** GWAS using SVs with CMLM model in 2010. **h** GWAS using SVs with FarmCPU model in 2010.


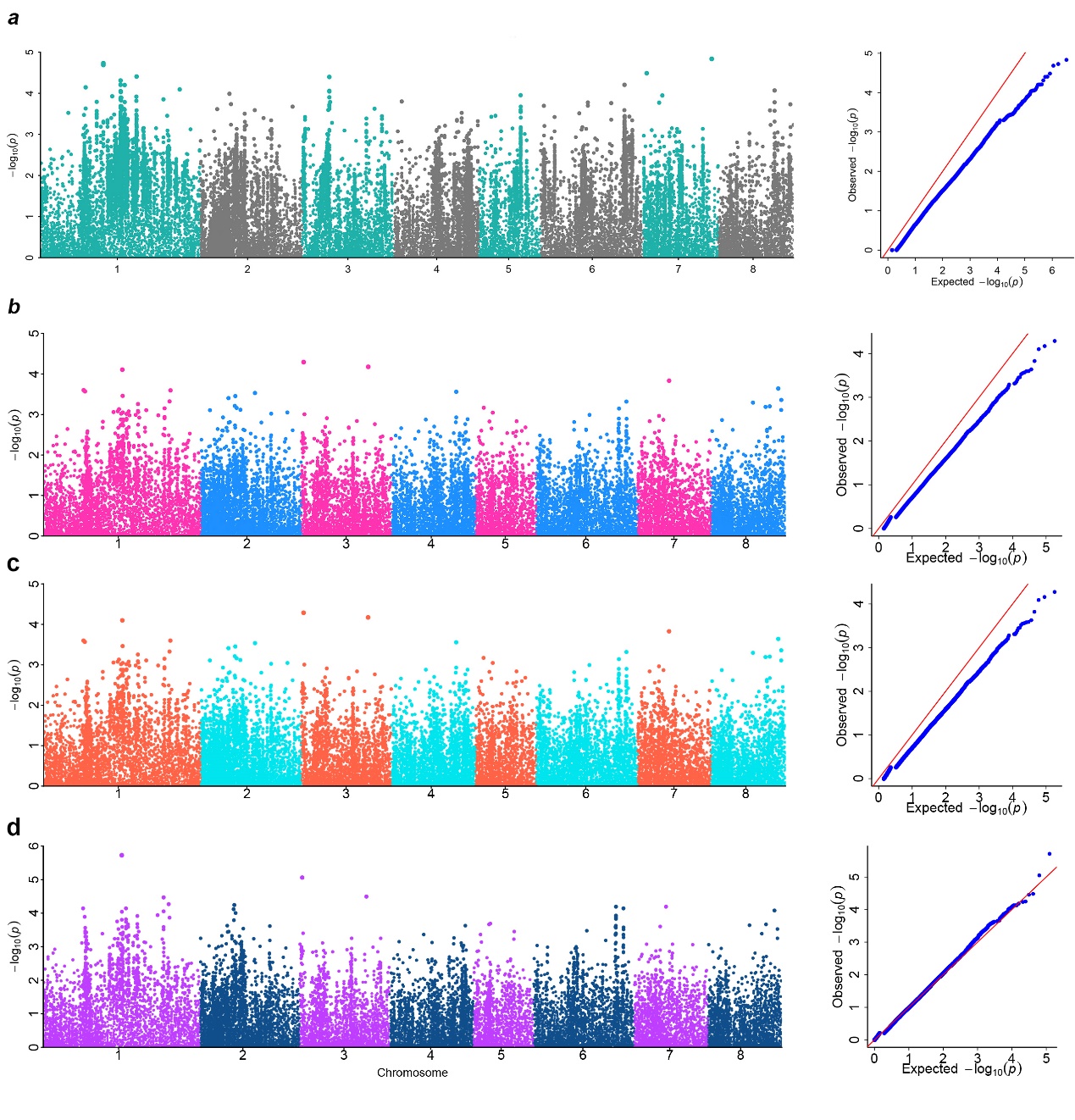

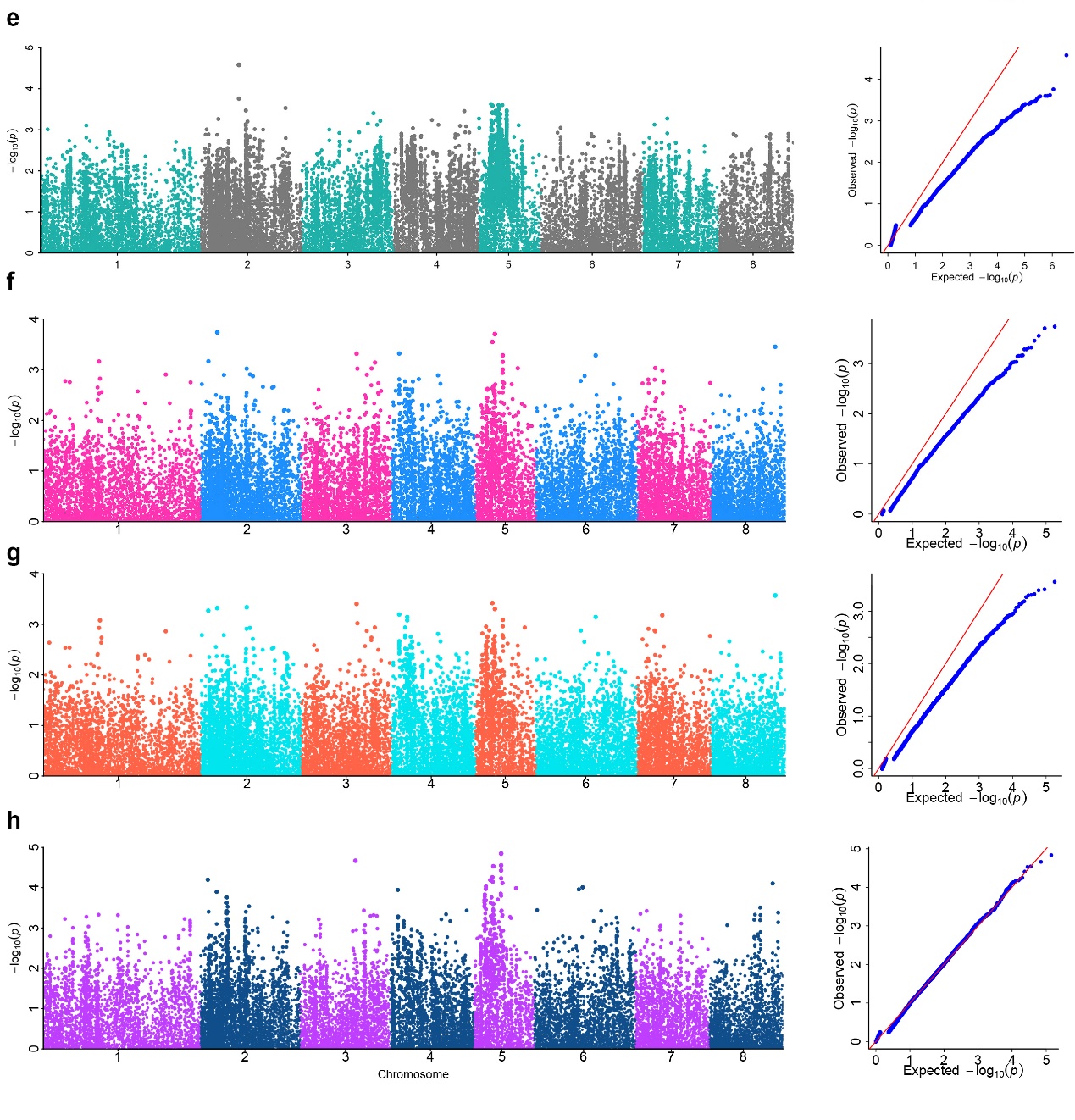


**Fig S22. Manhattan and QQ plots of genome-wide association studies (GWAS) of development period. a** GWAS using SNPs with MLM model in 2008. **b** GWAS using SVs with MLM model in 2008. **c** GWAS using SVs with CMLM model in 2008. **d** GWAS using SVs with FarmCPU model in 2008. **e** GWAS using SNPs with MLM model in 2010. **f** GWAS using SVs with MLM model in 2010. **g** GWAS using SVs with CMLM model in 2010. **h** GWAS using SVs with FarmCPU model in 2010.


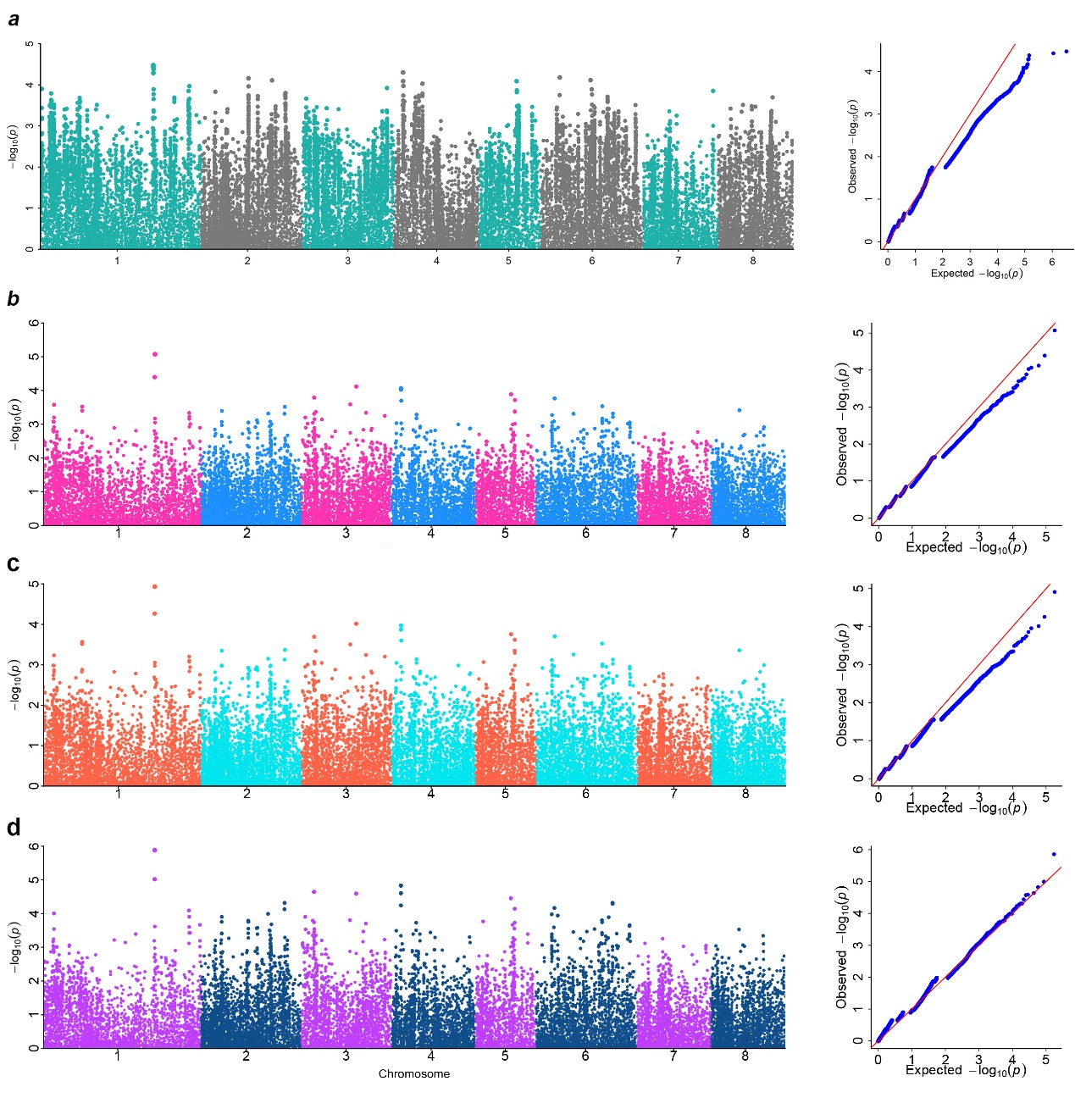


**Fig S23. Manhattan and QQ plots of genome-wide association studies (GWAS) of leaf length. a** GWAS using SNPs with MLM model. **b** GWAS using SVs with MLM model. **c** GWAS using SVs with CMLM model. **d** GWAS using SVs with FarmCPU model.


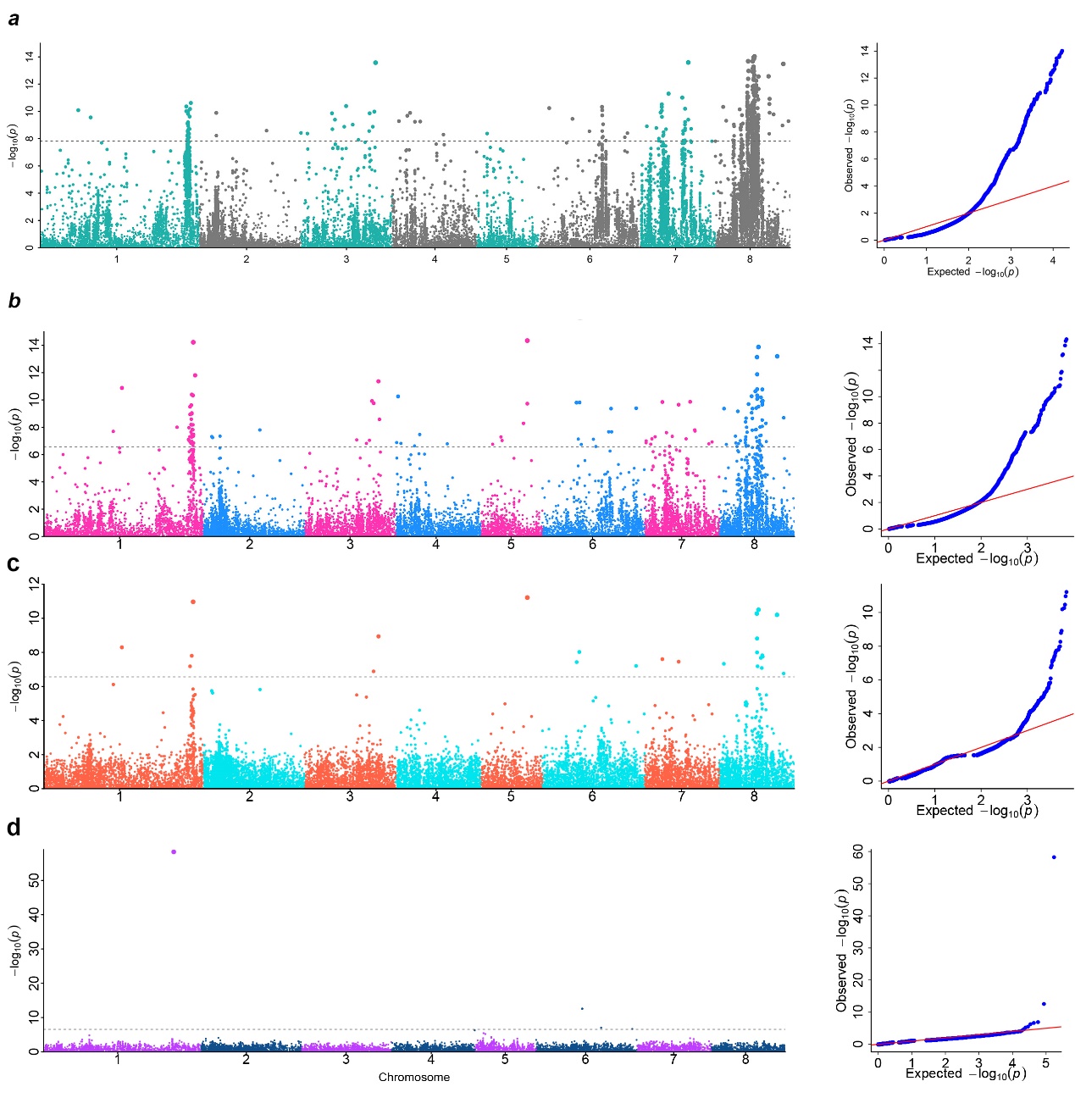


**Fig S24. Manhattan and QQ plots of genome-wide association studies (GWAS) of leaf width. a** GWAS using SNPs with MLM model. **b** GWAS using SVs with MLM model. **c** GWAS using SVs with CMLM model. **d** GWAS using SVs with FarmCPU model.


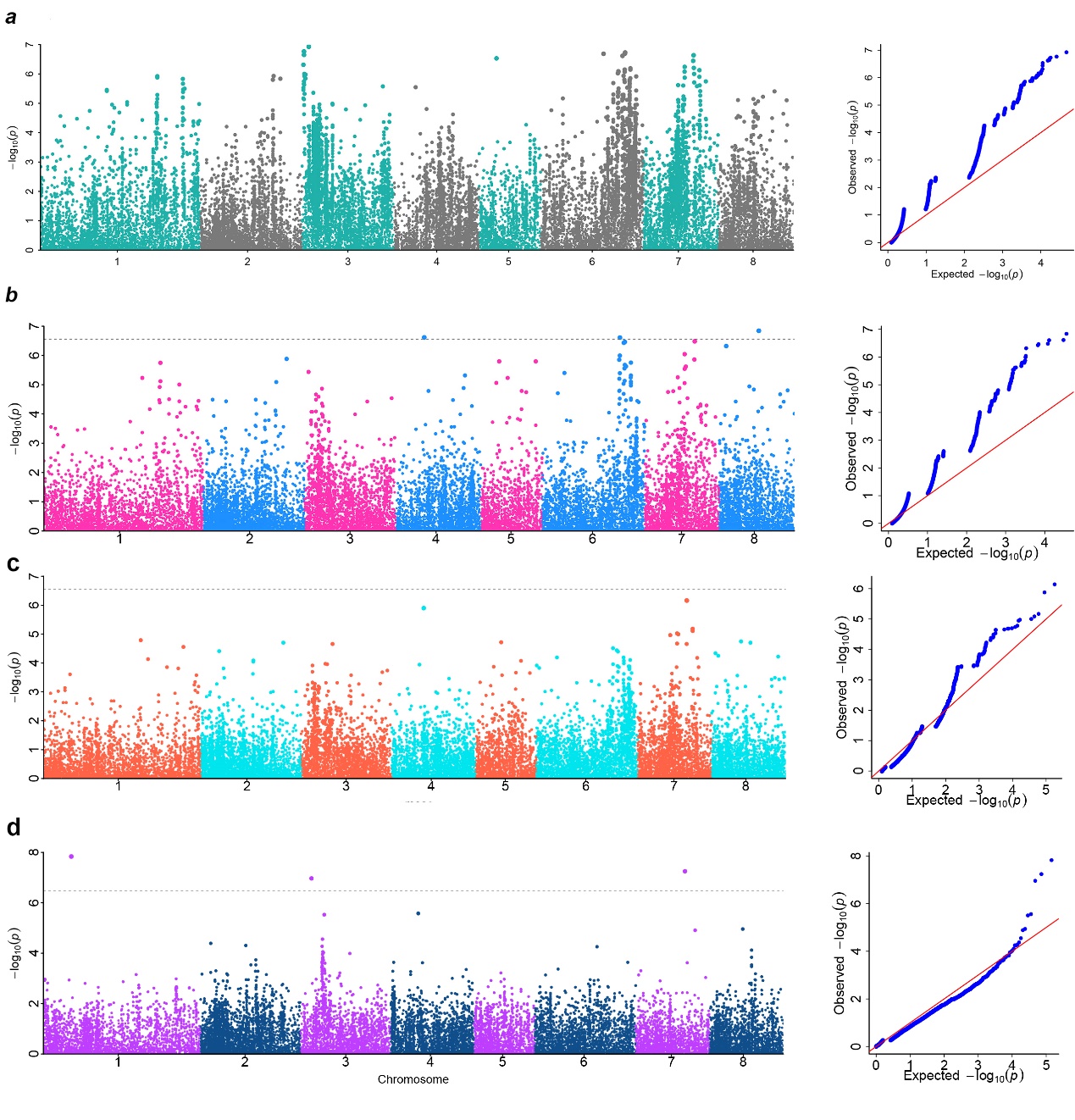

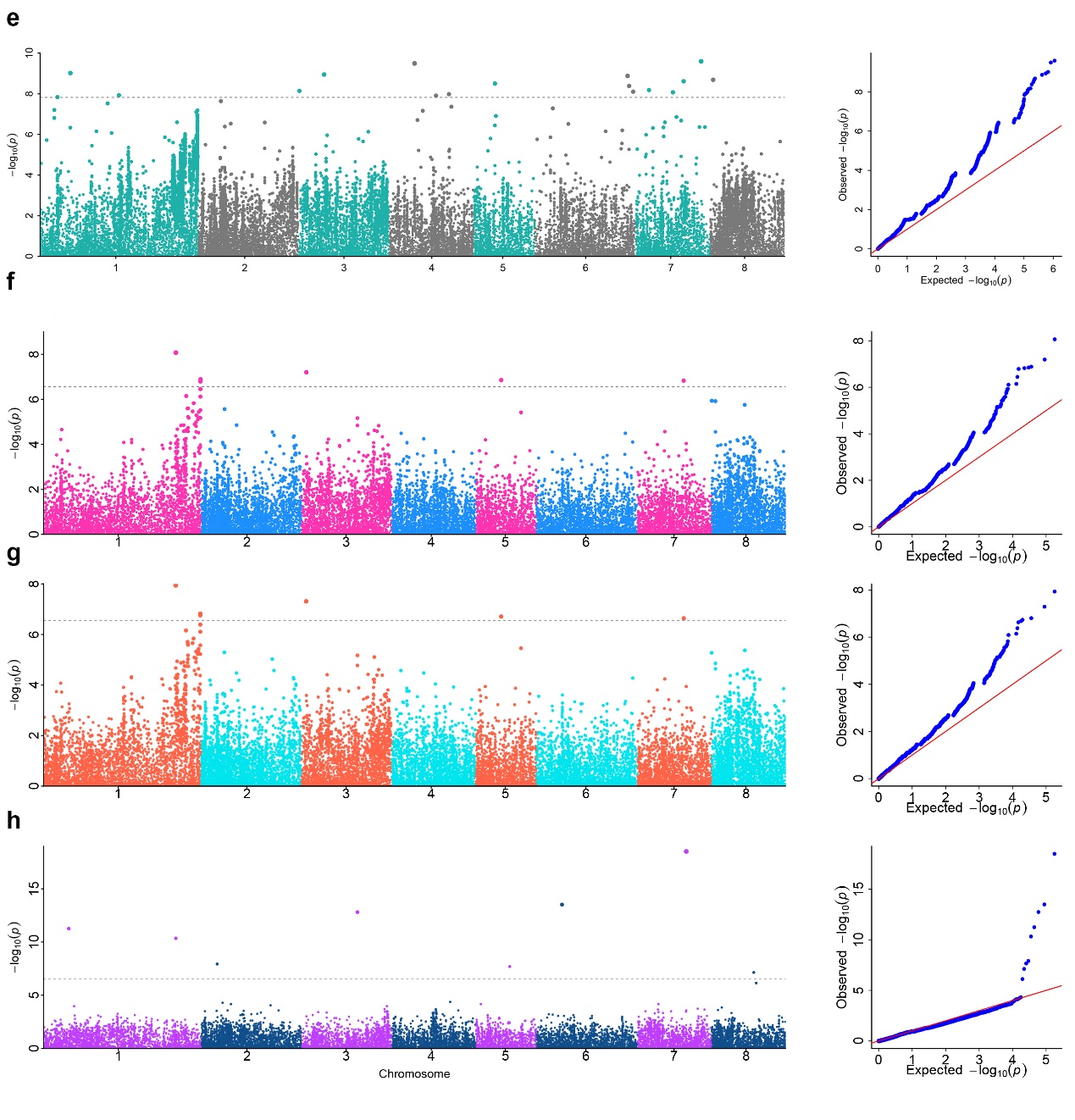

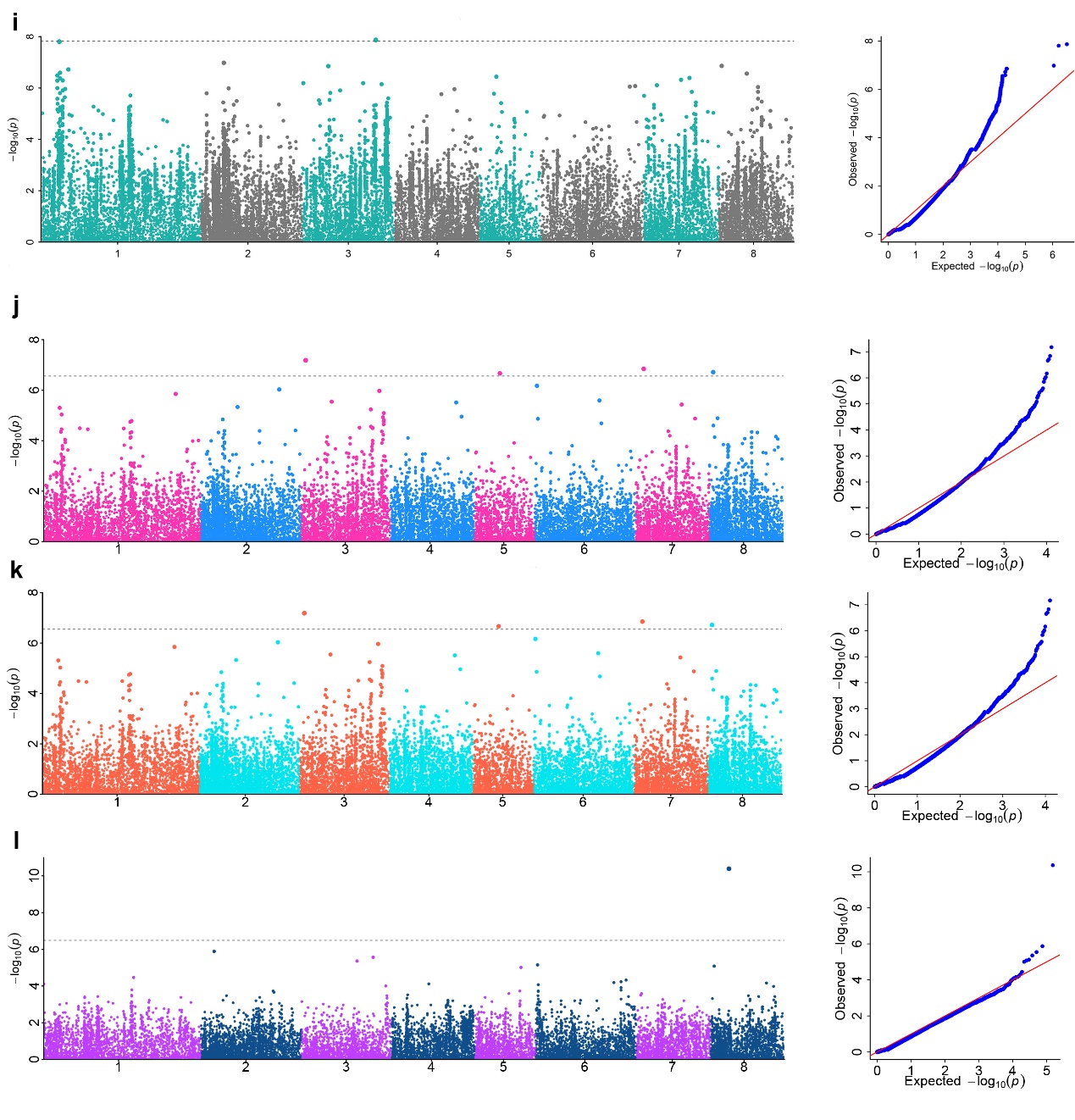


**Fig S25. Manhattan and QQ plots of genome-wide association studies (GWAS) of bloom date. a** GWAS using SNPs with MLM model in 2008. **b** GWAS using SVs with MLM model in 2008. **c** GWAS using SVs with CMLM model in 2008. **d** GWAS using SVs with FarmCPU model in 2008. **e** GWAS using SNPs with MLM model in 2016. **f** GWAS using SVs with MLM model in 2016. **g** GWAS using SVs with CMLM model in 2016. **h** GWAS using SVs with FarmCPU model in 2016. **i** GWAS using SNPs with MLM model in 2017. **j** GWAS using SVs with MLM model in 2017. **k** GWAS using SVs with CMLM model in 2017. **l** GWAS using SVs with FarmCPU model in 2017.


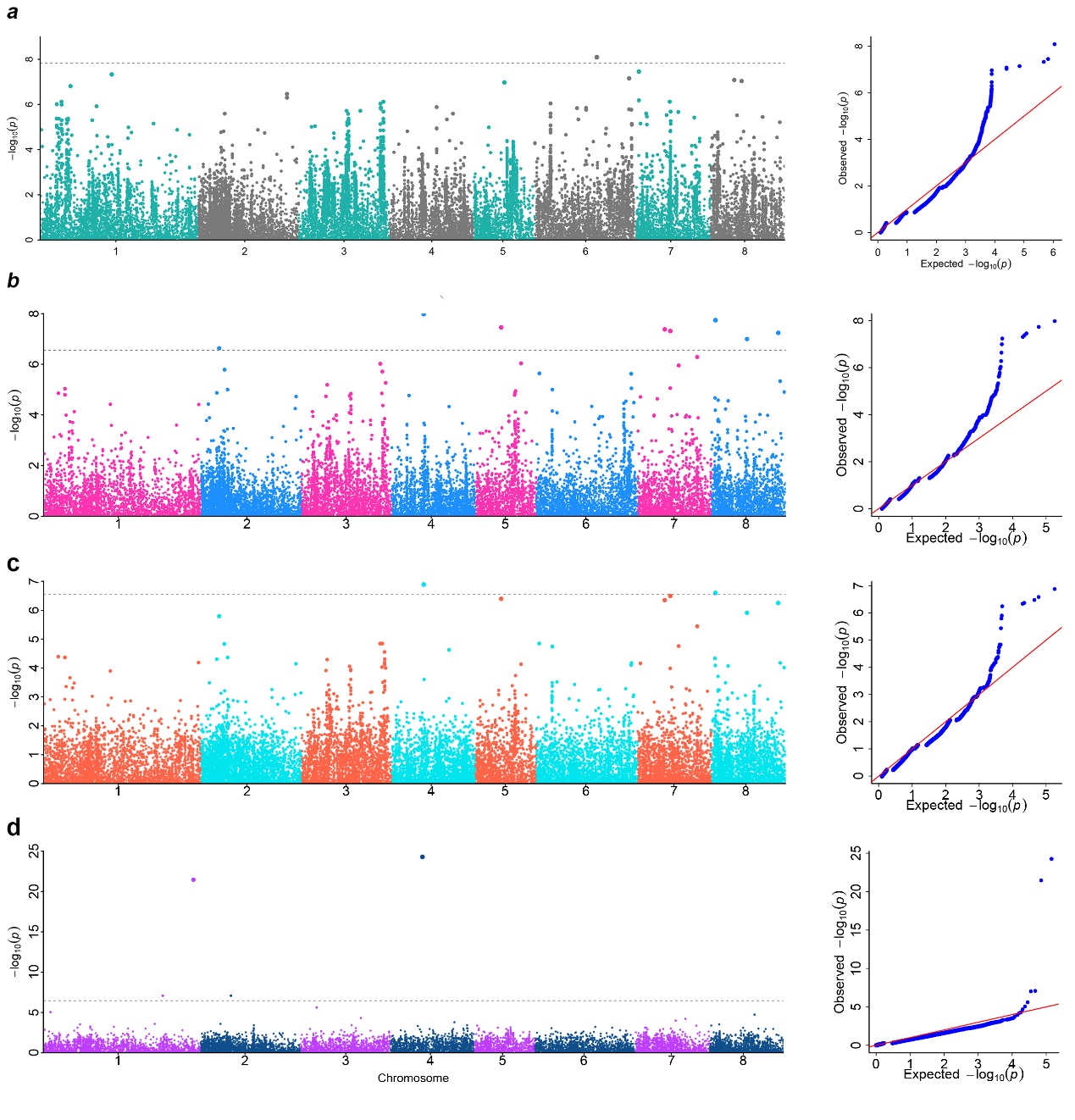

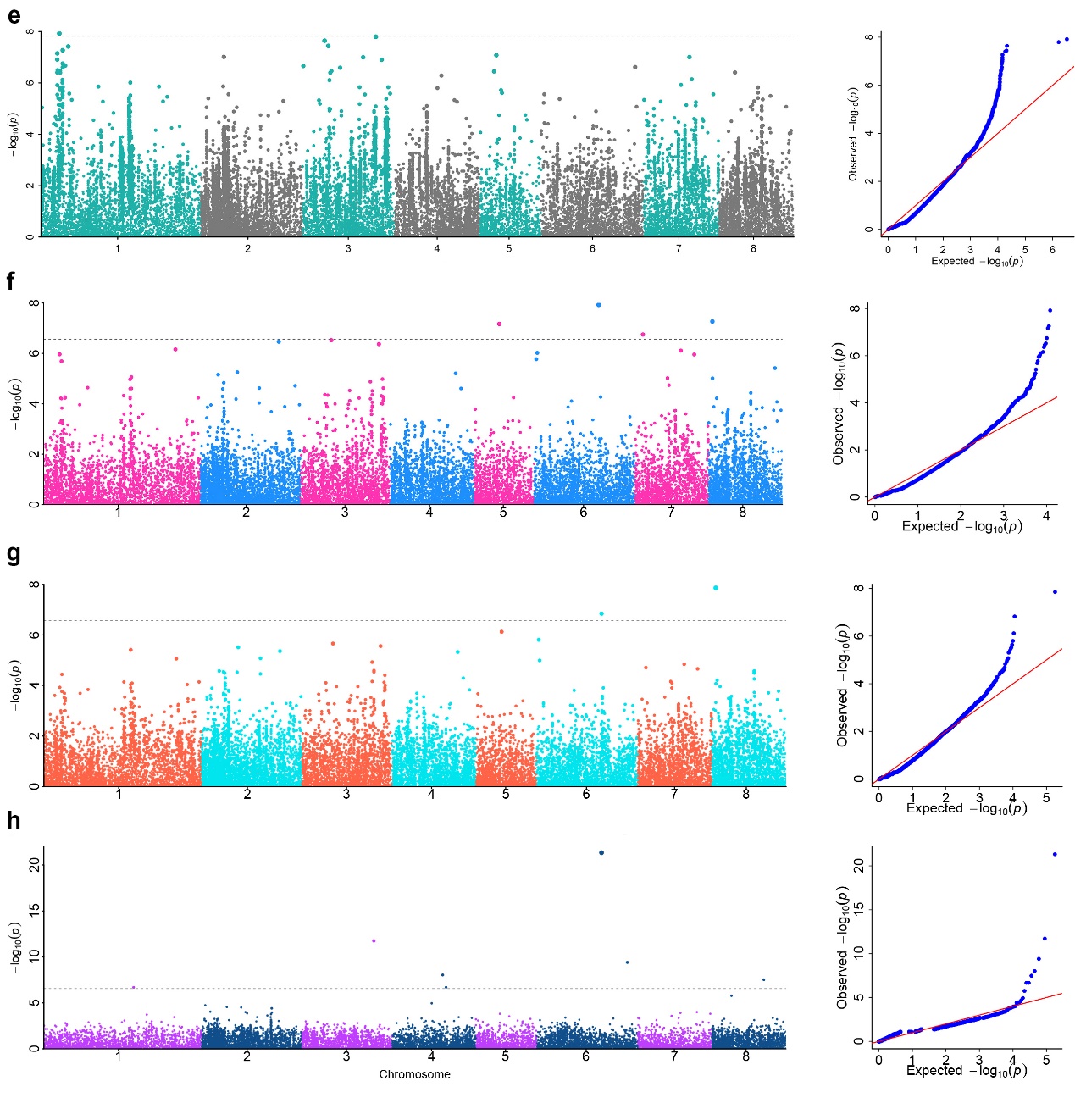


**Fig S26. Manhattan and QQ plots of genome-wide association studies on full bloom date. a** GWAS using SNPs with MLM model in 1987. **b** GWAS using SVs with MLM model in 1987. **c** GWAS using SVs with CMLM model in 1987. **d** GWAS using SVs with FarmCPU model in 1987. **e** GWAS using SNPs with MLM model in 2017. **f** GWAS using SVs with MLM model in 2017. **g** GWAS using SVs with CMLM model in 2017. **h** GWAS using SVs with FarmCPU model in 2017.


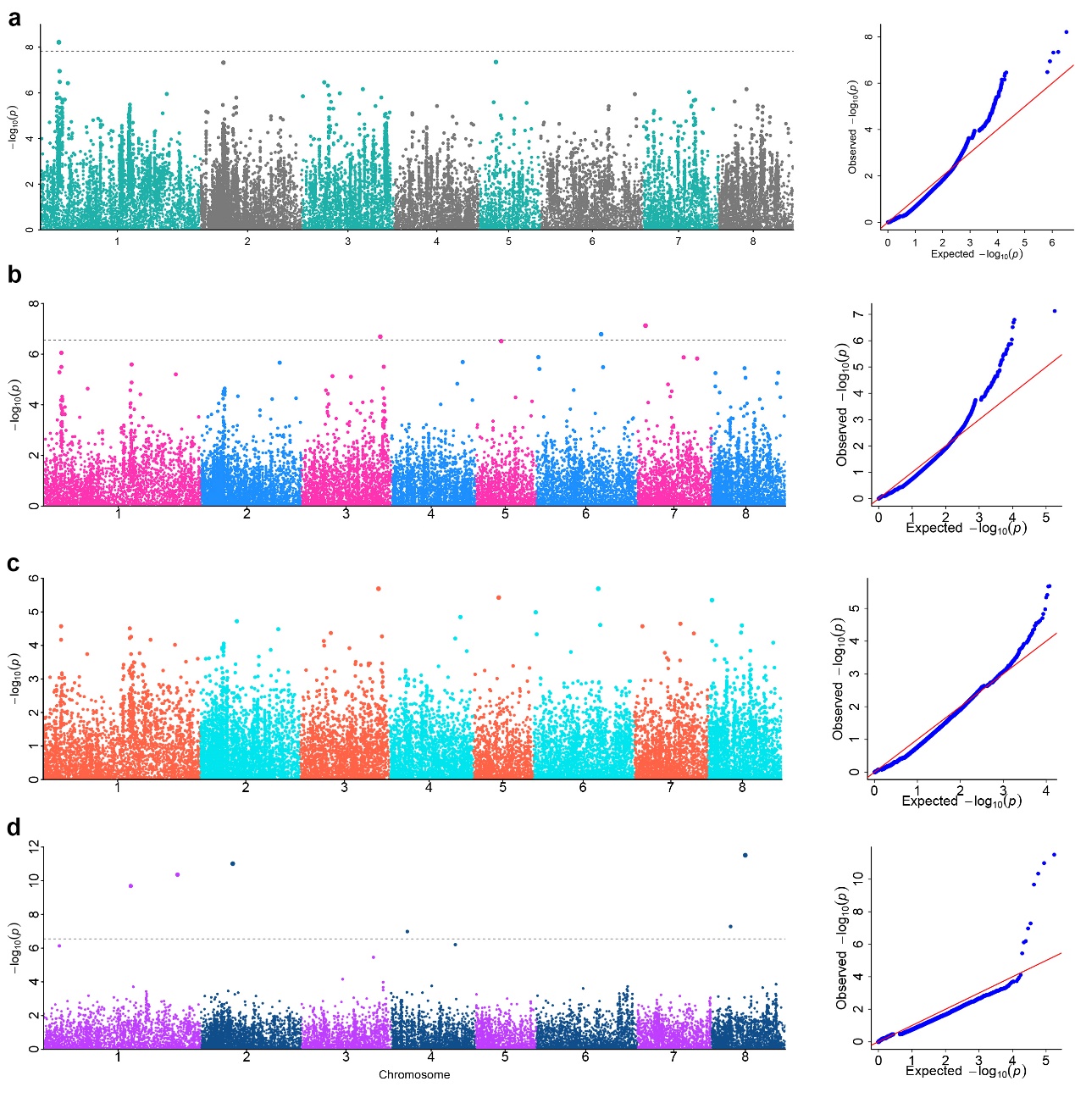


**Fig S27. Manhattan and QQ plots of genome-wide association studies (GWAS) of bloom ending date. a** GWAS using SNPs with MLM model. **b** GWAS using SVs with MLM model. **c** GWAS using SVs with CMLM model. **d** GWAS using SVs with FarmCPU model.


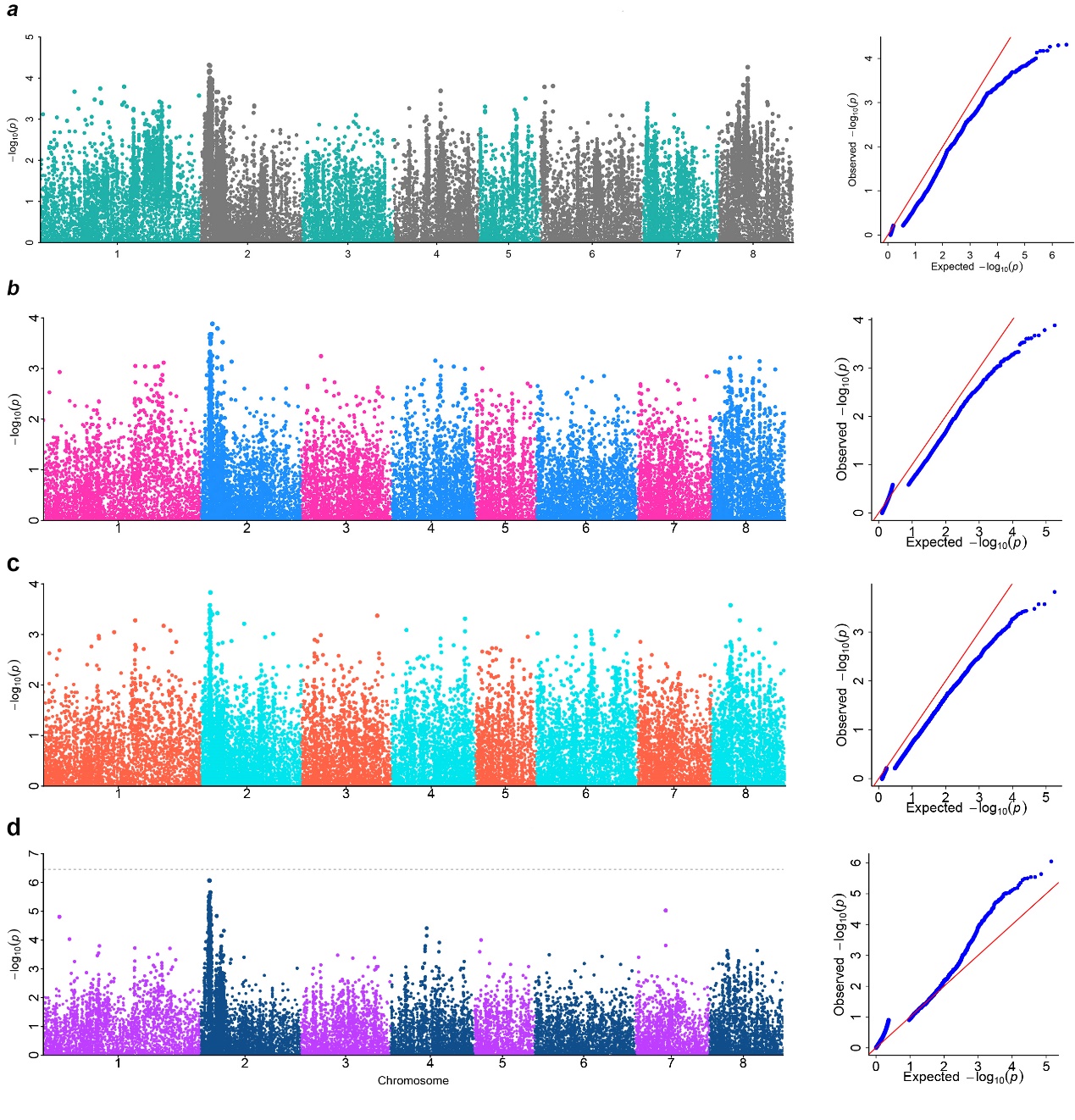

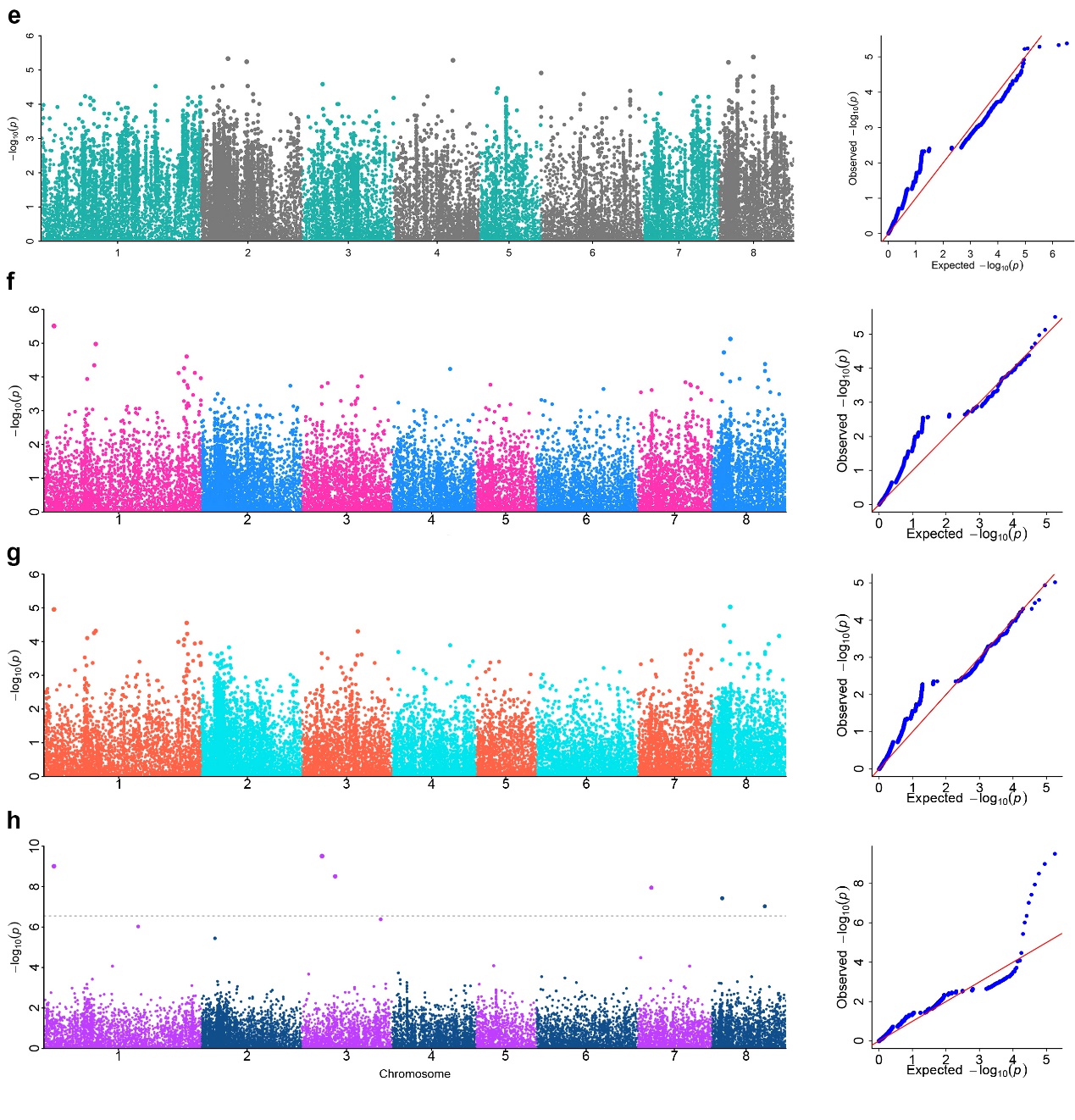


**Fig S28. Manhattan and QQ plots of genome-wide association studies (GWAS) of leaf expanding date. a** GWAS using SNPs with MLM model in 2008. **b** GWAS using SVs with MLM model in 2008. **c** GWAS using SVs with CMLM model in 2008. **d** GWAS using SVs with FarmCPU model in 2008. **e** GWAS using SNPs with MLM model in 2010. **f** GWAS using SVs with MLM model in 2010. **g** GWAS using SVs with CMLM model in 2010. **h** GWAS using SVs with FarmCPU model in 2010.


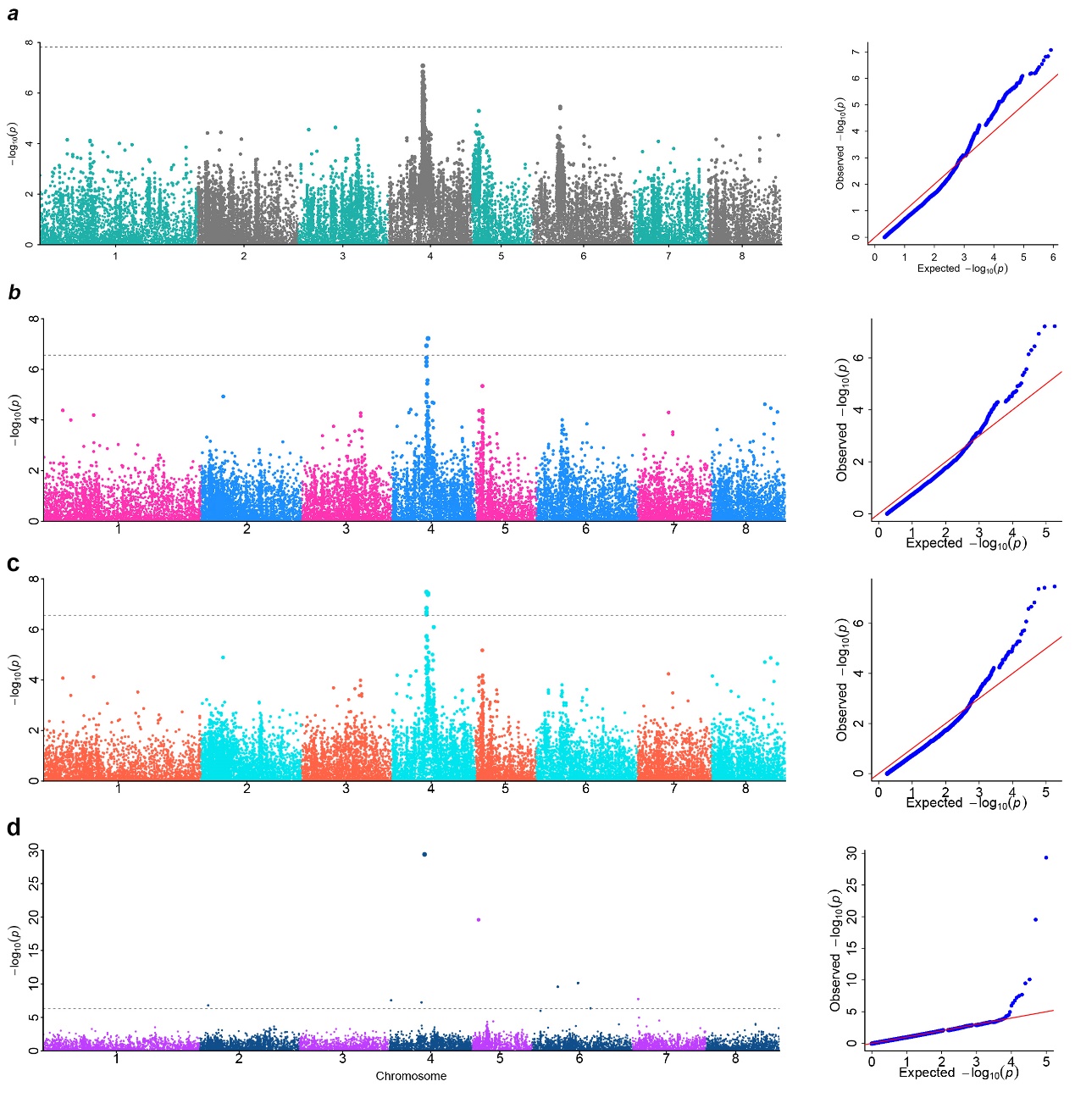


**Fig S29. Manhattan and QQ plots of genome-wide association studies (GWAS) of fruit maturation date. a** GWAS using SNPs with MLM model. **b** GWAS using SVs with MLM model. **c** GWAS using SVs with CMLM model. **d** GWAS using SVs with FarmCPU model.


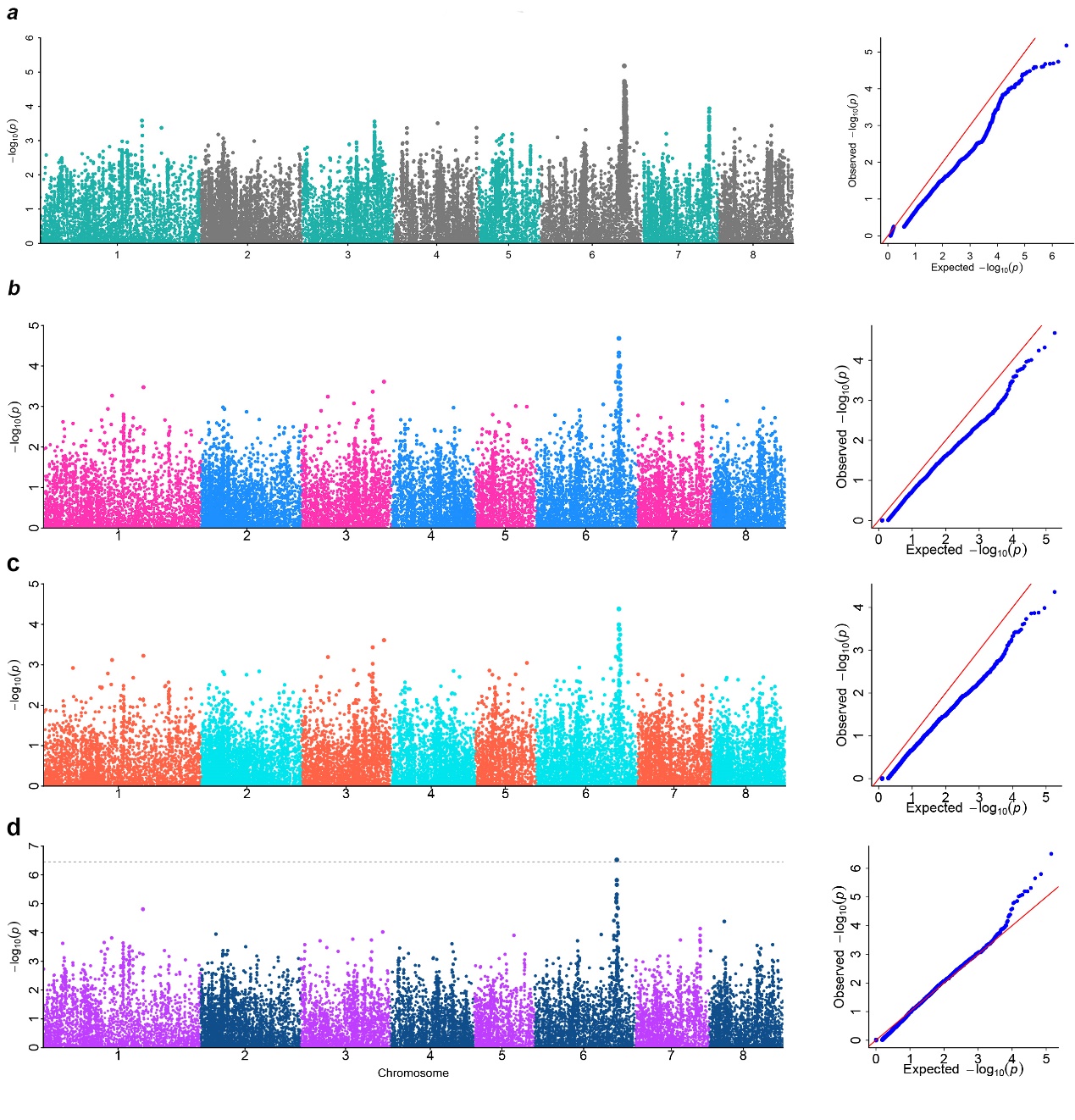

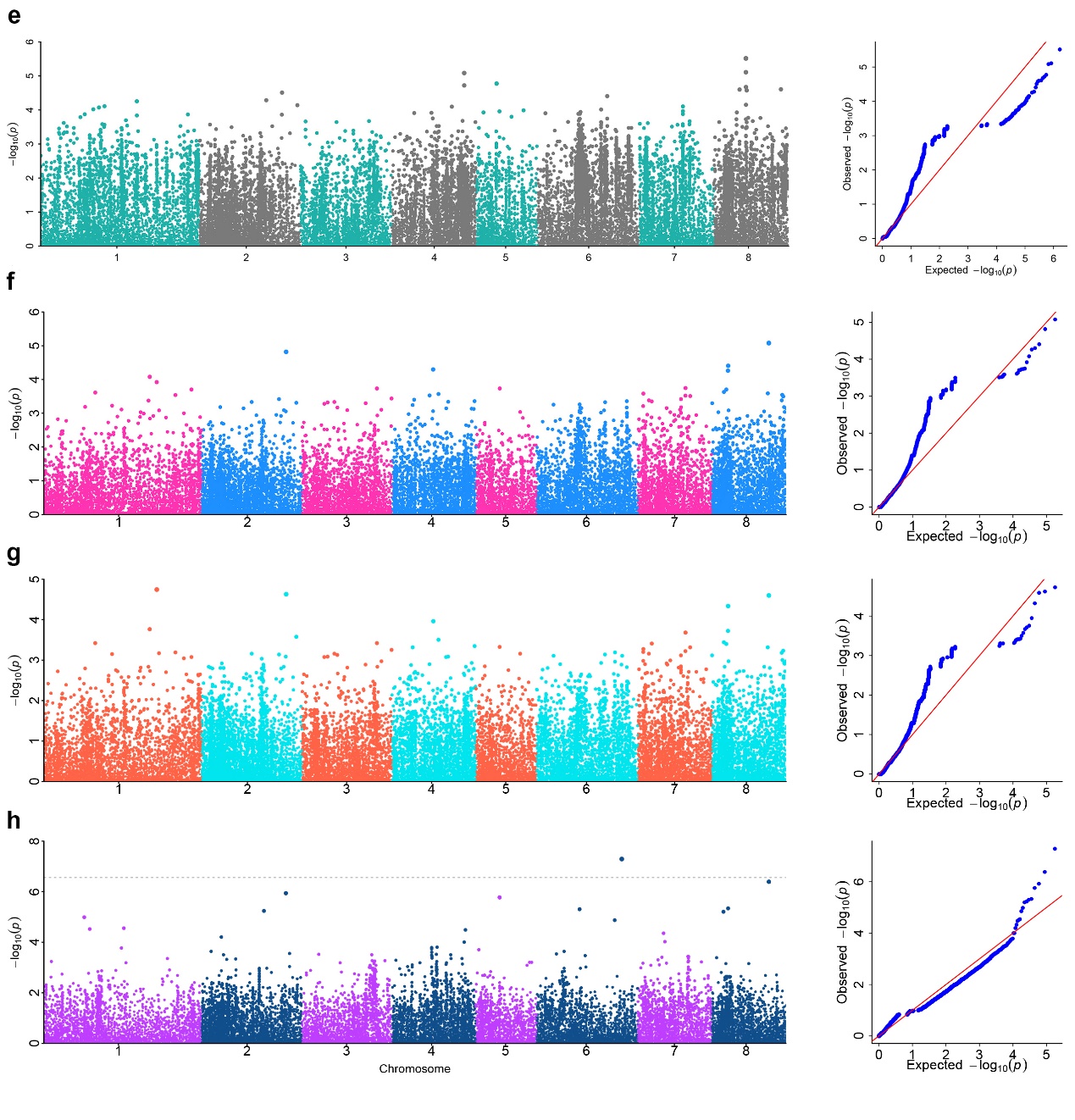


**Fig S30. Manhattan plot and QQ plots of genome-wide association studies (GWAS) of deciduous date. a** GWAS using SNPs with MLM model in 2008. **b** GWAS using SVs with MLM model in 2008. **c** GWAS using SVs with CMLM model in 2008. **d** GWAS using SVs with FarmCPU model in 2008. **e** GWAS using SNPs with MLM model in 2010. **f** GWAS using SVs with MLM model in 2010. **g** GWAS using SVs with CMLM model in 2010. **h** GWAS using SVs with FarmCPU model in 2010.


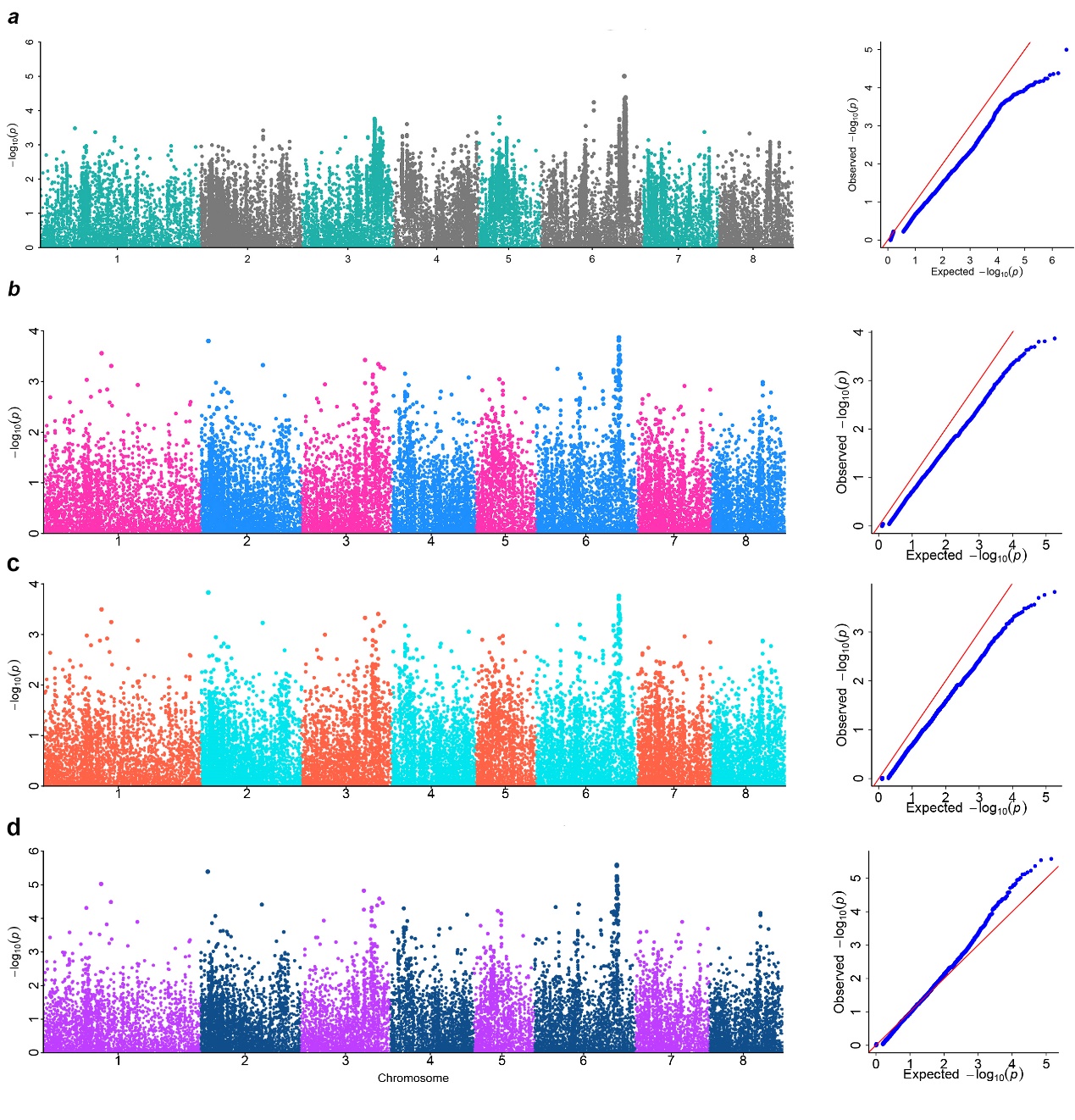

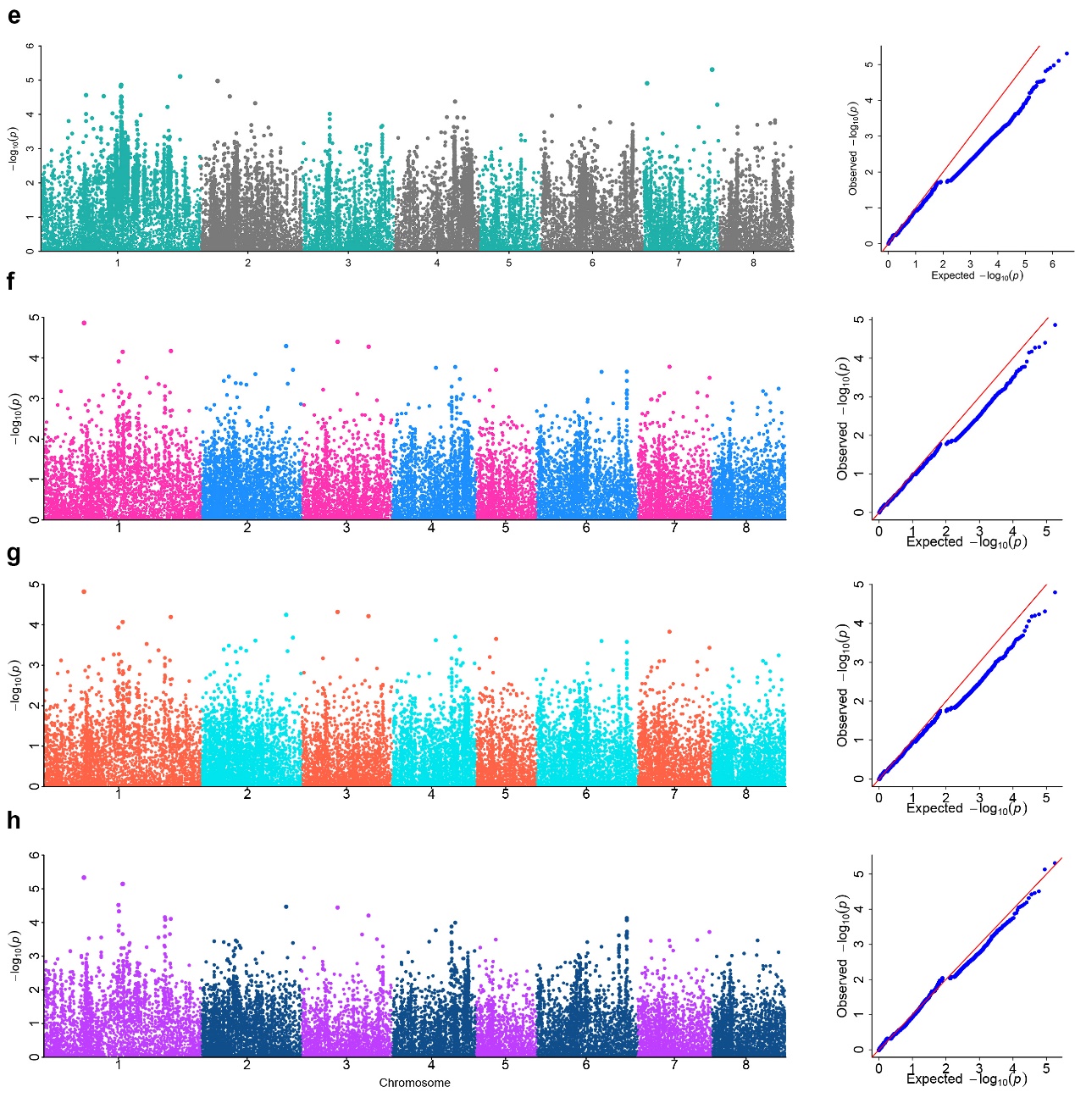


**Fig S31. Manhattan plot and QQ plots of genome-wide association studies (GWAS) of deciduous ending date. a** GWAS using SNPs with MLM model in 2008. **b** GWAS using SVs with MLM model in 2008. **c** GWAS using SVs with CMLM model in 2008. **d** GWAS using SVs with FarmCPU model in 2008. **e** GWAS using SNPs with MLM model in 2010. **f** GWAS using SVs with MLM model in 2010. **g** GWAS using SVs with CMLM model in 2010. **h** GWAS using SVs with FarmCPU model in 2010.


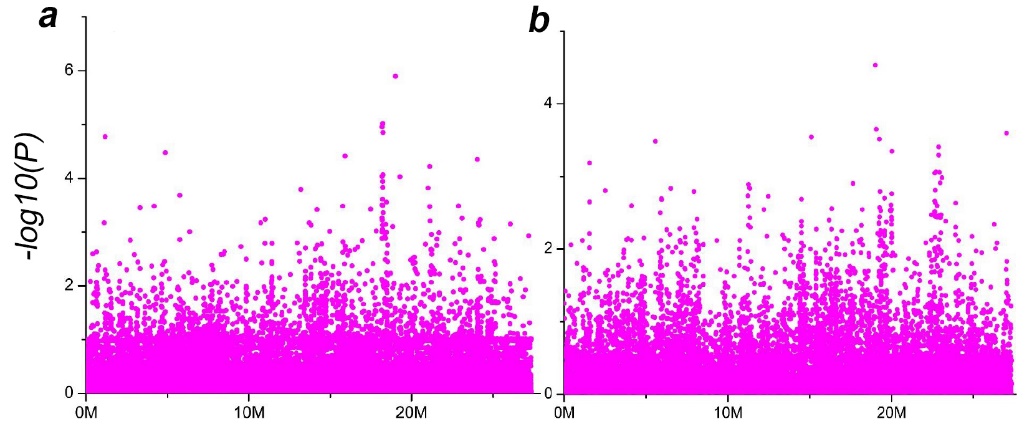


**Fig S32. Manhattan plot of genome-wide association studies (GWAS) of fruit skin color and fruit flesh color trait. a** Manhattan plot of fruit skin color on chromosome 3. **b** Manhattan plot of fruit flesh color on chromosome 3.


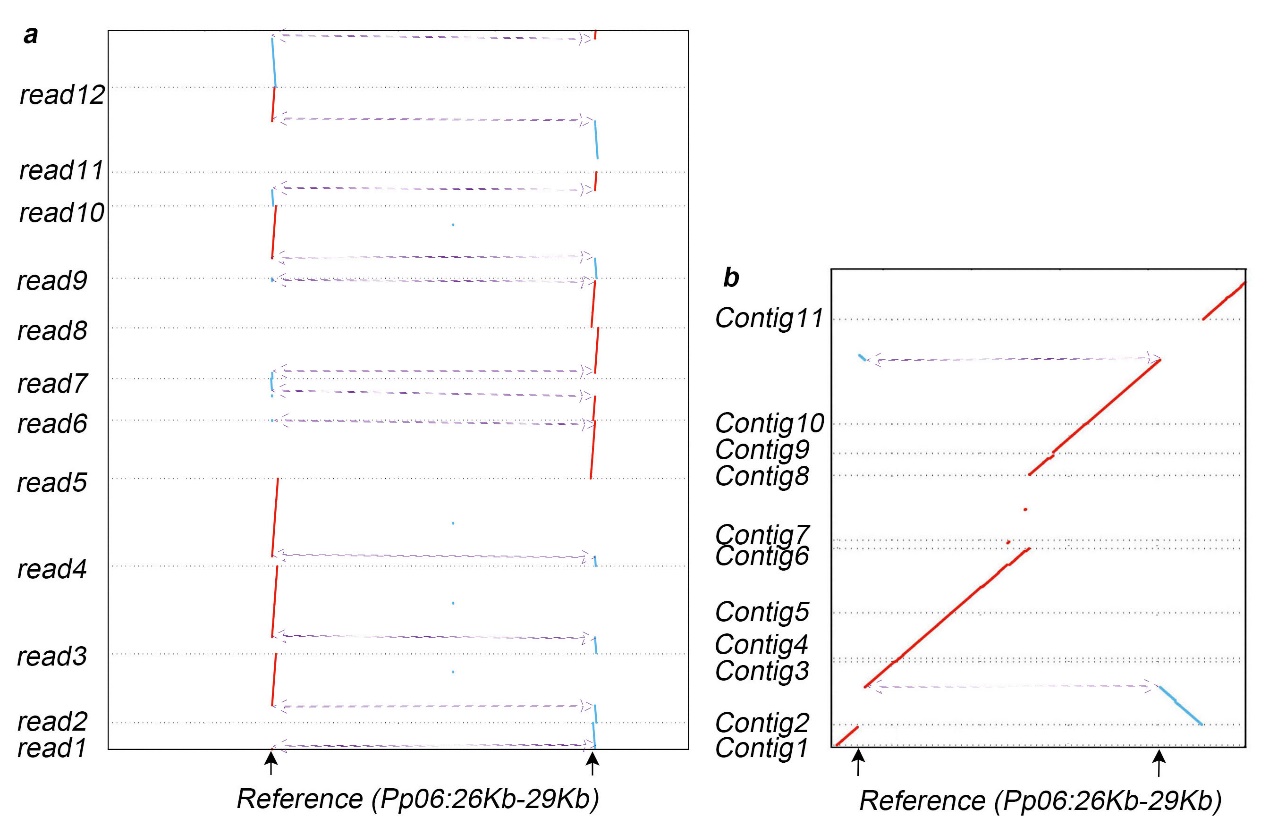


**Fig S33. Dot plot showing alignments around the inversion breakpoints. a** Dot plot showing alignments of PacBio reads to the reference genome. **b** Dot plot showing alignments of assembly contigs to the reference genome. The two black arrows indicate the inversion breakpoints. Dotted purple arrows link the same reads (**a**) or contigs (**b**) with one end mapped around one breakpoint (shown in blue) and the other end mapped around the other breakpoint (shown in red).


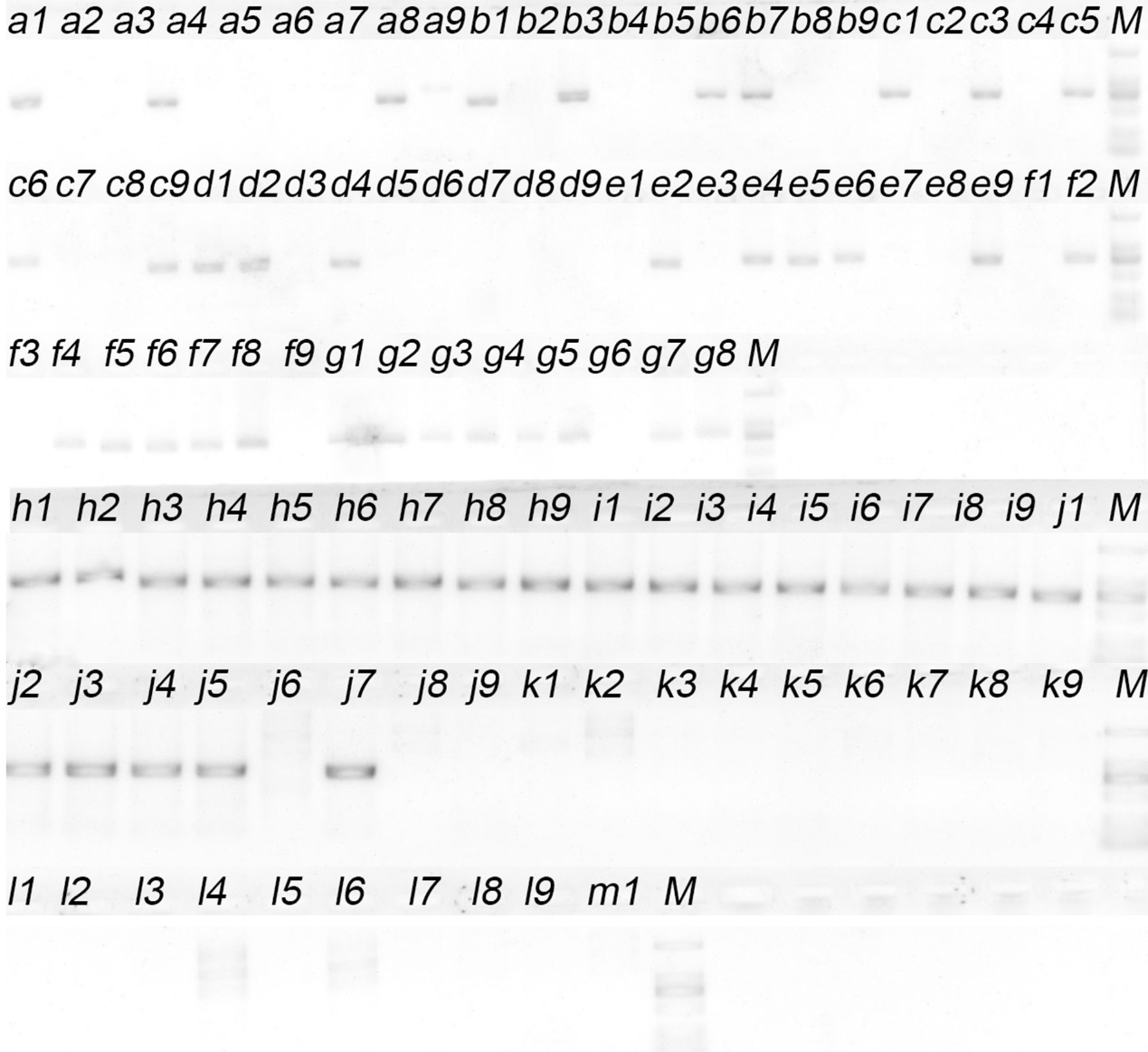


**Fig S34. Validation of the inversion in an F1 population (‘Okubo’ x ‘You Pan Tao 1-3’) and different cultivars using a PCR-based method.** The black band indicates the presence of the inversion and the corresponding cultivars are flat peach. The marker (M) shown here represents 2 kb. a-g represent F1 population and h-m are cultivars.


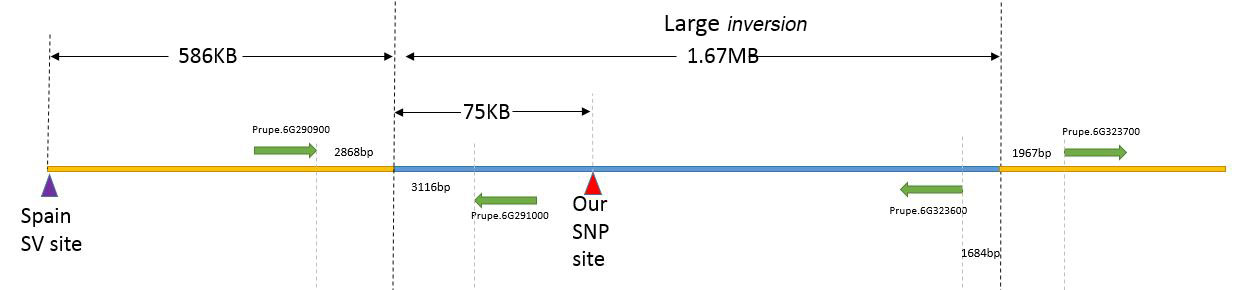


**Fig S35. Diagram illustrating the structure of the flat shape *S* locus.** The purple and red triangles indicate the 10-kb deletion (Lopez-Girona *et al.*, 2017) and the SNP (Cao *et al.*, 2016), respectively, reported previously for flat shape. The light-blue horizontal bar indicates the 1.67-Mb inversion.


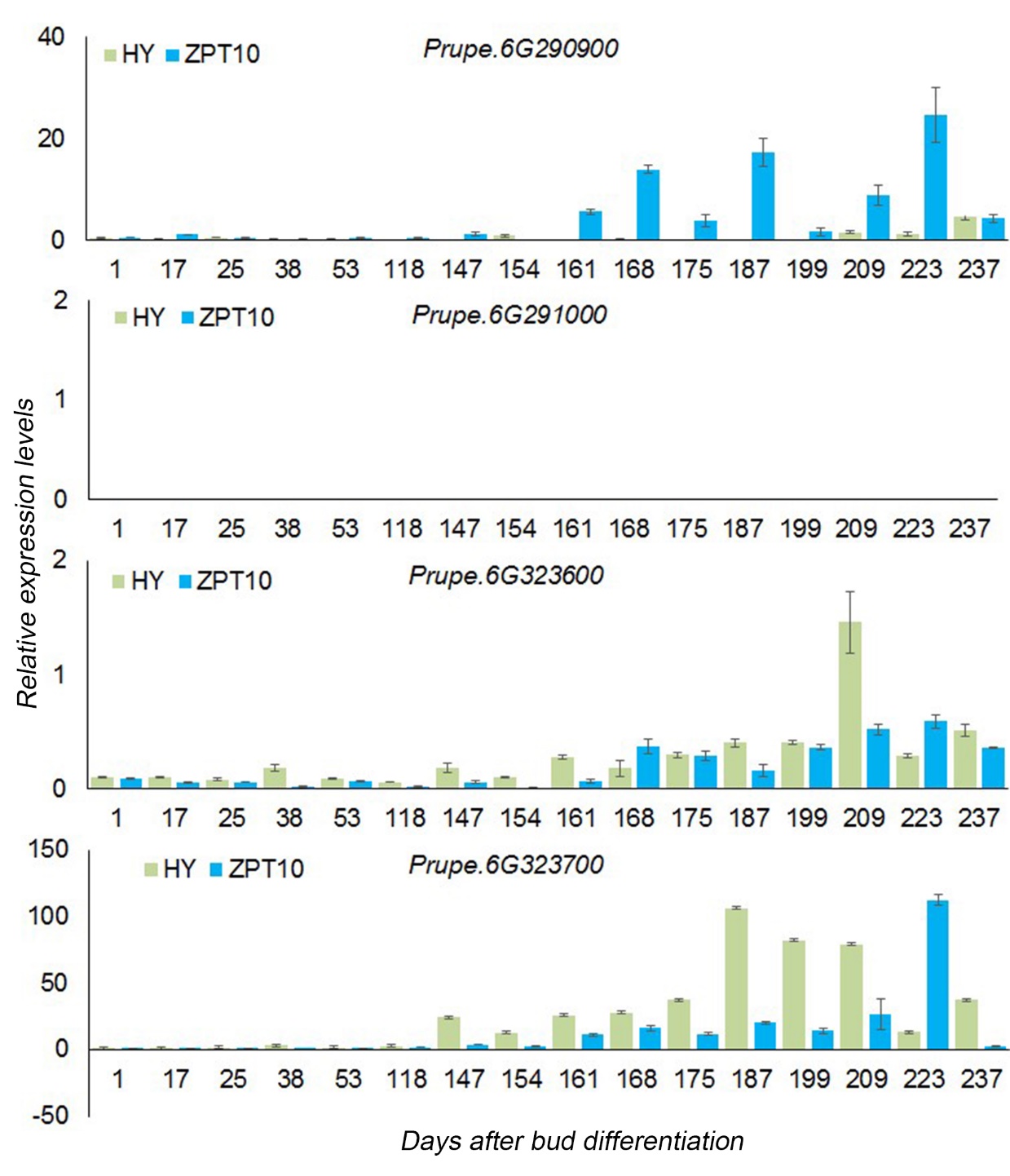


**Fig S36. Relative expression of flat shape candidate genes during peach flower bud or fruit development.** Samples were collected from the flower bud differentiations stage to fruit maturation. HY is round peach, while ZPT10 is flat. Relative expression levels were determined by qRT-PCR.


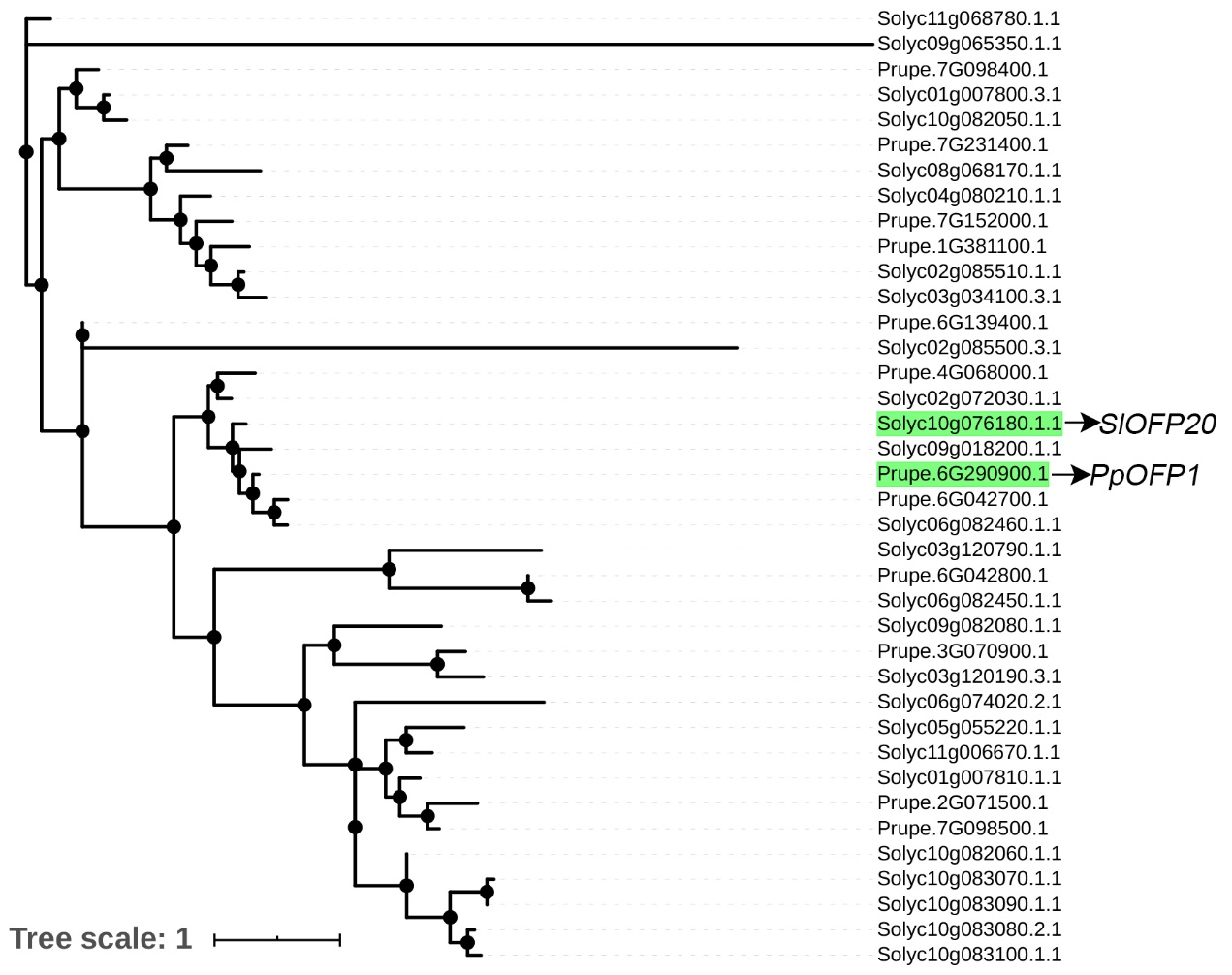


**Fig S37. Phylogenetic tree of OFP genes in peach and tomato.**

**
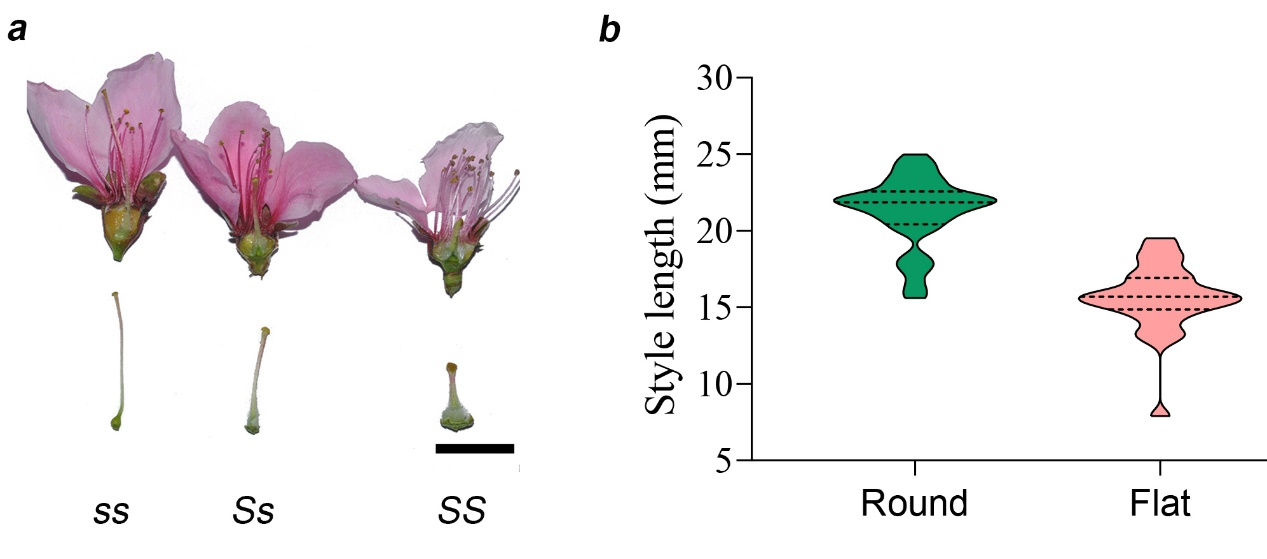
**

**Fig S38. Phenotypes of flower shapes among normal flat (Ss), aborted flat (SS) and round (ss) peach. a** comparison of style and ovary among normal flat (Ss), aborted flat (SS) and round (ss) peach. **b** Differences in style length between flat and round peach using 66 peach accessions (26 flat peach, 40 round peach). Scale bar = 0.5cm.
