# Supplementary material for: An integrated peach genome structural variation map uncovers genes associated with fruit traits
Source: Genome Biol. 2020 Oct 6;21:258. doi: 10.1186/s13059-020-02169-y (PMC7539501; doi:10.1186/s13059-020-02169-y)
Supplement: Supplementary file 3 — Additional file 3. Review history. [file 13059_2020_2169_MOESM3_ESM.docx]

Review History

**First round of review**

**Reviewer 1**

**Are you able to assess all statistics in the manuscript, including the appropriateness of statistical tests used?** Yes, and I have assessed the statistics in my report.

**Comments to author:**

Manuscript of "An integrated peach genome structural variation map uncovers genes associated with fruit traits" (GBIO-D-20-00406) performed GWAS to reveal candidate genes related to 26 fruit traits combining SVs and SNPs. Which is a further genetic population study after SNP-based GWAS (ref. 19) peach breeding history (ref. 20) and domestication study on peach (ref. 49). The results will provide meaningful understanding of genetic variation and identifying gene resources for future peach improvement. Overall, the article is well organized, the evidence is reliable and confident. However, some issues still need to be declared and improved:

Line 56 in page 4, more description should be added here (text and figure). Fig.1a listed four subgroups, while three subgroups were only showed in Fig.1b and 1c? I also didn't know which samples should be regarded as subgroup 1, which should be subgroup 2, and which should be subgroup 3.

Line 33 in page 4, read depth diversity (RDV) is the truly reflection of structural variant (SV), and different SV detection softwares utilize different info. types (or labels) from bam file. Thus, different SV types should be comprehensively considered to use appropriate methods, and used known or validated SV to examine the performance of software. Further, author should provide detailed parameters of bwa in Methods part.

Line 17 in page 5, "the main variant class was also indel", what did author want to say? I think it is meaningless. Sequencing data type, mapping and detection software, these will decide the number of different SV types.

Line 21 in page 5, rare allele accounts for most of variants whether SNP or InDel, and they have been reported to associate with important phenotypes, such disease in human. Author should be carefully define the minor allele frequency (Is it powerful using MAF < 0.01?), and pay more attention to the rare allele.

Line 40,42 in page 4, "quality value (> 20)" and "supporting reads (>5)", is it optimal? Author should give some statistics and evaluation. For example, when quality < 20, what is these SV and what regions did they distribute on? Need add an upper limit for the supporting read depth? It should be reasonable for these cutoffs.

Line 27 in page 5, "density of duplications in centromere regions was relatively higher than in other genome regions". First, a writing modification, a "that" should be added followed by "than"? Second, centromere region is a very special region in genome, which is gene-poor and repeat element-rich region. Thus, more hypothesis and validation should be description about higher density of duplications.

Line33-48 in page 5, it is too random to make it convincing that only using one sample to perform validation of SV detection based on long reads.

Line 51 in page 7, "MAF > 0.01. A total of 113,949 SVs",  "A final set of 202,273" "a total of 132,114 SVs had a minor allele frequency (MAF) < 0.01", what's the relationship between them?  

Line 13 in page 8, "two methods", what's the two methods? Two dataset?

Line 7 and 15 in page 8, it is inconsistence for "8 qualitative traits" and "10 qualitative traits"?

Line 2 in page 12, inversion and SNP, who is the main effect factor? Deeper exploration is required.

Line 54 in page 13, authors adding the resequencing data, and the final depth of each sample is 25 fold coverage. Have you tested the most appropriate sequencing depth for SV calling in peach?

Line 52 in page 14, Authors should carefully explained the details of SV calling. How to define the 'same' SVs from different peach accessions? How about the SVs containing different break points? What about the overlap SVs for each accessions? How do you deal with the SVs without break points, will they be considered twice because of the confused location? All of the SV types used in this population for performing GWAS should be illustrated clearly.

Line 29 in page 17, did you test multiple models for GWAS in GAPIT? MLM is the optimal for peach? As we known, CMLM model is more powerful than MLM, and FarmCPU model can reduce false positive caused by population structure and kinship.

Line 54 in page 17, the authors used GAPIT in this manuscript. Have you tested the other software to perform GWAS? In your previous study, TASSEL mixed linear model was used for peaches. Do they have any consistence or difference or different software?

There are only four types of variations in Fig. 2a. More details should be explained why do you discards other SV types? Furthermore, mutations of large size will be false positive.
Line 5 in page 21. Why did the authors set the MAF less than 0.01, is it the same for the SNPs sued for GWAS? Have you tested the number of SNPs under different MAF? Could you explain more about the details?

How about the variances of this peach population explained by PCA in Fig. 1b?

Supplementary materials. How did you deal with the traits, which didn't present consistent for different method. For example, the trait of suture depth showed different significant loci by SVs and SNPs.

For the experimental part,

The transient overexpression and gene silencing of PpMYB10.1 in peach could be conducted to validate the gene function.

In the previous study (ref. 19), one SNP mutation causes the difference of fruit shape from flat to round. In this manuscript, it is a 1.67 Mb inversion. Do they have any connection, or interaction?

**Reviewer 2**

**Are you able to assess all statistics in the manuscript, including the appropriateness of statistical tests used?** Yes, and I have assessed the statistics in my report.

**Comments to author:**

The work by Guo and co-authors describes structural variants in a panel of 336 peach accession and their association with agronomic traits. For some they provide further layers of experimental validation. The article is interesting, timely, and well written.

My reservations relate to the data deposition, public availability, and coding reproducibility. I could not find any information on where the data are available. Illumina reads and pacbio data should be made available as well as the files on which the results and conclusions are derived, including VCF or similar with SV coordinates relative to the genome reference. The former should be made available in NCBI SRA. The latter could be hosted by Zenodo or similar dataset database. I also reccomend the authors to provide more information about the code used in their analysis (methods are rather limited in the details provided), this could also made available in a public repository or even better in GitHub.

The pacbio assembly used to validate some SVs should be described and made available. The process of validation should be described with more details that I could find the methods section.

It would also be important to report the SV LD in the panel and how it compares to that of SNPs.

It is not clear how the association analysis accounted for population structure.

**Authors Response**

**Point-by-point responses to the reviewers’ comments:**

**Reviewer #1**

Manuscript of "An integrated peach genome structural variation map uncovers genes associated with fruit traits" (GBIO-D-20-00406) performed GWAS to reveal candidate genes related to 26 fruit traits combining SVs and SNPs. Which is a further genetic population study after SNP-based GWAS (ref. 19) peach breeding history (ref. 20) and domestication study on peach (ref. 49). The results will provide meaningful understanding of genetic variation and identifying gene resources for future peach improvement. Overall, the article is well organized, the evidence is reliable and confident. However, some issues still need to be declared and improved:

1. Line 56 in page 4, more description should be added here (text and figure). Fig.1a listed four subgroups, while three subgroups were only showed in Fig.1b and 1c? I also didn't know which samples should be regarded as subgroup 1, which should be subgroup 2, and which should be subgroup 3.

**Response**: We thank the reviewer for pointing this out and apologize for the confusion. In this study, four groups were defined according to their phenotypes and historical records, including the wild relatives, ornamental cultivars, landraces and improved cultivars, which is consistent with the phylogenetic analysis (Fig. 1c). The original Fig. 2b, which shows the separation of the wild relatives, has been moved to the supplementary file. Fig. 2b in the revised manuscript now shows the two-dimensional PCA plot without including the wild relatives, which show the separation between landraces and improved accessions, and the clustering of the majority of ornamental and landrace accessions into one group. The population structure further supported that ornamental and landraces were in the same group. Therefore, we regarded these accessions as three major groups for the downstream GWAS analysis. We have made this clear in the revised manuscript (Line 95-96).

2. Line 33 in page 4, read depth diversity (RDV) is the truly reflection of structural variant (SV), and different SV detection softwares utilize different info. types (or labels) from bam file. Thus, different SV types should be comprehensively considered to use appropriate methods, and used known or validated SV to examine the performance of software. Further, author should provide detailed parameters of bwa in Methods part.

**Response**: We thank the reviewer for bringing this up. Yes, we agree with the reviewer that RDV is the truly reflection of SV. In our study, the four different tools used for SV detection integrate at least two different types of evidence from bam files (with one tool, LUMPY, indeed using read depth diversity in SV detection): Delly (version 0.7.8) and Manta (version 1.6.0) integrate read-pair and split-read information. LUMPY (version 0.2.13) integrates read-pair, split-read and read depth signals. GRIDSS (version 2.5.2) combines assembly, read-pair and split-read evidence to detect SVs. Results from these SV detection tools were further combined and SVs detected by at least two tools were kept for the downstream analyses. To validate the performance, we checked previously reported trait-related SVs and found all of them were correctly captured in our SV dataset. For example, we identified the TE insertion variant for fruit hairiness (ref. 15), the 70.5-Kb deletion for fruit flesh texture (ref. 10), the 990-bp deletion for double flower shape (ref. 16) and the 1.8-Kb deletion for pendulous branches (ref. 17). Furthermore, we assessed our identified SVs using PacBio long reads from six different accessions, which revealed an accuracy rate of 88%.

The bwa parameters have been added in the Methods part (Line 375-376).

3. Line 17 in page 5, "the main variant class was also indel", what did author want to say? I think it is meaningless. Sequencing data type, mapping and detection software, these will decide the number of different SV types.

**Response**: Thanks for pointing this out. We have deleted this sentence in the revised manuscript.

4. Line 21 in page 5, rare allele accounts for most of variants whether SNP or InDel, and they have been reported to associate with important phenotypes, such disease in human. Author should be carefully define the minor allele frequency (Is it powerful using MAF < 0.01?), and pay more attention to the rare allele.

**Response**: Here we just described the distribution of SV under different MAF values. For example, there were 131,075 SVs with MAF < 0.01, 173,434 SVs with MAF < 0.05 and 182,599 SVs with MAF < 0.1 (Supplementary Fig. 1). Most of studies focusing on SV analysis used the parameters of MAF > 0.05, such as in cucumber (ref. 8), maize (ref. 12) and Rapeseed (ref. 32). In our present study, only 7 of 336 peach accessions showed weeping branch trait, which indicated that the casual allele for this trait should have MAF less than 0.05. Therefore, we kept SVs with MAF > 0.01 in our GWAS analysis. For human, some diseases have been reported to be caused by rare alleles; however, very limited studies on rare alleles have been reported in plants. While we agree with the reviewer that rare alleles are important in plants, we think the analysis of the rare alleles is beyond the scope of this manuscript.

5. Line 40,42 in page 4, "quality value (> 20)" and "supporting reads (>5)", is it optimal? Author should give some statistics and evaluation. For example, when quality < 20, what is these SV and what regions did they distribute on? Need add an upper limit for the supporting read depth? It should be reasonable for these cutoffs.

**Response**: We are sorry for the confusion here. These two parameters were only used in SV detection using the LUMPY program, not the other three tools. We made this clear in the revised manuscript (Line 87). According to a previous SV study (ref. 21), we set the quality value >20 to reduce false positives. In addition, we have further evaluated the accuracy of SV with quality value <20 in our study, which revealed the accuracy rate was only around 20%, compared to 88% accuracy rate of the final SV set. Most of the SVs had supporting reads <100 and we believed it would not affect our results without setting the upper limit for supporting reads. Although some SVs (very few as our average depth of sequencing is ~20x) could be unidentified with the parameter ‘supporting reads >5’ in LUMPY (while in the same time this increased the accuracy of SVs detected by LUMPY), they could be recovered by the other three tools if they are real.

6. Line 27 in page 5, "density of duplications in centromere regions was relatively higher than in other genome regions". First, a writing modification, a "that" should be added followed by "than"? Second, centromere region is a very special region in genome, which is gene-poor and repeat element-rich region. Thus, more hypothesis and validation should be description about higher density of duplications.

**Response**: We thank the reviewer for bringing this up. The abundant transposable elements in the centromere region might lead to higher density of duplication. However, it is also possible that high repeat contents in the centromere region could lead to false short read mapping, resulting in higher rate of falsely identified duplications. Nonetheless, we think this finding is not important therefore we have deleted this sentence and the associated figure (Fig. 2a) in the revised manuscript.

7. Line33-48 in page 5, it is too random to make it convincing that only using one sample to perform validation of SV detection based on long reads.

**Response**: In the revised manuscript, we have used PacBio reads from additional five accessions to evaluate the SV detection, which revealed an accuracy rate of 88%.

8. Line 51 in page 7, "MAF > 0.01. A total of 113,949 SVs", "A final set of 202,273" "a total of 132,114 SVs had a minor allele frequency (MAF) < 0.01", what's the relationship between them?

**Response**: We thank the reviewer for pointing this out. We have further checked the original VCF files and counted the variants. The final set of 202,273 SVs (>50 bp) included 131,075 SVs (not 132,144) with MAF < 0.01 and 71,198 SVs with MAF >0.01. However, for GWAS analysis, we also used the 109,067 small indels (<50 bp, MAF > 0.01) detected using the GATK pipeline. Thus, the number 113,949 was wrong and it should be 180,265. We have made this clear in the revised manuscript (Line 172-173).

9. Line 13 in page 8, "two methods", what's the two methods? Two dataset?

**Response**: Thank you for pointing this out. Yes, it means two datasets. We have corrected this in the revised manuscript.

10. Line 7 and 15 in page 8, it is inconsistence for "8 qualitative traits" and "10 qualitative traits"?

**Response**: Thanks for pointing this out. A total of 11 qualitative traits were analyzed in this study, of which 8 were targeted in the previous GWAS analysis using SNPs (ref19, ref20), including fruit shape, fruit hairiness, flesh color (white/yellow), flesh color around the stone, flower shape (double/single; showy/non-showy), pollen sterility and kernel taste. Three of the 11 qualitative traits were newly studied here, including pendulous branch, hypanthium color (white/yellow) and anther color. We are sorry that “10 qualitative traits” is a typo error; should be 11. We have made this clear in the revised manuscript (Line 185-186).

11. Line 2 in page 12, inversion and SNP, who is the main effect factor? Deeper exploration is required.

**Response**: We thank the reviewer for bring this up. The SNP reported in the previous study (ref. 19) is in the fifth intron of *Prupe.6G292200* and does not affect the coding sequence. We did characterize the potential role of *Prupe.6G292200* in regulating fruit shape by transferring  *Prupe.6G292200* into tomato. We found that expression of *Prupe.6G292200* in tomato did not affect the fruit shape (data not shown), strongly supporting that *Prupe.6G292200* was not the underlying gene although the SNP is highly associated with fruit shape. On the other hand, *Prupe.6G290900* adjacent to the inversion has been validated to regulate fruit shape and its expression pattern was highly consistent with the genotype (Fig. 5). Therefore, we believed that the inversion should be the main effect factor.

12. Line 54 in page 13, authors adding the resequencing data, and the final depth of each sample is 25 fold coverage. Have you tested the most appropriate sequencing depth for SV calling in peach?

**Response**: Yes, we have tested the appropriate sequencing depth for SV calling in peach. We selected one peach accession (sample38) which was sequenced to up to 50x depth and randomly separated the reads to obtain the data with different read depths, including 5x, 10x, 20x, 30x, 40x and 50x. The results showed that more SVs were identified with the increase of sequencing depth while the accuracy was reduced after 20x (please check Fig. R1 below). These results were consistent with the findings reported in the LUMPY SV detection tool, which shows that the sensitivity is lower at read depth < 10x and the false positives are increasing along with the increase of read depth (ref. 23). Therefore, by considering both sensitivity and false discoveries, we determined to generate data of around 20x. We don’t think this analysis provide much useful information and thus decided to not include the above analysis results in the revised manuscript.

Figure R1. Comparison of SV detection using data of different sequencing depth.

13. Line 52 in page 14, Authors should carefully explain the details of SV calling. How to define the 'same' SVs from different peach accessions? How about the SVs containing different break points? What about the overlap SVs for each accession? How do you deal with the SVs without break points, will they be considered twice because of the confused location? All of the SV types used in this population for performing GWAS should be illustrated clearly.

**Response**: Thanks for your suggestion. We have added the details of SV calling in the revised manuscript. In this study, we merged the SV call set of each accession using ‘SURVIVOR merge 1000 1 1 1 0 50’ command. Same SVs were defined by the parameter ‘1000’, which means the maximum distance between breakpoints. Therefore, the same type of SVs containing different breakpoints within 1000 bp would be considered as same SVs. SVs without breakpoints would not be considered twice, if their locations were within 1000 bp. We have made this clear in the revised manuscript (Line 386-388).

14. Line 29 in page 17, did you test multiple models for GWAS in GAPIT? MLM is the optimal for peach? As we known, CMLM model is more powerful than MLM, and FarmCPU model can reduce false positive caused by population structure and kinship.

Response: We thank the reviewer for the suggestion. We have further performed GWAS using CMLM and FarmCPU models implemented in GAPIT. The GWAS results were largely identical between MLM and CMLM models. While the FarmCPU model reported much less significant sites in a specific QTL locus, the most significant sites detected by the FarmCPU model were almost the same as those detected by the MLM and CMLM models (please check Supplementary Fig. 5-31, Supplementary Table 12). We have added one sentence to summarize this finding in the revised manuscript (Line 175).

15. Line 54 in page 17, the authors used GAPIT in this manuscript. Have you tested the other software to perform GWAS? In your previous study, TASSEL mixed linear model was used for peaches. Do they have any consistence or difference or different software?

**Response**: Thanks for the suggestion. We have further performed GWAS using TASSEL for three traits, fruit shape, flesh color around stone and fruit maturity date. GWAS results from GAPIT and TASSEL were largely the same (please check Fig. R2 below). In addition, the GAPIT has integrated the PCA and kingship analysis, which made it much easier and more convenient to run the pipeline. Therefore, we selected the GAPIT to perform GWAS in this study. We think analysis using TASSEL did not provide novel information so we did not include the results in the revised manuscript.

Figure R2. GWAS of three traits using GAPIT and TASSEL tools.

16. There are only four types of variations in Fig. 2a. More details should be explained why do you discards other SV types? Furthermore, mutations of large size will be false positive.

**Response**: We are not sure the meaning of “discards other SV types” as Fig. 2a shows all the four types of SVs identified in this study (Please also check Supplementary Table 2). Nonetheless, Fig. 2a has been removed in the revised manuscript (please refer to our response to question 6), this making this point moot.

We agree with the reviewer that large mutations are more likely to be false positives. However, we have integrated four different SV detection tools, which increased the SV accuracy. For example, a large inversion of 1.67 Mb was identified and validated in the flat fruit shape analysis.

17. Line 5 in page 21. Why did the authors set the MAF less than 0.01, is it the same for the SNPs used for GWAS? Have you tested the number of SNPs under different MAF? Could you explain more about the details?

**Response**: Most of studies focusing on SV analysis used the parameters of MAF < 0.05, such as in cucumber (ref. 8), maize (ref. 12) and Rapeseed (ref. 32). In our present study, only 7 of 336 peach accessions showed weeping branch trait, which indicated that the casual allele for this trait should have MAF less than 0.05 and greater than 0.01. Therefore, we filtered the variants using MAF < 0.01. We have tested the number of SNPs under different MAF, which showed the same pattern as SVs (please check Fig. R3 below).

Figure R3. Distribution of SNPs based on the site-frequency spectrum.

18. How about the variances of this peach population explained by PCA in Fig. 1b?

**Response**: Thanks for pointing this out. The Fig. 1b has been moved to supplementary figures. The variance explained by each principal component has been added in this figure.

19. Supplementary materials. How did you deal with the traits, which didn't present consistent for different method. For example, the trait of suture depth showed different significant loci by SVs and SNPs.

**Response**: For GWAS of suture depth using SNPs, the top four most significant SNPs were located at Pp03: 16118017, Pp03: 16118023, Pp03: 16118011 and Pp03: 16118036. Around these loci, we checked the SVs and found out that there were no SVs covering these four SNPs, which might explain why there were no signals in GWAS using SVs. In this case, we could not determine which was the casual variant and we considered both of the GWAS results using SNPs and SVs.

20. The transient overexpression and gene silencing of PpMYB10.1 in peach could be conducted to validate the gene function.

**Response**: The function of *PpMYB10.1* in anthocyanin biosynthesis in peach has already been validated (ref. 42,43,44). Therefore, we think it’s not needed to perform these assays in this study.

21. In the previous study (ref. 19), one SNP mutation causes the difference of fruit shape from flat to round. In this manuscript, it is a 1.67 Mb inversion. Do they have any connection, or interaction?

**Response**: First we would like to point out that the SNP is highly associated with different fruit shapes, as reported in ref. 19, instead of causing different fruit shapes. This SNP is located in the 1.67 Mb inversion region, and both the SNP and the inversion co-segregated with flat/round fruit shapes. Please check our response to question 11 above for more related information.

**Reviewer #2:**

The work by Guo and co-authors describes structural variants in a panel of 336 peach accession and their association with agronomic traits. For some they provide further layers of experimental validation. The article is interesting, timely, and well written.

1. My reservations relate to the data deposition, public availability, and coding reproducibility. I could not find any information on where the data are available. Illumina reads and pacbio data should be made available as well as the files on which the results and conclusions are derived, including VCF or similar with SV coordinates relative to the genome reference. The former should be made available in NCBI SRA. The latter could be hosted by Zenodo or similar dataset database. I also recommend the authors to provide more information about the code used in their analysis (methods are rather limited in the details provided), this could also made available in a public repository or even better in GitHub.

**Response**: We thank the reviewer for the valuable suggestions. We have deposited the Illumina reads and PacBio data in NCBI SRA under accession PRJNA630113, and have now made the data public. The VCF files were deposited in Zenodo (https://zenodo.org/record/3953424). The scripts used in this study were available in GitHub (<https://github.com/ytguojian/Peach_SV>). We have added this information in the revised manuscript.

2. The pacbio assembly used to validate some SVs should be described and made available. The process of validation should be described with more details that I could find the methods section.

**Response**: We are sorry for the confusion here. In this study we did not assemble PacBio reads; therefore, we don’t have PacBio assembly that can be made available. As described in our original manuscript (last paragraph of the first Results section), the validation was performed by manually checking the bam files of PacBio long read mapping using IGV, and comparing the mapping with the identified SVs for consistency. We have made this clear in the revised manuscript (Line 111-115 and Line 408-410)

3. It would also be important to report the SV LD in the panel and how it compares to that of SNPs. 
**Response**: We thank the reviewer for the suggestion. We have calculated the LD values using SNPs and SVs. The LD values calculated by SVs were much lower than those calculated by SNPs, which could be explained by the much lower resolution of the SV data (average distance between adjacent SVs was much larger than that between adjacent SNPs, thus lower correlation). However, the LD decay calculated from the SNP and SV data show a similar pattern (see Fig. R4 below) and the distances over which LD decayed to half of its maximum values calculated using SNPs or SVs were both around 20 Kb, consistent with that reported in a previous study (ref. 18). We don’t think this analysis provide much useful information and thus decided to not include the above analysis results in the revised manuscript. However, if the editor and the reviewer still feel that this is necessary we will be happy to comply.

Figure R4. Decay of LD in all samples calculated by SNPs and SVs.

4. It is not clear how the association analysis accounted for population structure.

Response: In GWAS analyses, the PCA and kinship analyses have been integrated in GAPIT (ref. 64), in which we set PCA value to 3. We have added this information in methods in the revised manuscript (Line 450).

**Second round of review**

**Reviewer 1**

Manuscript of 'An integrated peach genome structural variation map uncovers genes associated with fruit traits' (GBIO-D-20-00406R1) performed GWAS to reveal candidate genes related to 26 fruit traits combining SVs and SNPs, and provides meaningful understanding of genetic variation and identifying gene resources for future peach improvement. The authors have made a lot of improvement, and this revision fully addresses the points raised in review. There is just one point to be incorporated into the final draft.
1.     Please label the major groups in Fig.1b and 1c, which will be clear for readers to understand the accessions that each group represent.
2.     Supplementary Table 2. Modify the word of 'istribution' in the header of the table to 'distribution'.
3.     Numbers in the LD plot in Fig. 3a is not clear enough.

**Reviewer 2**

The authors addressed all my comments.

**Authors Response**

**Point-by-point responses to the reviewers’ comments:**

Reviewer #1:

1.Please label the major groups in Fig.1b and 1c, which will be clear for readers to understand the accessions that each group represent.

*Answer: Thanks for your suggestion. We have added the labels for the major groups.*

2.Supplementary Table 2. Modify the word of 'istribution' in the header of the table to 'distribution'.

*Answer: Thank you for pointing this out. We have revised this error.*

3.Numbers in the LD plot in Fig. 3a is not clear enough.

*Answer: We thank the reviewer for pointing this out. We have added the scale bar for the different LD value and modified the word size for LD blocks.*
